# Extracellular Proton Concentrations Impacts LN229 Glioblastoma Tumor Cell Fate *via* Differential Modulation of Surface Lipids

**DOI:** 10.3389/fonc.2017.00020

**Published:** 2017-03-01

**Authors:** Sebastian John, K. C. Sivakumar, Rashmi Mishra

**Affiliations:** ^1^Disease Biology Program, Department of Neurobiology and Genetics, Rajiv Gandhi Centre for Biotechnology, Thiruvananthapuram, India; ^2^Distributed Information Sub-Centre, Rajiv Gandhi Centre for Biotechnology, Thiruvananthapuram, India

**Keywords:** extracellular pH dynamics, glycosphingolipid conformational heterogeneity, glioblastoma, antitumor therapy, cholesterol, GM3, cyclophilin A, surface clustering

## Abstract

**Background:**

Glioblastoma multiforme (GBM) is a highly aggressive form of brain cancer with marginal survival rates. GBM extracellular acidosis can profoundly impact its cell fate heterogeneities and progression. However, the molecules and mechanisms that enable GBM tumor cells acid adaptation and consequent cell fate competencies are weakly understood. Since extracellular proton concentrations (pHe) directly intercept the tumor cell plasma membrane, surface lipids must play a crucial role in pHe-dependent tumor cell fate dynamics. Hence, a more detailed insight into the finely tuned pH-dependent modulation of surface lipids is required to generate strategies that can inhibit or surpass tumor cell acid adaptation, thereby forcing the eradication of heterogeneous oncogenic niches, without affecting the normal cells.

**Results:**

By using image-based single cell analysis and physicochemical techniques, we made a small-scale survey of the effects of pH ranges (*physiological*: pHe 7.4, *low*: 6.2, and *very low*: 3.4) on LN229 glioblastoma cell line surface remodeling and analyzed the consequent cell fate heterogeneities with relevant molecular targets and behavioral assays. Through this basic study, we uncovered that the extracellular proton concentration (1) modulates surface cholesterol-driven cell fate dynamics and (2) induces ‘differential clustering’ of surface resident GM3 glycosphingolipid which together coordinates the proliferation, migration, survival, and death reprogramming *via* distinct effects on the tumor cell biomechanical homeostasis. A novel synergy of anti-GM3 antibody and cyclophilin A inhibitor was found to mimic the very low pHe-mediated GM3 supraclustered conformation that elevated the surface rigidity and mechano-remodeled the tumor cell into a differentiated phenotype which eventually succumbed to the anoikis type of cell death, thereby eradicating the tumorigenic niches.

**Conclusion and significance:**

This work presents an initial insight into the physicochemical capacities of extracellular protons in the generation of glioblastoma tumor cell heterogeneities and cell death *via* the crucial interplay of surface lipids and their conformational changes. Hence, monitoring of proton–cholesterol–GM3 correlations *in vivo* through diagnostic imaging and *in vitro* in clinical samples may assist better tumor staging and prognosis. The emerged insights have further led to the translation of a ‘pH-dependent mechanisms of oncogenesis control’ into the surface targeted anti-GBM therapeutics.

## Introduction

Extracellular tumor acidification (pHe) is a well-recognized hallmark of cancer progression (1–6).

Consolidated information from several studies has brought forth the knowledge that tumor masses can demonstrate heterogeneous pHe levels ranging from 7.4 to 6.2 units, in general, that can fall as low as 5.5–3.4 units and even below (1–9). This is essentially due to several factors such as nutrient starvation, oxidative stress, hypoxia, and high glucose, which tune cancer cells to anaerobic glycolysis, resulting in a high buildup of an acid equivalent of lactate, which is then extruded to the extracellular environment generating high proton concentrations (10).

**Figure 1:**
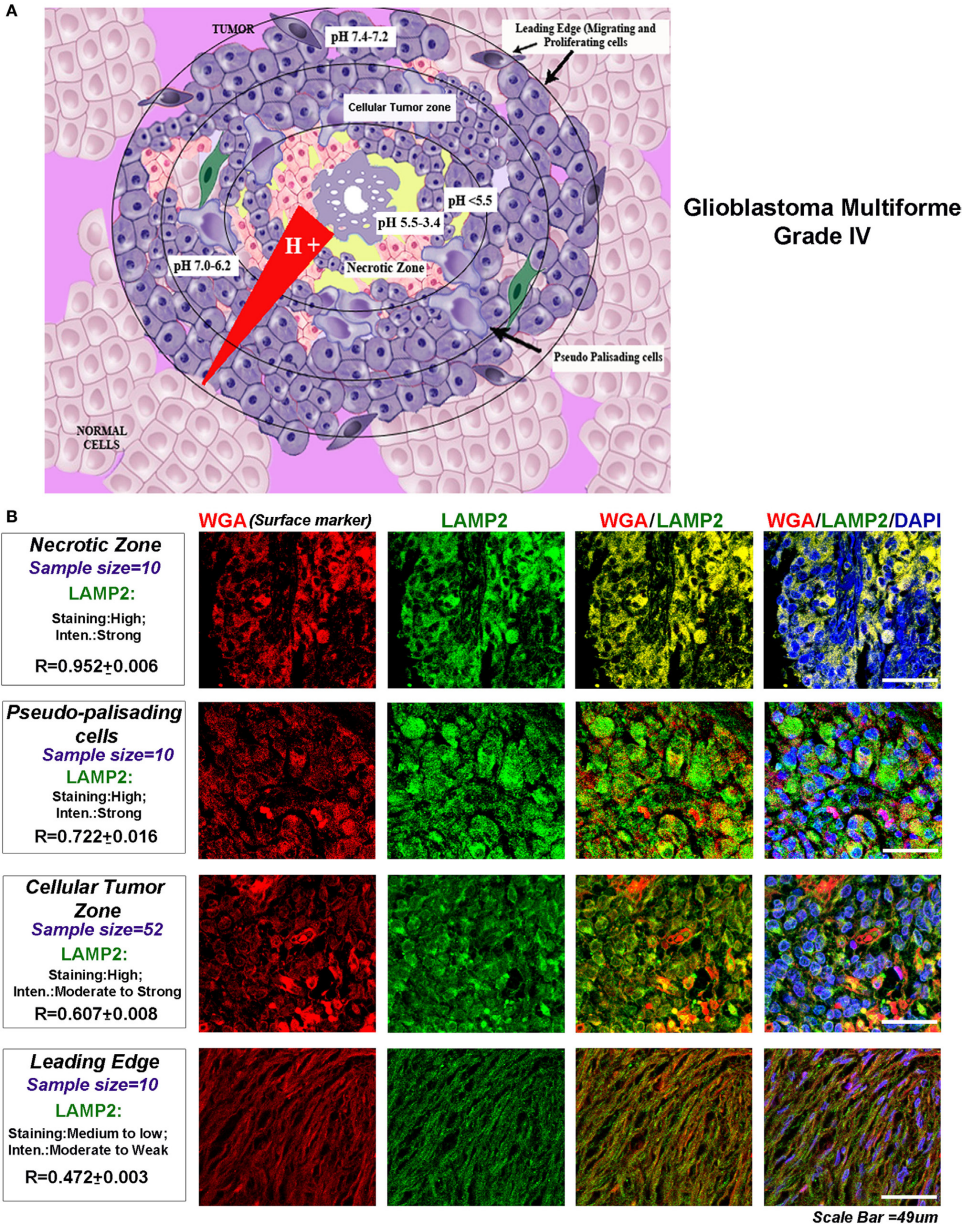
**Glioblastoma tumor zones and surface localization of LAMP2 as a biomarker of progressive acidosis in each zone**. **(A)** Diagrammatic representation of the glioblastoma tumor zones and the approximate pH ranges associated with each zone: necrotic zone (pH < 5.5–3.4), pseudo-palisading cells layer (pH approximately 5.5 or less), cellular tumor zone (pH < 7.0–6.2), and the leading edge or the tumor margins (pH < 7.4–7.2). **(B)** The glioblastoma multiforme (GBM) patient tissues were probed for surface marker wheat germ agglutinin (WGA, Alexa 598 labeled, red) and LAMP2 (an acidosis marker, green). A change in surface residency of LAMP2 in different tumor zones was estimated quantitatively by colocalization coefficient values (*R*) between WGA and LAMP2 staining. Qualitative significance was obtained by scoring the ‘Staining’ into high, medium, and low categories. ‘Intensity’ of expression was classified into strong, moderate, and weak. The number of ‘distinct GBM patient tissues’ considered for analysis of each tumor zone is indicated in the figure. Note that the average *R* values were derived from sample size for each tumor zone* three technical replicates. Also, note that there was a ‘very high’ surface localization of LAMP2 in the necrotic zones and ‘high’ localization in pseudo-palisading and cellular tumor zones. LAMP2 colocalized well with WGA in these zones, suggesting that more acidic tumor zones have high surface LAMP2 in GBMs.

The glioblastomas are characterized by four main tumor zones with distinct cellular phenotypes that are histopathologically discernable (http://glioblastoma.alleninstitute.org/, follow ‘documentation tab’ and ‘overview’ under document sub-section). Figure 1A diagrammatically represents the differential pH microenvironments that may develop in these zones. The necrotic zone shows exfoliating and dying cells. This zone is reported to be formed due to overcrowding of high metabolically active cells, which extrude protons into the external tumor milieu leading to a dramatic lowering of the microenvironmental pH (approximately pH < 5.5–3.4 and even below). The necrotic zone is circumvented by slow migrating, large, and swollen ‘Pseudo-palisading Cells’ (approximately pH < 5.5). Beyond this zone is the ‘Cellular Tumor’ zone where pH microenvironment varies from approximately 7.0 to 6.2 units, and tumor cells show various survival adaptations and cell fate heterogeneities. The well-vascularized tumor margins (approximately pH 7.4–7.2) are essentially characterized by fast migrating and invasive cells, hence known as the ‘Leading Edge.’

In acidic extracellular microenvironment (pHe), differential proton concentrations directly intercept the tumor cell surface. In the case of physiologically pH-adapted cells, such as those lining the kidney and gastric lumen, a high surface residency of cholesterol and GM3 glycosphingolipid is observed (Figure S1 in Supplementary Material) (11–13), which is probably to prevent the acid facing cellular membrane from proton-mediated hydrolysis. Interestingly, in tumor cells too, the biosynthesis of both cholesterol and GM3 is reported to be enhanced (14–17). However, how surface lipids such as cholesterol and GM3, the major components of the plasma membrane, fine tunes the tumor cell fate adaptations and heterogeneities in response to varying pHe is not yet understood.

Hence in this study, we have specifically addressed: (1) how extracellular protons can work to generate differential glioblastoma/tumor cell fates, that is, what cell fates competence is associated with which extracellular pH ranges and (2) whether extracellular proton concentrations differentially modify cholesterol and GM3 biophysical and molecular properties that can crucially impact the intratumoral cell fate heterogeneities?

Toward this, we needed to test (i) the biophysical–biochemical capacities of protons, (ii) the differential cell fates they generate, and (iii) how surface cholesterol and GM3 participate in this process. To further explore the corroboration of our *in vitro* observations with GBM pathology, we examined the GBM patient data, as distinct histopathological zones of GBMs are presumptively associated with certain pH microenvironments (diagrammatically shown in Figure 1A). This attempt was made in the light of a recently published study where presumptive low pH zones in breast cancer tissues were identified to be associated with enhanced LAMP2 surface localization *in vivo* and extracellular acidification was shown to generate the same effect *in vitro* (18). Later, the authors measured the *in vivo* tumor pH and concluded that LAMP2 enhanced expression and plasma membrane localization highlights the regions of progressive tumor acidosis (18, 19).

We too found that LAMP2 surface localization was significantly enhanced in the regions attributed with ‘very low’ (necrotic zone) and ‘low pH’ (pseudo-palisading and cellular tumor zones) in the glioblastoma patient samples (Figure 1B) [US Biomax glioblastoma tissue arrays containing 63 distinct patient samples were processed for this analysis; here, ‘*R*’ denotes the colocalization coefficient between wheat germ agglutinin (binds to cell surface marker) and LAMP2 surface localization]. Hence, to some extent, extrapolation of acidosis-driven *in vitro* observations with comparable evidences in different tumor zones of GBM patient samples may be relevant in this study and may present fresh insights into the development of the therapeutic strategies/diagnostic biomarkers from the results obtained.

## Materials and Methods

### Chemicals and Reagents

All fluorescent probes were purchased from Thermo Fisher Scientific (USA) and other common chemicals from Sigma Inc. (St. Louis, MO, USA); cyclophilin A inhibitor was from Calbiochem (Cat no-239836). LN229 cell line used here is a p53 mutant, PTEN wild type, and was purchased from ATCC, USA. It is derived from aggressive, metastatic and grade IV (WHO) glioblastoma. It is an often used GBM cell line in acidosis and other glioblastoma-related studies (20–23). Other normal and tumor cell lines used in this study were also from ATCC, USA. All antibodies used in this study are detailed in Supplementary Material.

### Molecular Simulations

A series of atomic-scale molecular dynamic simulations were carried on the lipid bilayer comprising of 16 AcGM3 and 146 POPC molecules for three different pHs: 7.4, 6.2, and 3.4. This allowed us to study the conformation and organization of the glycan headgroup of GM3 on bilayer normal at different pHs in a systematic manner. The simulation was performed using the GROMACS molecular dynamics package (for further details, please see Section ‘Methods and Materials’ in Supplementary Material) (24–27).

### Cell Culture and *In Vitro* Acidification Protocol

Briefly, cells were seeded for 16 h in DMEM (high glucose, Invitrogen, Cat No-12100-046) medium with 10% fetal bovine serum (Cat no. RM9955-500mL, HiMedia) and 1X antibiotic–antimycotic solution (HiMedia-A002A). For 8-well chamber slides, seeding density was kept at 8 × 10^4^ cells per well; for 6-well plates, initial cell density was 2.8 × 10^6^, and for 96- and 24-well plates, the seeding densities were 10^4^ and 4.8 × 10^4^, respectively. The cells were subsequently incubated in serum-free DMEM (high glucose) medium with 1X antibiotics for 2 h for serum-derived signal downregulation. The medium was then replaced with DMEM (high glucose) supplemented with 100 ng/ml of recombinant epidermal growth factor and adjusted to pH 7.4, 6.2, and 3.4. pH of the medium was adjusted with 2 N HCl. Cells were incubated at the specified pH units for 3 h, and then experimental sets were either treated with 10 µM methyl-beta-cyclodextrin (+veCD condition) or were left untreated in the respective pHs (−veCD condition). Cells were given these treatments for 2 h and were either then fixed with 1.5% PFA for immunocytochemistry or were processed for other assays as described in Supplementary Material. Each condition in the experimental sets (−veCD and +veCD) was kept in triplicate for the derivation of statistics and significance of data.

### Statistics

All above experiments were performed in triplicates. Each independent experiment had three technical replicates. Error bars are indicative of average SDs obtained across three independent experimental sets. The *p* values were obtained through Bonferroni’s *t*-test between pH 7.4 units −veCD condition and other pH conditions and were represented as *p ≤ 0.05, **p ≤ 0.01, and ***p ≤ 0.001, respectively. In individual situations, other comparisons of significance were also indicated. Image analysis was done on over 200 cells in each condition using Fiji image processing software. Calibration bar for LUT converted images were shown in the respective figures.

Please see Supplementary Material for further details on image analysis schema and other common methods used in this study.

## Results

### LN229 Glioblastoma Tumor Cells Show Differential Phenotypes and Levels of Surface Cholesterol in Response to Extracellular pH Ranges

Acid stress experiments have been successfully employed to understand cellular responses to both the intracellular (pHi) and extracellular pH (pHe) shifts (28–30).

**Figure 2:**
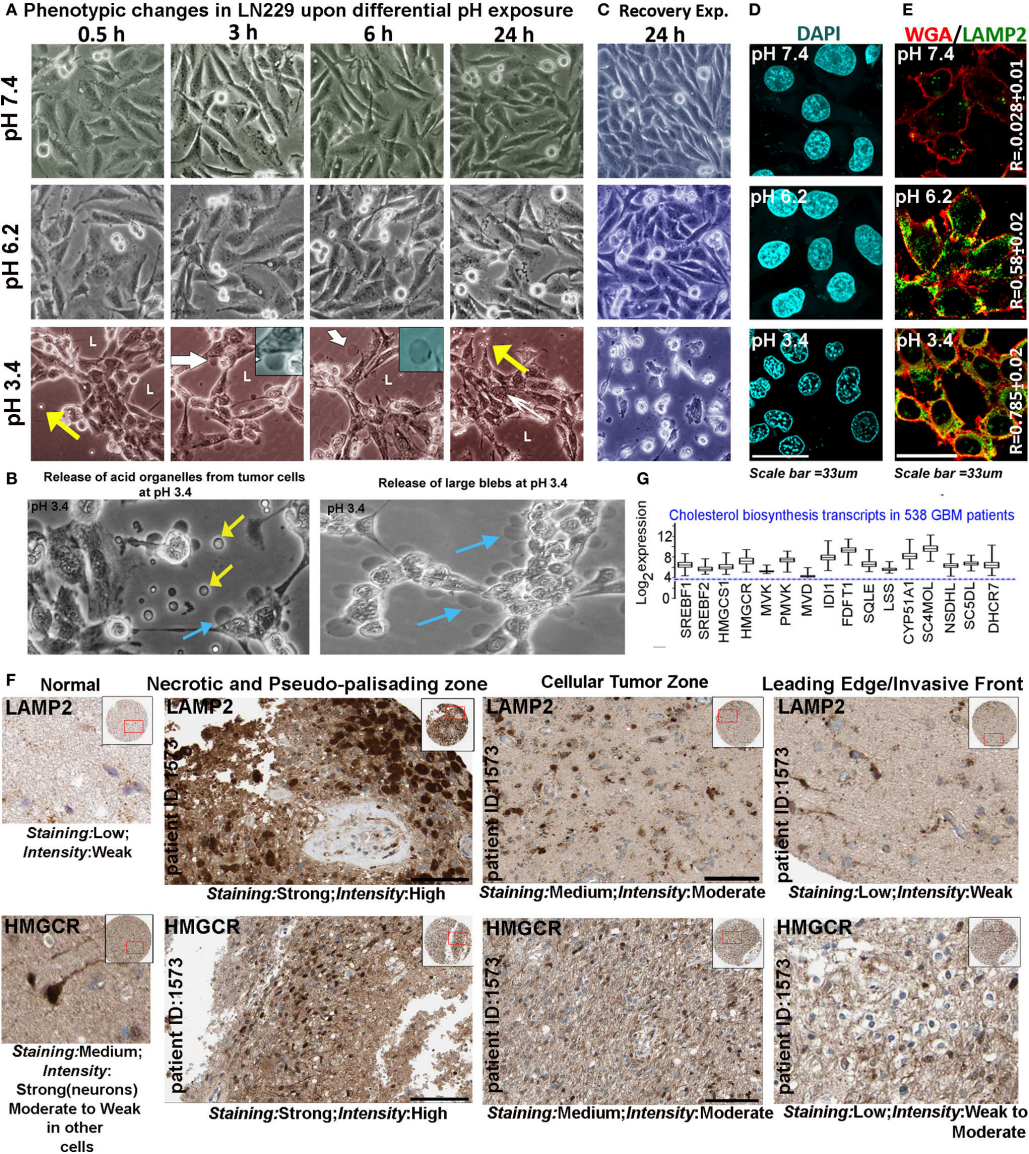
**Extracellular pH impacts glioblastoma tumor cell phenotype and suggests a possible association with cholesterol biosynthesis**. **(A)** LN229 glioblastoma tumor cell phenotype was monitored at pH 7.4 (physiological), pH 6.2 (low), and pH 3.4 (very low) for 24–72 h (data are shown for different time intervals in 24 h). Tumor cells at pH 7.4 and 6.2 showed spindle and rounded morphologies, while cells at pH 3.4 showed coagulation and release of small (yellow arrows) and large blebs (white arrows, see inset). Panel **(B)** shows zoomed images of small (yellow arrows) and large blebs (cyan arrows) formed at pH 3.4. **(C)** In recovery experiments, pH 3.4 exposed cells showed 100% de-adhesion and anoikis when reverted to physiological pH. **(D)** The nuclear morphology was not pyknotic in any pH range when monitored for 24 h, suggesting that no apoptosis was induced in low and very low pH ranges, in the time frame of the study. Additional supporting data for non-prominence of apoptosis, autophagy, and senescence at various pHs are described in Figures S2 and S3 in Supplementary Material. **(E)** LAMP2, a surface marker of progressive acidosis in breast cancer cells also showed significant surface localization in LN229 glioblastoma cells at low (pH 6.2) and very low pH (3.4) ranges, see colocalization coefficient (*R*) values between surface marker wheat germ agglutinin (WGA) and LAMP2. **(F)** HPA high-grade glioma patient data showed strong expression of LAMP2 (acidosis marker) and HMGCR (a rate-limiting enzyme in cholesterol biosynthesis) in necrotic/pseudo-palisading and cellular tumor zones. These zones are in general associated with very low and low pHs, respectively (see Figure 1A). The expression comparisons were made in the samples from the same patients. For comparisons in more patients, see Figure S4 in Supplementary Material. **(G)** TCGA glioblastoma patient microarray data too showed more than twofold increase in mRNA expression of major cholesterol synthesizing enzymes.

We chose pH of 7.4, 6.2, and 3.4 units as representatives of *physiological* (pH 7.4), *low* (pH 6.2), and *very low* (pH 3.4) proton concentration ranges for further studies on pH-associated oncogenic *vs*. non-oncogenic transformations. Briefly, by about 6 h, physiological pH range 7.4 showed well spread out and spindle-shaped morphologies (Figure 2A). Low pH range of 6.2 units showed both spindle-shaped cells and rounded phenotypes (associated with proliferative morphologies) (31, 32) and ‘very low’ pH range of 3.4 units showed progressive cellular aggregations, formation of pseudo-luminal areas indicated by L, and release of small vesicles-indicated by yellow arrows. Cells in pH range 3.4 also showed extensive liberation of micrometer-sized blebs that got detached from the cells and were seen floating freely into the medium, indicating buildup of high hydrostatic pressure at very low pH (cyan and yellow arrows in Figure 2B, white arrows and insets in Figure 2A) (33, 34). It is to be noted that in pH 3.4, the cells remained adhered for 3 days and beyond this, a slow peeling began to take place. However, when the tumor cells growing at low and very low pH were exposed to physiological pH medium, the cells in pH 3.4 showed 100% rapid anoikis, suggesting that very low pH-adapted cells cannot recover to physiological phenotype (Figure 2C). This observation supports the reports that buffer therapy may kill low pH-adapted cells (35). It is to be further noted that tumor cells adapted to pH 6.2, did not show any signs of anoikis in the recovery experiments. When such peeled/anoikis cells (pH 3.4 cultures re-exposed to pH 7.4 medium) were collected and re-plated in the physiological medium, they failed to show any residual growth or anastasis when followed for several days (36, 37).

Pyknotic nuclear morphology, an indicator of apoptotic cell death, was not observed in tumor cells adapted to different pHs (Figure 2D). Lack of ladder formation in DNA fragmentation assay also indicated that cells were not undergoing apoptosis even though cleaved caspase-3 levels were slightly high at pH 3.4 in comparison to other pH ranges (Figures S2A,D in Supplementary Material). Furthermore, cleaved PARP1, cleaved caspase-8, cell senescence, and autophagy markers also did not indicate prominent activation of any of these processes in different pH ranges (Figures S2B,C and S3A–C in Supplementary Material). Note that cleaved caspases were observed to be sequestered near the inner surface of the plasma membrane at low and very low pHs, a localization that cannot induce apoptosis (38).

Localization of LAMP2 to the plasma membrane is recently evidenced to protect breast cancer cells from acidosis and surface hydrolysis (18). It is a new histopathological marker for progressive tumor acidosis, and its expression levels are seen to be much higher in low pH tumor zones. We found a proportionate relocation of LAMP2 to the plasma membrane of LN229 acid-adapted cells (Figure 2E), wherein high surface localization was observed in tumor cells exposed to low and very low pH. Human Protein Atlas (39, 40) data also showed high expression of LAMP2 protein (Figure 2F) in necrotic and peri-necrotic pseudo-palisading zones (cells resident in very low pH). ‘Cellular Tumor’ zone, generally at low pH, showed moderate expression, while the ‘Leading Edge,’ generally at near physiological pH, had low levels. Interestingly, HMGCR, a rate-limiting enzyme in cholesterol biosynthesis, showed high corroboration with the expression trend lines of LAMP2 in different tumor zones, when compared to the same patient samples (Figure 2F, for more patient data, please see Figure S4 in Supplementary Material). Similar, correlation was observed for LAMP2 and SREBF2 (a transcription factor that regulates HMGCR mRNA synthesis), Figure S5 in Supplementary Material. These observations cumulatively suggested that low and very low pH zones may upregulate cholesterol for efficient acid adaptation both *in vitro* and *in vivo*.

As mentioned earlier, in physiologically pH-adapted cells, high levels of cholesterol and GM3 were detected on the surface (Figure S1 in Supplementary Material). This adaptation is proposed to prevent the cell membranes from acid hydrolysis, which is akin to mechanism suggested for LAMP2 function in the acid-adapted tumor cells. Hence, we first wanted to understand the role of cholesterol in tumor acid adaptation and cell fate transformations and then follow-up on GM3 glycosphingolipid.

**Figure 3:**
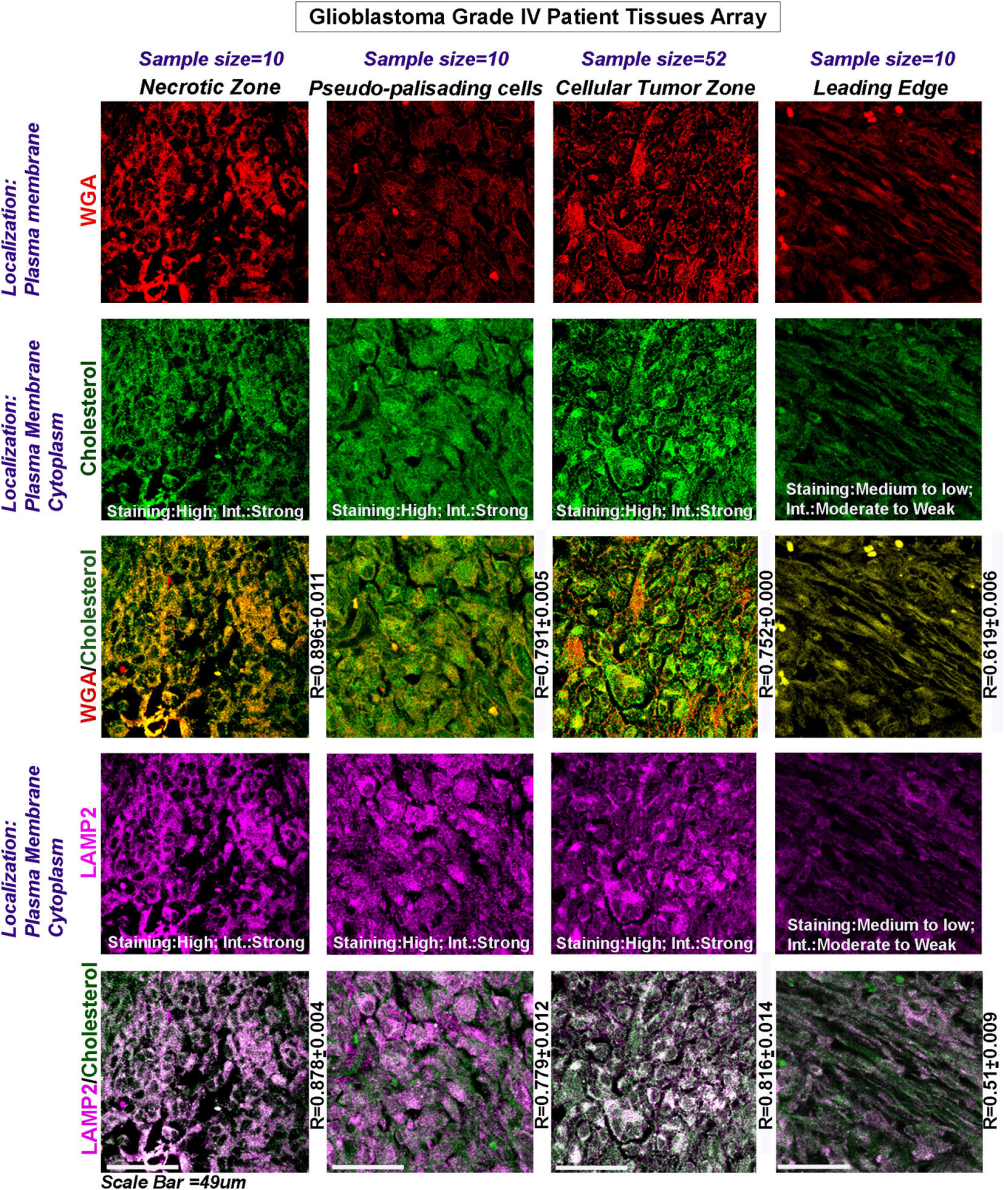
**Glioblastoma patient data show an increase in surface cholesterol and significant association with LAMP2, an acidosis marker protein**. Three glioblastoma tissue arrays bearing tissues from the same patients (63 distinct patient tissues) were processed as technical replicates. The tissues were probed for WGA (surface marker *WGA*: wheat germ agglutinin, Alexa 598 labeled, shown in red); cholesterol (green); and LAMP2 (an acidosis marker, shown in magenta): a change in surface residency of cholesterol in ‘different tumor zones’ was estimated quantitatively by colocalization coefficient values (*R*) between WGA and cholesterol staining (yellow regions). Qualitative significance was obtained by scoring the ‘Staining’ into high, medium, and low categories. ‘Intensity’ of expression was scored in terms of strong, moderate, and weak. The total number of ‘distinct GBM patient tissues’ considered for analysis of each tumor zone was indicated in the figure. Note that the average *R* values were derived from sample size for each tumor zone* three technical replicates. Note that there was a high surface localization of cholesterol in necrotic and pseudo-palisading zones, while cellular tumor zones showed moderate expression. LAMP2 colocalized well with cholesterol in these zones (white areas, magenta + green), suggesting that more acidic tumor zones have high surface cholesterol.

TCGA mRNA transcript data from 538 GBM patients showed high upregulation of cholesterol synthesizing genes (Figure 2G, see more than twofold increase in expression levels of HMGCR and SREBF2). Further, significantly high expression and colocalization of LAMP2 and surface cholesterol were detected in necrotic and pseudo-palisading zones of GBM patient samples, followed by moderate expression and colocalization in ‘Cellular Tumor’ zone *vs*. the leading edge areas (Figure 3).

This initial analysis, hence suggested that low and very low pH zones may transport more cholesterol to the surface for acid adaption and survival.

### Extracellular Protons Impact LN229 Glioblastoma Tumor Cell Physicochemical Dynamics in a Cholesterol-Sensitive Manner

**Figure 4:**
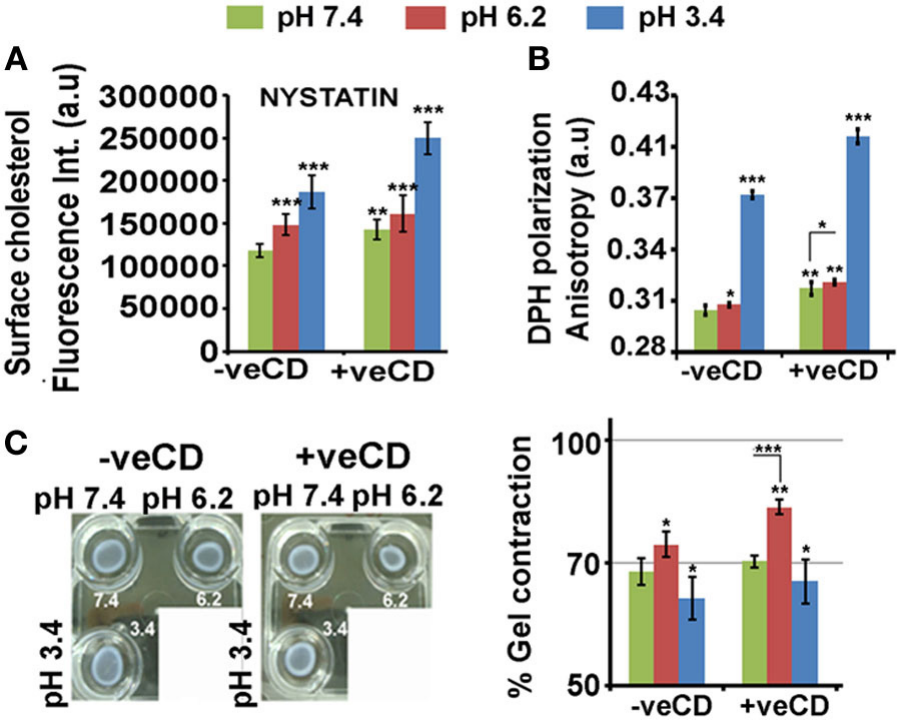
**Extracellular pH impacts LN229 glioblastoma tumor cell surface cholesterol levels and associated physical properties**. Nystatin dye binds to cholesterol and hence enables its surface detection and quantitation. **(A)** Image-based quantitation of cholesterol *via* nystatin dye signal was performed in LN229 glioblastoma tumor cells. Data show an increase in surface cholesterol with decreasing pH. **(B)** Surface rigidity or anisotropy was measured through DPH dye polarization. A decrease in pH led to increase in surface rigidity. **(C)** Gel contraction assay showed that tumor cells with high surface cholesterol contracted more and were far competent in dissipating mechanical load. Error bar = SD; no. of independent experimental replicates = 3; *p ≤ 0.05, **p ≤ 0.01, ***p ≤ 0.001.

As discussed in the earlier section, there may exist a cross talk between the low pH interfacing cell surfaces and high surface cholesterol to shield tumor cells from acidosis. A significant rise in cholesterol was also observed when LN229 glioblastoma cell line was exposed to lower pH ranges of 6.2 and 3.4 units (Figure 4A; −veCD). The plasma membrane cholesterol quantitation was performed *via* imaging of signal from cholesterol binding nystatin dye that was incubated on the surface of (i) live cells (kept on ice, to prevent endocytosis; cells were fixed after staining and imaged) and (ii) also post-fixation (Figure S6B in Supplementary Material). Similar results were obtained when measurements were made through fluorescence spectroscopy (Figure S6C in Supplementary Material).

When the glioblastoma cells in different pH microenvironments were further perturbed with a very low concentration of methyl-beta-cyclodextrin (+veCD 10 µM) (37), a significant rise in cholesterol from its initial values was observed at all pH ranges (Figure 4A). This may be a feedback response to the initial loss in cholesterol by CD treatment, highlighting the need to maintain cholesterol levels in pH-graded tumor microenvironments. Hence, by this strategy, we were able to generate tumor cells with enhanced levels of cholesterol. This enabled us to study the effects of much higher levels of cholesterol on cell fate competencies in response to differential proton concentrations.

Cholesterol levels crucially determine surface rigidity/anisotropy (41, 42). Surface rigidity is known to determine tumor cell fates (43). The low (6.2) and very low pH (3.4) microenvironments in the unperturbed cholesterol condition (−veCD) showed enhanced surface anisotropy or rigidity, and this was further elevated in the high cholesterol conditions (+veCD) (Figure 4B).

Higher cholesterol levels increase the substrate adhesion energies for efficient mechanical load dissipation, and the gel contraction assay suggests that indeed high cholesterol pH 6.2 +veCD gels contracted more and hence were far competent in the dissipation of the mechanical force caused due to ECM–tumor cell interactions (Figure 4C) (44). Of note was that gels in pH 3.4 microenvironments also contracted moderately, again suggesting that the cells in this pH condition were not dead but were under high membrane tension and could manage to moderately release tension by contracting. Further, gels devoid of cells, failed to contract in pH adjusted medium, confirming that contractility was produced by cells and not by the medium.

Surface rigidities and mechanical homeostasis directly determine tumor metabolic milieu (45, 46). The surface mechanical states are communicated to the chromatin *via* nuclear translocation of several surface resident mechanosensitive transcription factors that then enable appropriate adaptive modulations in cellular metabolic states. The cortical actin also relays the information on the surface stiffness to the chromatin through stress fibers that are focally linked to the nuclear envelope. This further enables adaptive modulations in metabolic gene expressions (47). Besides, the surface stiffness also induces conformational changes in several metabolic enzymes to prevent their degradation. Surface rigidities crucially determines the trafficking of channels that maintain the pHi of the tumor cells to effect the functions of energy metabolism-associated proteins and enzymes, even in low extracellular pH conditions (48–51).

**Figure 5:**
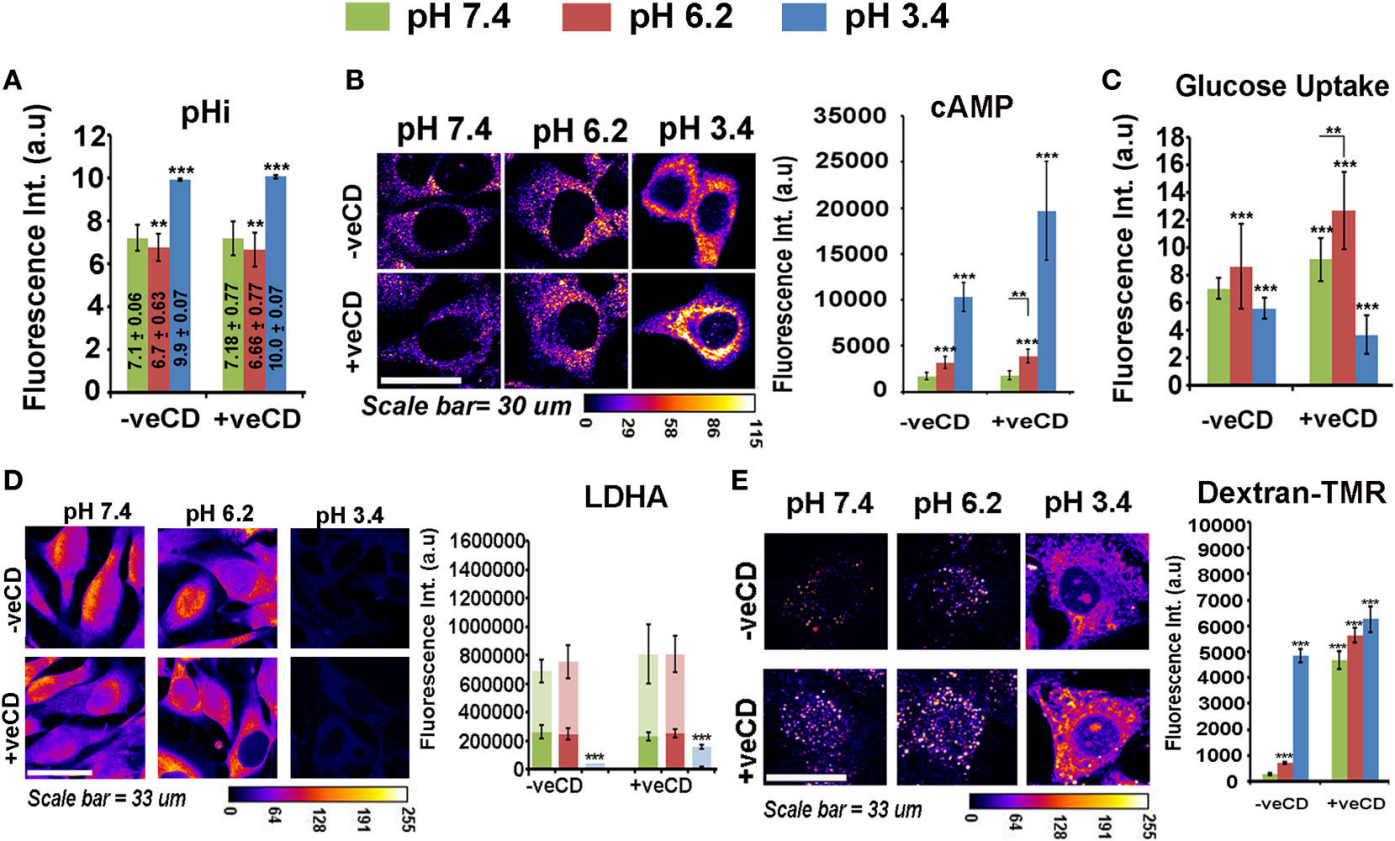
**Extracellular pH impacts LN229 glioblastoma tumor cell biochemical homeostasis in a cholesterol-sensitive manner**. Glioblastoma cells with basal and high surface cholesterol levels were incubated in various pH ranges. **(A)** Intracellular pH (pHi) in different extracellular pH (pHe) conditions was measured in live cells by using pH Rodo-based fluorescent microscopic imaging. **(B)** Cellular cAMP levels were measured by immunostaining. **(C)** Glucose uptake was measured by 2-NBDG assay. **(D)** LDHA, a crucial enzyme in anaerobic glycolysis, was measured by immunostaining and **(E)** Macropinocytosis was probed in live cells *via* dextran–TMR uptake assay. These assays enabled us to understand the overall cell energetics in different pH ranges. Error bar = SD; no. of independent experimental replicates = 3; *p ≤ 0.05, **p ≤ 0.01, ***p ≤ 0.001.

We find that low and very low pH microenvironments in −veCD and +veCD conditions showed higher intracellular alkalinity (Figure 5A) in comparison their respective external pH environments. Since most of the glycolysis-associated enzymes become inactive at very low pHi; this rise in intracellular pH was particularly important adaptation for tumor survival in low pH conditions. Surprisingly, cAMP, a known soluble mediator of bicarbonate channels, also showed high levels in ‘very low’ pH environment; hence, these tumor niches were rather quite successful in maintaining very high pHi and cAMP pools for their survival (Figure 5B) (52, 53). We find that pH 6.2 had high glucose uptake which was significantly increased in higher cholesterol conditions at both pH 7.4 and 6.2 (Figure 5C). Cells at very low pH (3.4 units) had reduced glucose uptake and negligible levels of LDHA, a crucial enzyme in anaerobic glycolysis (Figure 5D). Although the observation on the absence of anaerobic glycolysis-associated marker fits well with increased intracellular alkalinity at ‘very low’ pH, we wondered how tumor cells at pH 3.4 were able to generate high levels of ATP to sustain high levels of cAMP, if LDHA levels were low.

It came to our notice that macropinocytosis is an alternative mechanism by which cells drive excess nutrients from the extracellular microenvironment (54–56), and we observed that this phenomenon was enhanced at both ‘low’ and ‘very low’ pH microenvironments in −veCD conditions and at all pH ranges in +veCD-treated cells (Figure 5E) (as measured by the uptake of dextran–TMR, an assay to detect macropinosomes). Hence, high cholesterol and low pH microenvironments used multiple and heterogeneous pathways for active metabolism in comparison to tumor cells resident in pH 7.4 −veCD (basal cholesterol) condition.

### Extracellular Protons Impacts LN229 Glioblastoma Tumor Cell Fate Dynamics in a Cholesterol-Sensitive Manner

The physicochemical and metabolic remodeling *via* differential proton concentration may act as a stimulus for generation of heterogeneous tumor cell fate, a possibility that was further explored.

**Figure 6:**
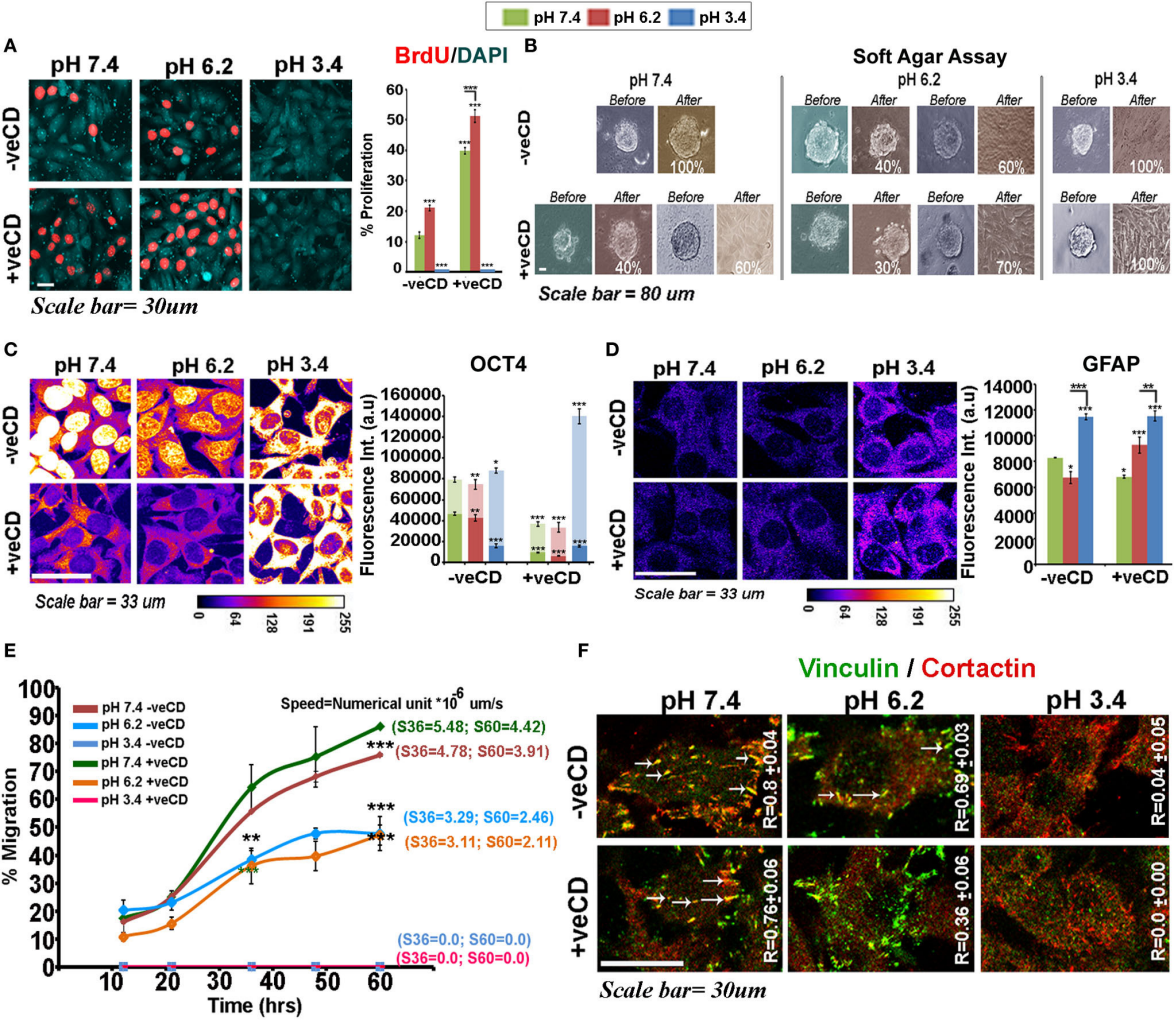
**Extracellular pH impacts LN229 glioblastoma tumor cell proliferation *vs*. differentiation dynamics in a cholesterol-sensitive manner**. **(A)** LN229 cells with basal and high surface cholesterol levels were subjected to different pH microenvironments and were then tested for the proliferation status *via* BrdU labeling. **(B)** Soft agar anchorage-independent growth assay was performed to examine the impact of various pH ranges on tumor cell self-renewal property. For this, live cell imaging of about 15–20 glioma spheres in each condition was followed for 3 days. **(C)** Immunostaining with OCT4, an often used marker of substrate-independent growth was performed and its nuclear *vs*. cytoplasmic localization at different pHs was compared in tumor cells. The *in vitro* observations on Oct4 immunostained patterns were also found to corroborate well with the patient data (please see Figure S7 in Supplementary Material). **(D)** GFAP, an astrocytic differentiation marker, showed significantly high levels in very low pH conditions. This observation was concordant with *in vivo* GFAP staining in necrotic and pseudo-palisading zones of glioblastoma multiforme tissues (Figure S8 in Supplementary Material). **(E)** The wound healing-based migration assay showed differential metastatic competence with varying pH microenvironments where very low pH condition promoted motility arrest. Tumor cells at physiological pH were most migration competent in both basal and high surface cholesterol conditions. Note that letter ‘S’ denotes the speed of migration of tumor cells and values of speed at 36th and 60th hours is particularly highlighted. **(F)** Colocalization of cortactin and vinculin at migratory focal adhesion structures (highlighted with white arrows) in cells exposed to various pH is indicated by Pearson’s coefficient (*R*-value). Error bar = SD; no. of independent experimental replicates = 3; *p ≤ 0.05, **p ≤ 0.01, ***p ≤ 0.001.

The BrdU-labeling index showed that high cholesterol tumor cells were proliferation competent and the high cholesterol pH 6.2 condition was most proliferative (Figure 6A). However, to further examine whether different pH ranges also impact substrate anchorage-independent proliferation and growth (that gradually evolves stem-like cells), we ran the soft agar anchorage-independent growth assay (Figure 6B). Gliomaspheres incubated in the unperturbed cholesterol physiological pH condition were 100% anchorage independent and lower pH treatments flattened the spheres, which de-promoted long-term anchorage-independent growth. The −veCD pH 6.2 condition showed approximately 60% sphere flattening, probably due to an increase in cholesterol-mediated surface rigidity. The +veCD conditions showed heterogeneous behavior with 60–70% flattening of spheres at pH of 7.4 and 6.2 units, respectively. Notably, spheres at the ‘very low’ pH in all conditions showed flattened morphology without any further growth when monitored for three consecutive days. Hence, high cholesterol containing tumor cells, though more proliferative in 2D cultures, were diminished of substrate-independent growth properties *vs*. the tumor cells growing in the unperturbed cholesterol physiological pH condition (7.4 −veCD).

We further found that Oct4 (Figure 6C), an often used marker for substrate-independent growth, was diminished nuclearly in pH 6.2 −veCD condition and its total levels also dropped in the high cholesterol physiological and pH 6.2 conditions. Oct4 showed reduced cytoplasmic accumulation in +veCD pH 7.4 and 6.2 conditions but high accumulation in the cytoplasm at very low pH in all conditions, a phenotype also observed in the gastric cancers (57, 58) and glioblastoma biopsies (see expression in different tumor zones of GBM patient samples—Figure S7 in Supplementary Material). Reduction in nuclear Oct4 in high cholesterol conditions may be one of the major reasons for diminished anchorage-independent growth. Hence, Oct4 nuclear *vs*. non-nuclear localization in the response to pH and cholesterol levels may crucially determine differential cell fates. In fact, the cells at very low pH range showed very high upregulation of glial acid fibrillary protein, GFAP, a well-known marker of astrocyte differentiation (Figure 6D; see expression in different tumor zones of GBM patient samples—Figure S8 in Supplementary Material). Also, the distribution and concentration of GFAP protein at this pH were membranous and sub-membranous, while it was predominantly cytoplasmic in the other pH ranges. This suggests that ‘very low pH’ can program tumor cells toward differentiated and non-oncogenic fate.

While high cholesterol pH environment of 6.2 and 7.4 units excelled in proliferation and pH 3.4 in differentiation-like status, the wound healing assay showed a different ranking in migration competence of pH-graded microenvironments, with very low pH conditions being highly migration incompetent (Figure 6E). Tumor cells at physiological pH were most migration competent closely followed by cells growing in high cholesterol physiological pH microenvironments. However, tumor cells at pH 6.2 units migrated more slowly than physiological pH exposed cells (Figure 6E).

The migration competence showed high coroborration with enhanced colocalization of cortactin and vinculin at the focal adhesion structures in pH 7.4, as indicated by Pearson’s coefficient (*R*) (Figure 6F, white arrows, required for efficient migration). High *R* values indicated that the pH 7.4 microenvironment was intrinsically highly ‘metastasis competent.’ These observations are much supported by the evidence that tumor margins, essentially at near physiological pH are most migration competent (59, 60). Similar corroboration was derived from the other components of the focal adhesion such as the α-actinin–F-actin association, the levels of surface integrins, and its colocalization with the underlying alpha-tubulins and juxtapositioning of Rac1 to the inner leaflet of the plasma membrane, all of which generate an efficient migration competence (Figure S9 in Supplementary Material).

### Cholesterol Depletion Impacts Survival of Low pH-Adapted LN229 Glioblastoma Tumor Cells

**Figure 7:**
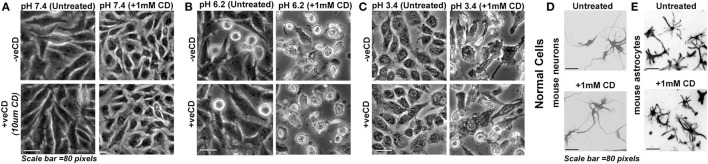
**Depletion of surface cholesterol causes anoikis in low pH-adapted LN229 glioblastoma tumor cells**. **(A–C)** LN229 glioblastoma cells with basal (−veCD) and high surface cholesterol level (+veCD) were exposed to different pH ranges and were then treated along with 1 mM CD (methyl-beta-cyclodextrin that removes cholesterol from the surface at mild to high concentrations). Normal cells: **(D)** mouse cerebellar granule neurons and **(E)** mouse cortical astrocytes were also treated with 1 mM CD. Within 48 h, 100% anoikis was observed only in glioblastoma cells at low and very low pH ranges. There was no visible anoikis in normal cells. Interestingly, glioblastoma cells growing at physiological pH were completely protected from this treatment. This suggests that removal of surface cholesterol makes low pH-adapted cells highly vulnerable to acid-mediated cytotoxicity. Note that mouse neurons and astrocytes were fixed after the experiment and labeled with Tuj1 and GFAP antibodies, respectively, to reveal morphologies clearly. The immunostained images are shown in grayscale. The experiment was repeated three times.

Knowing that cholesterol levels in different pH microenvironments can crucially impact cell fates and growth, we decided to deplete the surface cholesterol from acid-adapted and non-acid-adapted tumor cells by treatment with 1 mM methyl-beta-cyclodextrin (MBCD/CD). Unlike 10 µM CD that enhances surface cholesterol levels by a feedback mechanism, 1 mM and above concentrations of CD are shown to sequester cholesterol from surface permanently, in a dose-dependent manner (61). However, 1 mM MBCD/CD treatment is shown to be mild in normal cells (also tested by us, Figures 7D,E). We find (Figures 7A–C) that tumor cells resident in low pH (6.2) and very low pH (3.4) microenvironments began to peel off within 30 min of treatment and by about 1 h all the cells in pH 3.4 microenvironment had peeled off and showed necrosis. By about 3 h, all the cells in pH 6.2 microenvironment showed the same effect while cells growing in physiological pH were unharmed. We collected the floating necrotic cells and upon centrifugation, pelleting and resuspension in the physiological medium, we re-plated the suspension in plastic dishes and on the soft agar. We did not find any evidence of residual cell revival/anastasis or recovery upon monitoring the cultures for next 7 days. Hence, the depletion of surface cholesterol in acid-adapted cells removed the protective shield and made cells vulnerable to acid-mediated toxicity and permanently killed them by anoikis. The very low pH (3.4)-adapted cells showed more rapid vulnerability to this treatment *vs*. the low pH (6.2)-adapted tumor cells.

### Extracellular Proton Concentrations May Induce GM3 Glycosphingolipid Conformational Heterogeneity *via* the Glycan Moiety

Since physiological pH-adapted tumor cells were unharmed by moderate cholesterol depletion (Figure 7A), we questioned whether GM3 glycosphingolipid, which is also seen to be high in acid-adapted cells, can be exploited to eradicate tumor cells in all pH microenvironments, including cells growing at the physiological pH.

**Figure 8:**
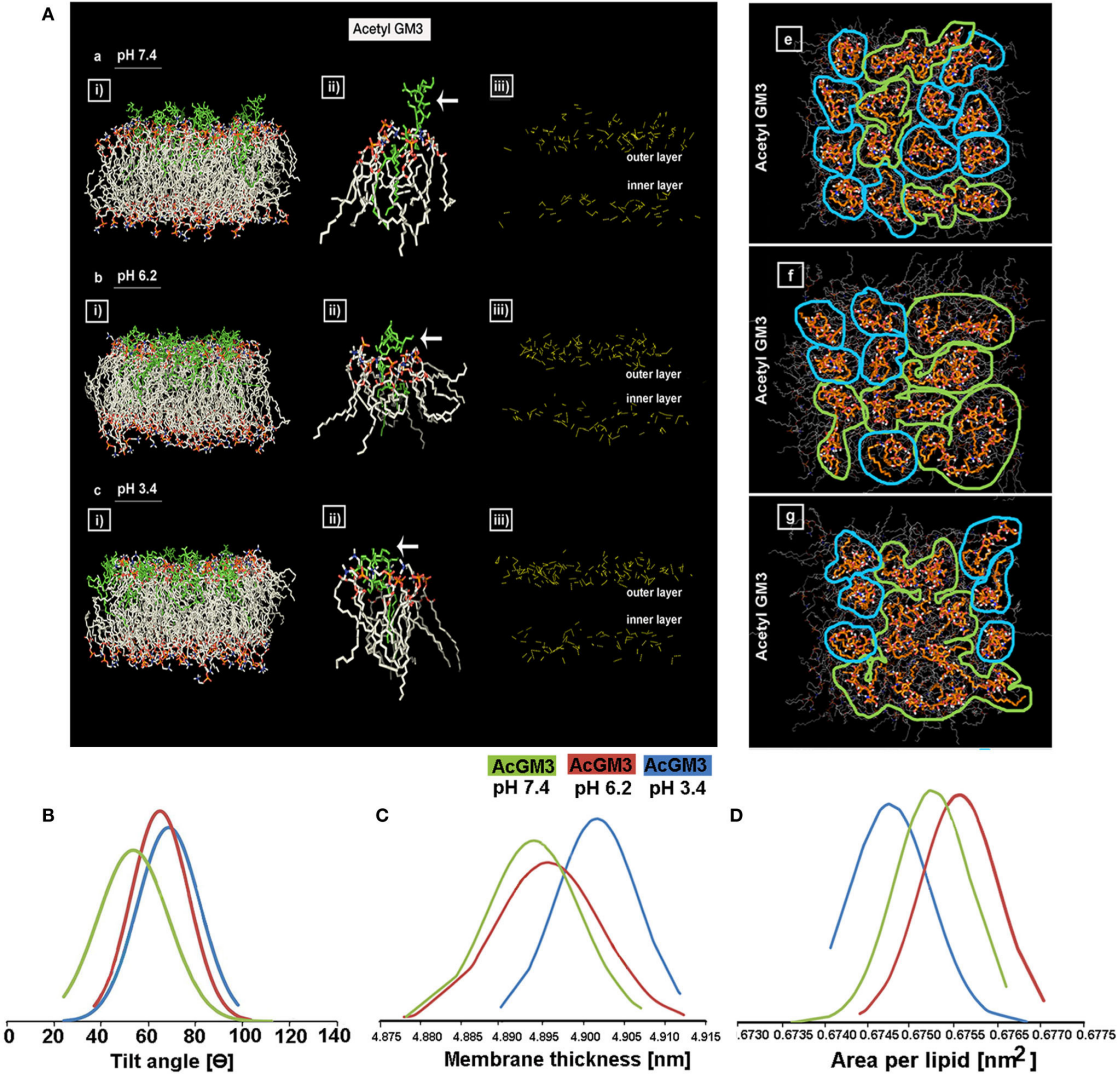
**GM3 demonstrates conformational heterogeneity in response to pH ranges**. Panel **(A)** a–c (i) shows *x*–*z* projection of AcGM3 in GM3–POPC asymmetric bilayer. Panel **(A)** a–c (ii) shows single AcGM3 molecule average conformation in lipid bilayer at pH 7.4, 6.2, and 3.4, respectively. Panel **(A)** a–c (iii) shows outer leaflet-associated density and compactness of H-bonds (yellow dotted lines) of AcGM3 in vertical projections at pH 7.4, 6.2, and 3.4, respectively. Panel **(A)** e–g is the *x*–*y* projection of the simulations and shows GM3 monomers at pH 7.4, dimers at pH 6.2, and lipid clusters at pH 3.4. Panel **(B)** shows preferred tilt angle of GM3 glycan headgroup at various pH ranges. Panels **(C,D)** represent the effects of GM3 conformational heterogeneity (at different pH) on membrane thickness and lipid packing, respectively.

We began by running atomic level molecular dynamic simulations of GM3–POPC in an asymmetric bilayer, to understand whether GM3 can respond to microenvironmental pH levels and thereby can act as a pH sensor and cell fate regulator. The density of hydrogen (H) bonds [Figure 8A: a–c (iii) shown as yellow dotted lines] in the outer leaflet containing GM3 was found to be progressively increased with a decrease in pH (from *physiological*: pH 7.4, *low pH*: pH 6.2, to *very low*: pH 3.4). The H-bonds at pH 6.2 and 3.4 showed reduced vertical projection [Figure 8A: a–c (iii)] probably due to compactness of H-bonds. GM3 at the physiological pH, in contrast, basically had more vertical projections from the surface. This explanation was supported by the observations that glycan headgroup mainly stuck out of the plane of the bilayer at the physiological pH [Figure 8A: a (ii), see white arrow] which gradually tilted toward the bilayer with the decreasing pH values [Figure 8A: b,c (ii) indicated by white arrows]. The *x*–*y* projection of the simulations showed that due to increased H-bonding between glycans of GM3, there was an evolution of dimers at pH 6.2 and more clustered organization at pH 3.4 (Figure 8A: e *vs*. f,g). A detailed analysis of the H-bonds between different glycans of the neighboring as well as self in GM3 revealed that GM3 H-bonds were predominantly established by the sialic acid [*N*-acetylneuraminic acid (NANA)] moieties at pH 6.2 and 3.4, followed by intermolecular interaction *via* galactose (Gal) (see Table 1). However, at pH 7.4, the H-bonds were formed predominantly with water and the total numbers of intermolecular H-bonds per nanosecond were particularly low.

**Table 1:** **pHe gradient induced H-bond interactions in GM3–POPC bilayer and role of sialic acid**.

		AcGM3	
Intermolecular H-bonding partners	pH 3.4	pH 6.2	pH 7.4
NANA-NANA	**0.19**	**0.17**	0.04
Gal-Gal	**0.17**	–	0.02
Glc-Glc	0.01	–	0.04
Gal-NANA	0.07	0.05	0.02
Glc-NANA	0.06	0.08	0.08
SP-NANA	–	0.02	–
SA-NANA	–	0.02	–
NANA-POPC	0.15	0.16	0.09
Gal-POPC	0.05	0.07	0.09
Glc-POPC	0.11	0.18	0.20
SP-POPC	0.04	0.06	0.05
SA-POPC	0.01	0.01	0.02
Charge pairs	0.07	0.09	0.08
H-bonds with H_2_O	0.05	0.10	**0.28**

*Average number (expressed in %) of intermolecular H-bonds and charge pairs between GM3–GM3, GM3–POPC, and GM3–water in systems at various pH. H-bonds formed per nanosecond were analyzed for last 100 ns in a 200-ns simulation. Each system consisted of 16 GM3, 143 POPC, and around 10,966 water molecules (error < 0.02). The expansion of terms used in table are as follows: NANA, sialic acid moiety of GM3 (*N*-acetylneuraminic acid of acetyl GM3); Gal, galactose moiety of GM3; Glc, glucose moiety of GM3; SP, sphingosine lipid tail of GM3; SA, stearic acid lipid tail of GM3; POPC, 1-palmitoyl-2-oleoyl-phosphatidylcholine; charged pair species, dipole pairs within the molecule*.

*Bold font indicates the most significant values in the phenomenon studied*.

The results obtained by us in bi-component membrane simulations at pH 7.4 are much in concert with the recent report on MSD data of GM3 clusters on complex lipid membranes (62). However, since our focus was also on the GM3 conformational patterns at the lower pH units, our MSD results suggests that acidic pHs can trigger a lipid shielding effect of Gal and NANA sugar moieties toward the POPC bilayer.

To further understand the basis of competence of different pHs in evolving GM3 receptor heterogeneity by local spatial changes in its glycan headgroups, we studied the tilt angles (Figure 8B; Figure S10 in Supplementary Material). While preferred tilt angle of glycans at pH 7.4 peaked at 50°, it peaked to 62° and 69° in pH 6.2 and 3.4, respectively. This suggests that at low pH, sialic acid can tilt more to find its surrounding binding partners. A look at the net effects of this pH-driven GM3 glycan conformational heterogeneity on its acyl chain orientation, that guides the membrane thickness, suggests that the membranes were progressively thicker at the lower pH units, indicative of stretched acyl chains in the membrane (Figure 8C).

We further investigated the compression of lipids per unit area in response to the decreasing pH. Results show that ‘area per lipid’ was reduced at low and very low pHs, suggesting an evolution of high lipid packing density. Hence, enhanced lipid packing at low pH and very low pH indicated the probability of high compressional stress on the membrane (Figure 8D).

### GM3 Glycosphingolipid Show *In Vivo* and *In Vitro* Differential Surface Clustering in Glioblastomas

**Figure 9:**
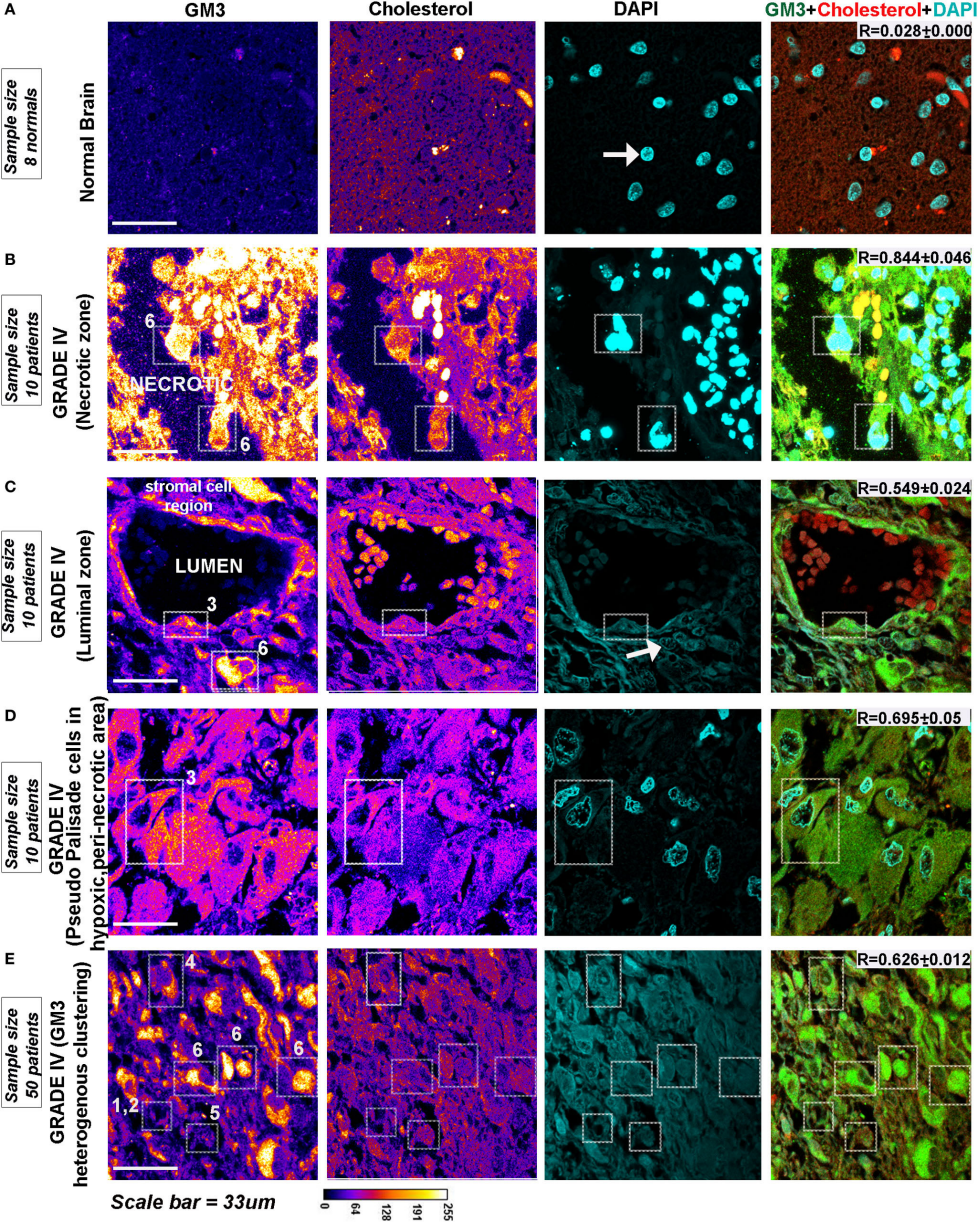
**Glioblastoma patient tissues reveal GM3 heterogeneous clusters with prominent supraclustering in prospective low pH zones**. **(A–E)** Immunohistochemistry on (human) normal brain samples and glioblastoma grade IV patient tumor tissues was performed to probe the levels and organization of GM3 glycosphingolipid and its colocalization with cholesterol. **(B–D)** GM3 was enriched in necrotic zones, luminal, and peri-necrotic zones where it showed large punctate/clustered pattern on the cellular surface. **(E)** Non-luminal zones, cellular tumor zones showed differential patterns where either small puncta of GM3 were observed all over the cells, or GM3 polarized clustered assemblies were noticed, suggestive of a migratory phenotype. The white boxes in the panels and numbers alongside each box indicates distinct pattern of GM3 cluster organization. Note that the figure bears a reference number for each pattern which is described as follows—No. 1 is used for very few and small GM3 clusters; No. 2 is used for more but small clusters; No. 3 is used for large clusters dispersed all over the cell; No. 4 is used for numerous but small and polarized clusters; No. 5 is used for small clusters with few large clusters dispersed all over the cell; and No. 6 is used for very large clusters covering the entire surface of the cell. Calibration bar for LUT converted images is shown alongside the image. Each glioblastoma multiforme patient tissue array consisted of 63 patient samples. Three samples showed medium GM3 staining while rest showed high GM3 levels on a visual scoring scheme of High, Medium and Low expression. *R* values indicate the extent of colocalization between GM3 and cholesterol in different zones of glioblastoma.

GM3 is highly expressed in gliomas and is shown to be a crucial component of the migratory complex (63, 64). Immunohistochemistry from glioma patient revealed that GM3 is highly expressed in malignant glioblastomas and was well colocalized with cholesterol-rich regions in the WHO grade IV (Figure 9) cancers in both pediatric and adult patients *vs*. grade II (Figure S11 in Supplementary Material, see table embedded in the figure for comparative *R* values). GM3 was highly enriched in the necrotic and luminal zones where it showed large punctate/clustered patterns on the cellular surface (phenotypes designated by numbers 6 and 3) (Figures 9B,C, white arrow points to very low pH-associated nuclear morphology as observed in Figure 2D). However, the non-luminal zones showed differential patterns where either a mix of large and small puncta were observed all over the cells (a phenotype designated by number 5) or small punctate and polarized clustered assemblies were noticed, suggestive of a polarized migratory phenotype (a phenotype designated by number 4) (Figures 9D,E). Further, small phase separated clusters were observed in some tumor cells (phenotypes designated by numbers 1 and 2). All these patterns of GM3 were well correlated with the expression of cholesterol in the tumor cells and also with GM3 and cholesterol co-expression patterns in the human kidney and the gastric tissues which are the naturally acid-adapted organs (Figure S1 in Supplementary Material). Hence, it appears that in a very low acidic environment, the tumor pH induces conformational changes in GM3 and simultaneously upregulates cholesterol to induce a lipid shielding effect against harsh acidic environment, which is generated by the extrusions of proton from the nutritionally starved and over-active tumor cells.

**Figure 10:**
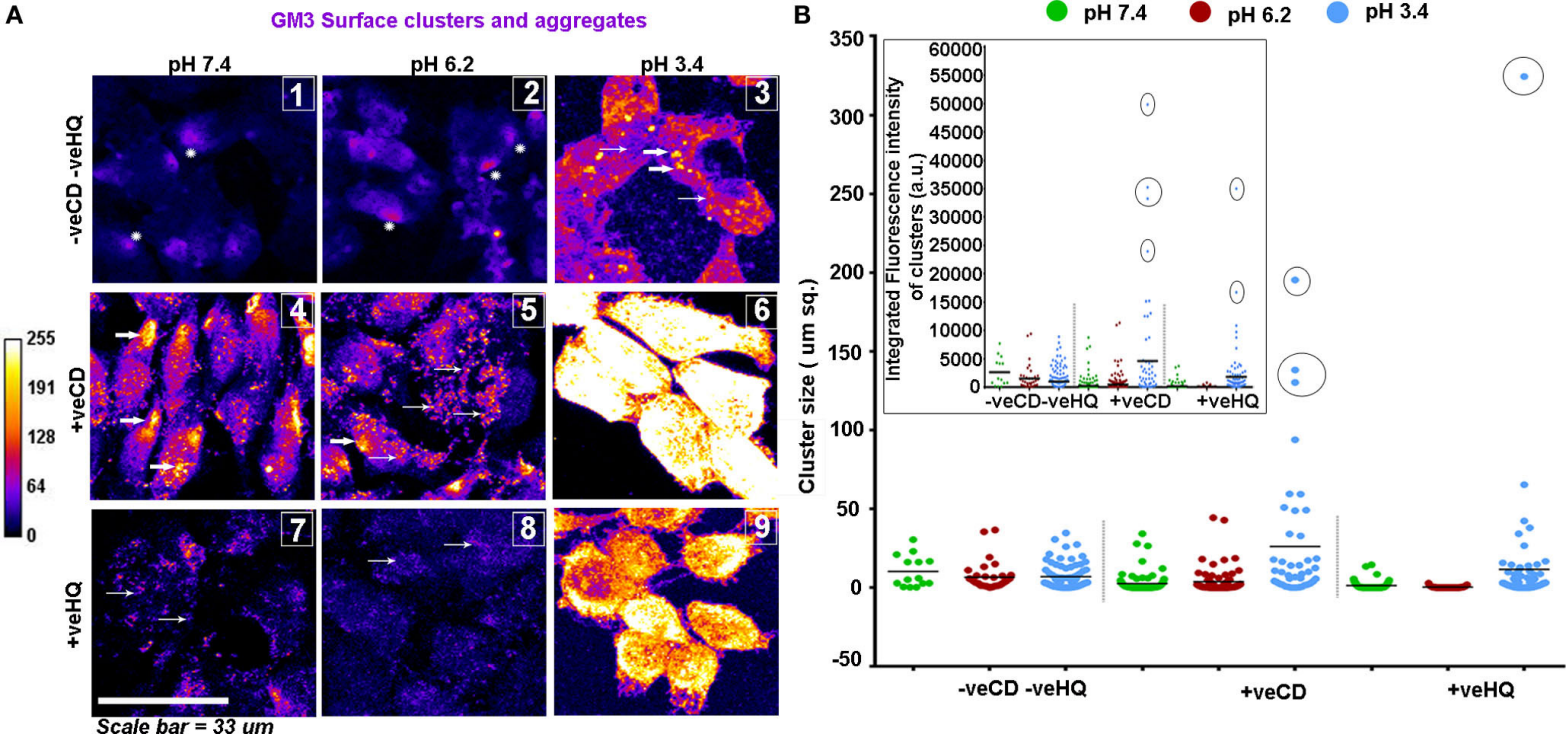
**LN229 glioblastoma tumor cell line shows variable GM3 surface clustering at different pHe**. **(A)** Tumor cells at various pH and with different surface cholesterol levels (−veCD and +veCD conditions) were incubated with the anti-GM3 antibody on ice for 45 min; cells were fixed and developed with a fluorescent secondary antibody to visualize GM3 organization on the plasma membrane. Also, to study the dependency of GM3 clusters on underlying actin, cells in different pH microenvironments in −veCD condition were treated with 50μM HQ to disrupt cytoskeleton. Images were captured with a confocal microscope and differential clustering patterns (size and intensity) were analyzed using Fiji software. Each image in panel **(A)** bears a number as an inset to describe the clustered pattern which matched with the patterns observed in glioblastoma multiforme patient tissues shown in Figure 9. No. 1 indicates small phase separated domains (shown with white stars) that are overall few in numbers; No. 2 indicates small phase separated domains (shown with white stars) that are overall more numerous than in pH 7.4 −veCD−veHQ condition. Thick white arrows in the image labeled as No. 3 indicates large punctate clusters; Thick white arrows in the images labeled as No. 4 and No. 5 indicates dense polarized clusters, while thin arrows indicate smaller clusters. No. 5 image shows few large clusters too. No. 6 image shows very large and a few small clusters covering the entire surface of the cell. Thin arrows in No. 7 and No. 8 images indicate very small, diffuse, and less dense GM3 clustering. **(B)** Cluster size/area/cellular surface spread was analyzed in Fiji image processing software, and data were plotted using GraphPad Prism software, density of cluster was quantified *via* fluorescent intensity measurements. Error bar = SD; no. of independent experimental replicates = 3; *p ≤ 0.05, **p ≤ 0.01, ***p ≤ 0.001.

We were rather excited to see that the patterns obtained on the GBM patient tumor tissue sections strongly corroborated with the GM3 surface organization in our pH range *in vitro* studies (Figures 10A,B).

Overall, the unperturbed cholesterol conditions (−veCD) at physiological and low pH had reduced size and number of clusters, in comparison to the high cholesterol pH conditions, but pH 6.2 (−veCD) showed more number of clusters than pH 7.4 (−veCD), which was also predicted by our simulation data. Both GM3 cluster size and cluster fluorescence intensity that essentially denoted the number of expressing pixels in a cluster were found to be highly correlated in each condition (Figure 8B).

The high cholesterol tumor cells (+veCD) at physiological pH 7.4 showed numerous small sized GM3 clusters (0.17 μm^2^) that were organized in a polarized pattern. However, the pH 6.2 exposed cells had comparatively fewer punctate clusters in the range of 0.17 μm^2^ but rather showed some larger sized clusters (0.34 μm^2^ and above), probably due to the high levels of cholesterol in these conditions. The few but larger clusters observed in pH 6.2 +veCD condition could have exerted a high compressional force on the tumor cells, forcing a spherical mitotic geometry, which might be the reason for the observed highest proliferative index of this condition (Figure 6A). The very low pH 3.4 exposed cells showed an evolution of very large sized clusters that almost appeared to cover the entire cell surface. Such very large numbers and sizes of GM3 clusters can exert very high compressional stress on tumor cells (as predicted in MSD data) which could have been responsible for the growth arrested phenotype at this pH.

The GM3 cluster size and intensity were highly diminished at all pH conditions from their original values in HQ (cytoskeleton disrupter) treated cells, suggesting that the major micron-sized GM3 clustering was cytoskeleton dependent. Since +veCD conditions also showed enhanced clustering, it can be assumed that micron-sized clusters were cholesterol and cytoskeleton dependent.

We confirmed that the clustering patterns obtained were not due to the cluster detection assay performed at a low temperature because in experiments where the cells that were first fixed at the room temperature and then probed for surface GM3 also showed similar surface patterns as seen when the GM3 antibody was incubated on the surface of live cells that were kept on ice, to prevent antibody endocytosis (Figure S12A in Supplementary Material). When live cells in different conditions were treated with saponin to deplete the surface cholesterol and then probed for surface GM3, the GM3 clustering pattern was highly reduced in all conditions at all pH units, again suggesting that clustering was sensitive to the levels of cholesterol (Figure S12B in Supplementary Material). We find that pH and cholesterol-driven GM3 differential clustering was specific and its precursor lipid species, lactosylceramide or another ganglioside SSEA4 (65) did not exhibit this type of clustering phenomena when exposed to the similar conditions (Figures S12C,D in Supplementary Material).

We further considered that the cholesterol-independent GM3–self-glycan interactions, as seen in lactonized forms, may also exist in low pH conditions, at least to some extent, and may contribute to observed GM3 clustering (17, 66). For this, we investigated the extent of contribution of low pH-induced GM3 lactones (intra-glycan bonding between sialic acid and other sugar moieties) *vs*. GM3–GM3 glycan ligated clusters in our pH conditions. Data showed that GM3 lactonization was rather minimal as the digestion of the sialic acid by sialidases, dramatically reduced surface GM3 detection at all pHs, which would not have been the case if detected clusters were predominantly lactones, as GM3 lactones are resistant to such enzymatic treatment (Figure S13 in Supplementary Material) (67).

### GM3 Clustering Antibody Can Partially Mimic the Very Low pH-Induced Biophysical Effects to Promote Non-Oncogenic Differentiated Condition

From the insights gained, we wanted to further test whether incubation of LN229 GBM cells with multivalent anti-GM3 IgM antibody can partially mimic the GM3 clustered phenotype as observed at the very low pH. We wanted also to test whether such treatment can enable differentiation of the tumor cells in the −veCD and +veCD, pH 7.4 and 6.2 oncogenic conditions.

**Figure 11:**
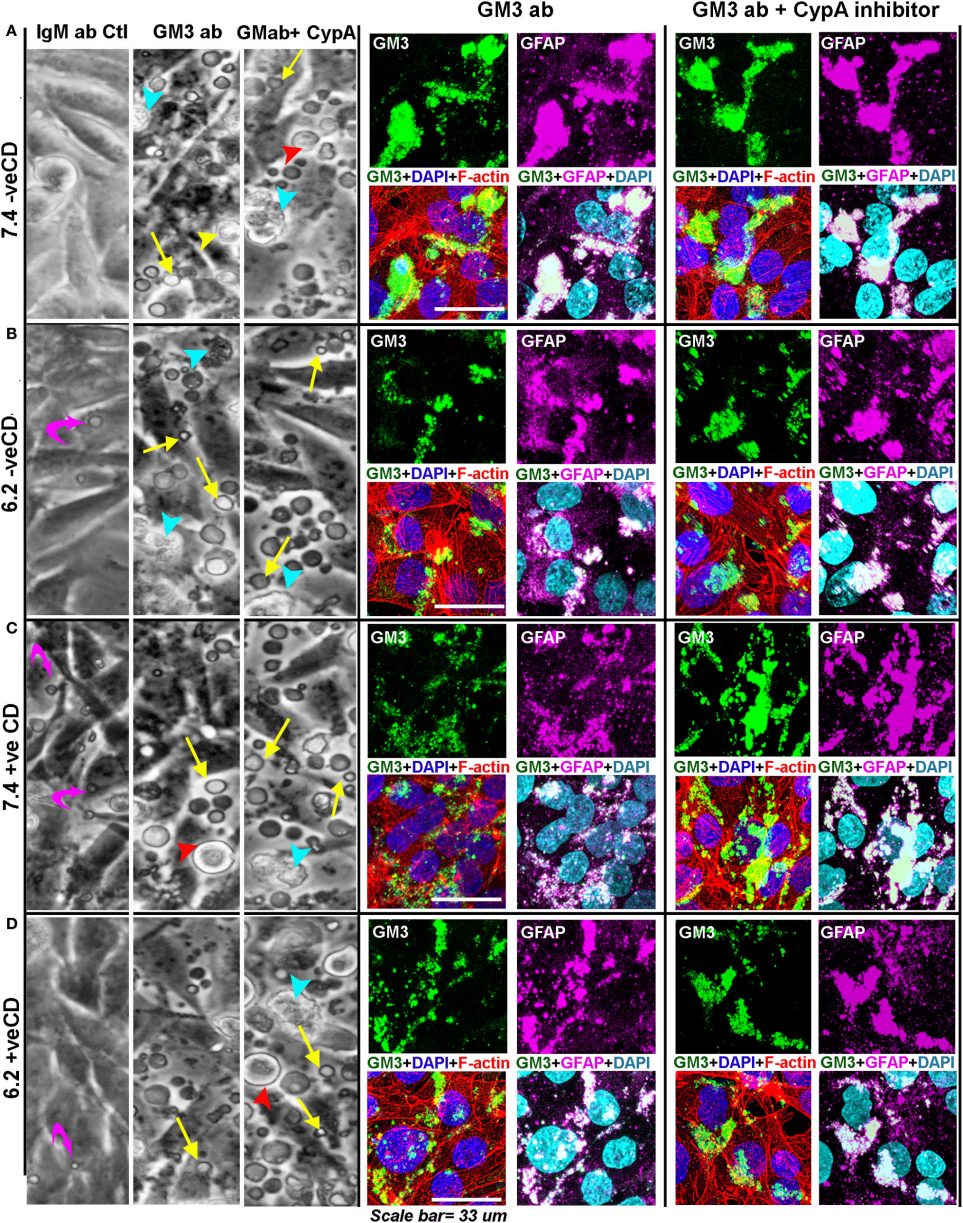
**Anti-GM3 antibody and its additional synergism with cyclophilin A inhibitor mimics low pH-induced physicochemical effects promoting non-oncogenic transformation**. **(A–D)** Cells were incubated with either 20 µg of IgM Ab, anti-GM3 IgM antibody, or anti-GM3 antibody along with 5 nM of cyclophilin A inhibitor. Treatments were repeated after 24–30 h. DIC image panel for each condition shows that by about 33 h, massive cell blebbing (indicated by yellow arrows) was observed in all treatments but not in control antibody sets (also see Figures S14 and S17 in Supplementary Material). The blebbing was rapidly accompanied with cell rounding (red arrowhead), detachment from the substrate (yellow arrowhead), followed by secondary necrosis (cyan arrowhead), and anoikis-type cell death. Cellular built up of acid vacuoles is shown by magenta curved arrows. The anti-GM3 antibody and antibody plus cyclophilin A inhibitor treated cultures were fixed and probed for (i) surface GM3 clusters, (ii) relationship of GM3 clustered organization with cortical and intracellular actin network (*via* Rhodamine phalloidin staining), and (iii) impact of GM3 clusters on the status of differentiation-associated protein (GFAP). Note that the Pearson’s colocalization coefficient (*R*) between GM3 and GFAP in all treatments (anti-GM3 Ab alone or anti-GM3 Ab + cyclophilin A inhibitor) was >0.7 ± SD and hence was significant. The experiment was repeated three times.

For this, we incubated the confluent tumor cell cultures with 20 µg of antibody in 300 µl of medium per well in the eight-well chamber slides. In 24 h, no visible morphological effects were observed over the isotype antibody control. When the next antibody shot was given, within 1.5 h, we began to observe massive cellular blebbing that was further enhanced by the next 3 h (Figure 11A, DIC image panel). The blebs were akin to what were seen at very low pH in our acid stress test (Figure 2B), but the number of levitated blebs and cells were far more in any field of microscopic view suggesting a buildup of high membrane tension which was released by the cells *via* blebbing.

The antibody-incubated cultured cells were fixed and probed for GM3 surface clusters, and the impact of this organization on the cortical and intracellular actin networks as well as on the status of differentiation-associated gene ‘GFAP’ were examined. The cells were fixed within 26 h of antibody treatment (for immunostaining-based analysis) as almost 100% anoikis was observed in 50–60 h post-treatment.

We find that in blebbed conditions (Figure 11), GM3 clusters were formed and were akin to those seen at the very low pH in the acid stress experiments (Figure 10A). These results also suggest that anti-GM3 antibody may have generated GM3 tilts akin to those observed in our simulation studies for the very low pH condition.

The cytoskeleton too was similarly disrupted as observed in the acid stress experiments (Figure S9 in Supplementary Material), and an appreciable increase in GFAP staining was seen (Figure 11). However, the GFAP appeared to be bundled and accumulated in certain regions of the cells, which was not the case when experimental conditions were incubated with isotype-specific IgM antibody alone (Figure S14 in Supplementary Material). These clustered GFAP rich regions were found to be intriguingly enriched in GM3 clusters that were generated by the anti-GM3 antibody-mediated cross-linking. Hence, the cells could be effectively forced into a differentiated state *via* the GM3 antibody-mediated clustering at both physiological and low pHs. The loss of protumorigenic receptors and signaling lattices through extensive surface blebbing could be one of the possible causes for this differentiation-like state.

**Figure 12:**
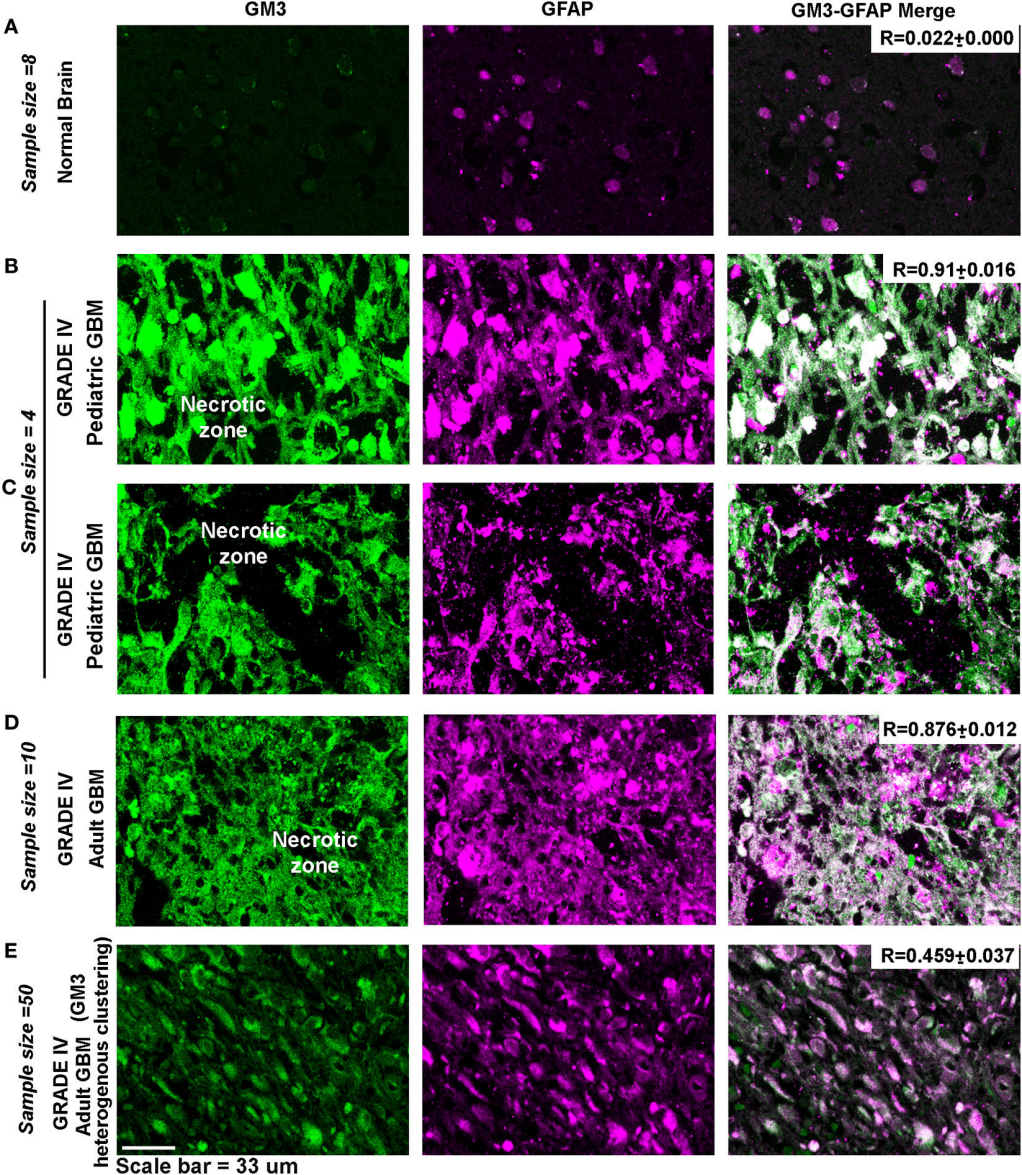
**Necrotic zones in glioblastoma patient samples show surface supraclustering of GM3 and underlying GFAP-positive filaments**. Panel **(A)** shows (human) normal adult brain section with a negligible expression of GM3. GFAP, a filamentous protein and an astrocytic marker, is normally expressed in the brain. Necrotic zone in pediatric glioblastoma multiforme (GBM) patients is shown in panels **(B,C)**, and necrotic zones in adult GBM patient tissue is shown in panel **(D)**. Panels **(B–D)** show high surface clustering of GM3 and GFAP in the corresponding areas. **(E)** Grade IV GBM non-necrotic tumor growth areas (cellular tumor zone) show heterogeneous clustering of GM3 and GFAP unlike supraclustered organization at the necrotic and peri-necrotic zones.

Indeed, necrotic and peri-necrotic zones (associated with low pH environments) in glioblastoma patient samples showed extensive GM3 and GFAP colocalization and supraclustering (Figure 12), which was not seen in the non-necrotic zones of the tumor mass (see *R* values). The clusters in the non-necrotic zones were small and spread throughout the cells or were small and polarized. Hence, these results show that not just GM3 but GM3 supraclustered conformations must be associated with the tumor cell death.

### GM3 Supraclustering along with the Cyclophilin A Inhibition May Be a Useful Non-Cytolytic Antitumor Therapy for GBMs

Inhibition of cyclophilin binding to its surface receptors by cyclosporin A has been evidenced to impact GFAP expression (68). Cyclophilin A is found to be highly expressed in gliomas (Figure S16 in Supplementary Material, GBM patient data on CyPA expression). In our acid stress experiments, cyclophilin A (CyPA) release or secretion was found to be highly reduced at the very low pH and GFAP expression was enhanced (Figure S15 in Supplementary Material; Figure 6D).

We observed that a potent cyclophilin A inhibitor (69) at a very low concentration of 5 nM could inhibit CyPA release and induced appreciable tumor cell blebbing (Figure S17 in Supplementary Material, DIC image inset panels). This was accompanied by an increase in membranous GFAP and GM3 supraclustering; however, the cytoskeleton did not show an extensive rupturing as seen with anti-GM3 antibody treatment. The inhibitor treated cells showed massive stress fibers, indicative of high surface mechanical stress (Figure S17 in Supplementary Material). It is to be noted that the inhibitor treatment was observed to increase the intracellular concentration of cyclophilin A, suggesting an inhibitory action on the extracellular release of CyPA. The time frame for the recording of this observation was as follows: the first inhibitor shot: 24 h; second shot: 3 h.

To enhance the supraclustering effects, we incubated the anti-GM3 antibody and cyclophilin A inhibitor together and found far prominent cell surface blebbing accompanied by an extensive rupture of the cytoskeleton (Figure 11). In this combination treatment, the GFAP expression was more enhanced than in the conditions with the anti-GM3 antibody alone and GFAP showed both surface anchored and sub-surface accumulation in the corresponding GM3 clustered areas.

**Figure 13:**
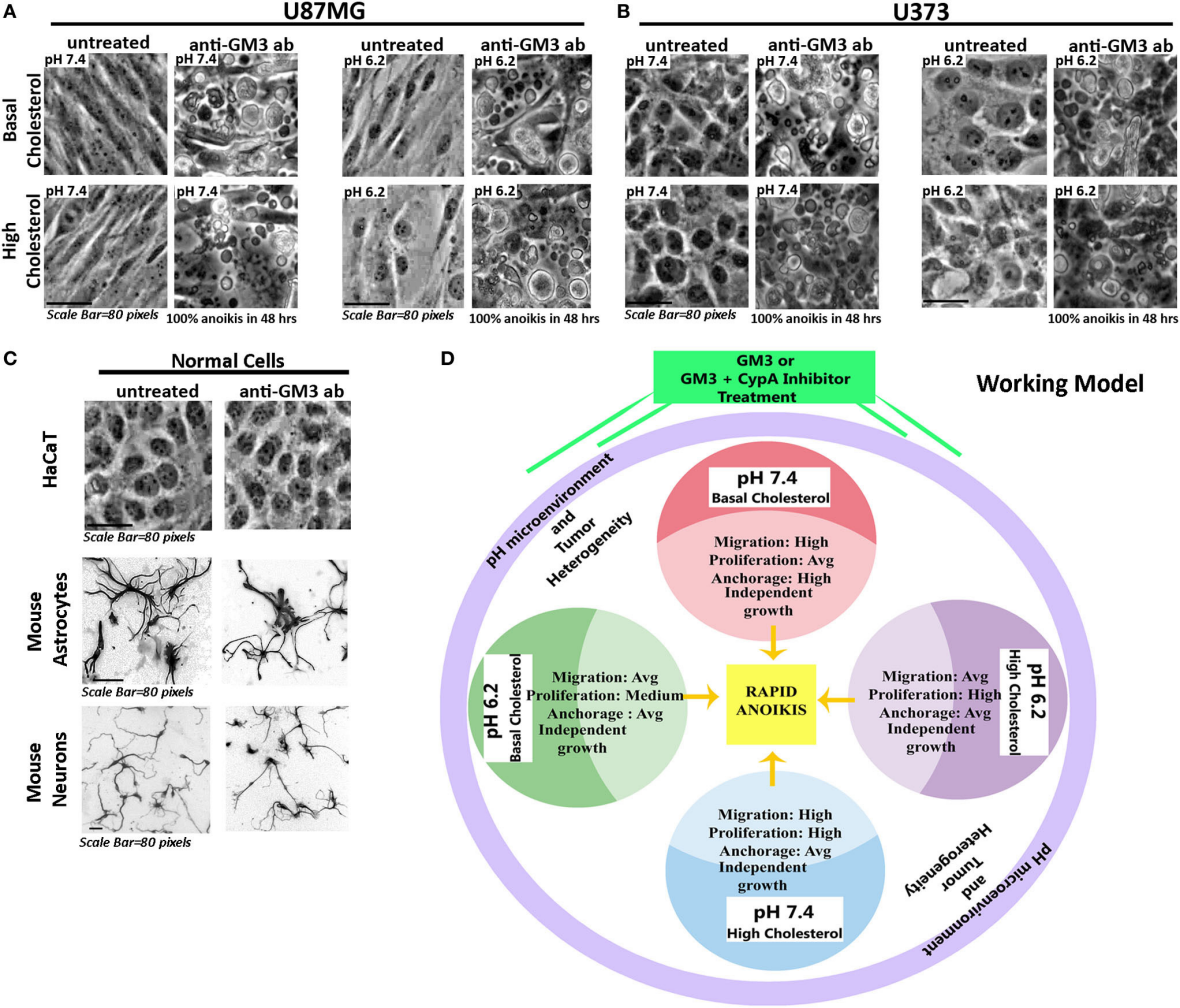
**Experimental confirmation of anti-GM3 antibody-mediated anoikis in other aggressive glioblastoma tumor cell lines**. Glioblastoma cells **(A)** U87MG and **(B)** U373 at oncogenic pH ranges along with **(C)** normal cells: HaCaT (immortalized epidermal keratinocytes), mouse cerebellar granule neurons and mouse cortical astrocytes were treated with anti-GM3 antibody. Results showed 100% anoikis in only glioblastoma cells post 48 h treatment, but no signs of anoikis were visible in normal cells. Note that mouse neurons and astrocytes were fixed after the experiment and labeled with Tuj1 and GFAP antibodies, respectively, to reveal the morphologies clearly. The immunostained images are shown in grayscale. The experiment was repeated three times. **(D)** The deduced working model hence suggests that differential pH-induced cell fate competencies can all collapse into anoikis-driven cell death by regimes that can generate surface GM3 supraclustering/extensive cross-linking.

As GFAP is a membranous and also cytoplasmic cytoskeletal protein, high surface mechanical stress probably influenced its integrity; however, GFAP enhanced expression indicated that cells might have switched their fate to more differentiated phenotypes. Thus a combination of anti-GM3 antibody and cyclophilin A inhibitor was more potent than anti-GM3 antibody alone in first causing cell differentiation and then forcing it to die by anoikis over the course of time (about 30–50 h post treatments). We further find that other GBM cell lines, U87MG and U373, when treated in a like manner, showed similar anoikis phenotype while normal cells such as epidermal keratinocytes (HaCaT), mouse astrocytes, and neurons were unaffected (Figures 13A–C). Hence, indeed manipulation of surface GM3 *via* cross-linking served as an effective therapeutics to eradicate not only low pH zones but also the tumor cells that were growing in physiological pH microenvironments. This combination treatment hence converged the tumor cell heterogeneity into anoikis zones (Figure 13D).

## Discussion and Conclusion

The major goals of this study were (1) to identify how glioblastoma tumor cells respond to changes in the extracellular pH ranges leading to the heterogeneous cell fates; (2) how very low pHe causes growth arrest and (3) whether such identified growth arrest mechanisms can be simulated in the oncogenic pH environments, hijacking their biophysical homeostasis and molecular response machineries, to further transform them into the non-oncogenic phenotype (4) and force them to die by anoikis (a de-adhesion induced cell death), which could be further followed by secondary necrosis that permits better *in vivo* system clearance of the dead cells.

The above mentioned queries were answered by two major findings that generated crucial insights into the role of surface lipids as targets in acidosis and in anticancer therapeutics.

The *first finding* indicated that pHe ranges exhibit differential capacities to modulate glioblastoma cell fate dynamics in a cholesterol-sensitive manner. Data showed that (1) lowering of pHe raised surface cholesterol probably as an adaption to prevent acid hydrolysis of the plasma membrane. A concomitant rise in proliferation index was observed for low pH conditions. (2) Tumor cells at physiological pH, when chemically manipulated to raise surface cholesterol, also showed enhanced proliferation. (3) High cholesterol containing tumor cells in pH microenvironment of 6.2 units showed highest proliferation competencies. Hence, high cholesterol seemed to increase the proliferation potential.

On the contrary, basal cholesterol tumor cells at pH 7.4 showed maximum migration competence and anchorage-independent growth (which is associated with the emergence of more stem-like properties). Biophysical and molecular characterizations suggested that rise in cholesterol may have led to surface rigidity which supports proliferation but de-promotes stem-like characteristics (70, 71). Very low pH at 3.4 units showed very high cholesterol accumulation on the plasma membrane and an immense rise in surface rigidity with the loss of cortical actin and focal contacts. Due to these changes, the ‘very low’ pH exposed tumor cells may have gradually levitated from the surface and died *via* anoikis-a ‘loss of anchorage’ mediated cell death. Interestingly, when the protective umbrella of enhanced surface cholesterol was removed from the low and very low pH microenvironments, the tumor cells underwent rapid anoikis, while physiological pH-adapted cells were unharmed. These results suggest that surface cholesterol plays a crucial role in tumor cell adaptation to acidosis and can be developed into a prognostic and diagnostic marker.

The *second main finding* took leads from the first and describes how extracellular pH levels–cholesterol cross talk impacts the GM3 glycosphingolipid surface clustering, wherein very high proton concentration-driven and cholesterol-sensitive GM3–GM3 ligations were capable of generating strong substrate de-adhesion forces, levitating the tumor cells and hence driving tumor cell fate to anoikis type of cell death. The work further describes how insights from proton concentration-driven GM3 supraclustering mechanisms inspired us to take an alternative approach to generate GM3 supraclusters *via* synergistic application of anti-GM3 antibody to first potentiate the oncogenic tumor cell niches into differentiation and then into subsequent cell death, hence presenting novel insights into tumor therapeutics.

Anti-GM3 antibody probably cross-linked surface GM3 molecules and produced high mechanical pressure as evidenced by blebbing on the tumor surface *via* GM3 supraclustering effects (Figure 11). This effect was accompanied by high expression and clustering of the underlying GFAP-positive filaments and rupturing of F-actin fibers that further diminished the substrate adhesion and levitated the cells. This suggests that due to the probable transbilayer coupling (72), the surface clustering phenomena also clustered the underlying modules and mechano-transduced high pressure stress, damaging the stress fibers, and leading to the non-tumorigenic fate. These levitated cells eventually underwent secondary necrosis and cell death over the course of time. In concert with our results, GFAP high tumor cells are also found to be non-tumorigenic in the animal tumor graft models (73). Indeed, the GM3 supraclustered zones were identified in the necrotic regions of glioblastoma patient samples which also showed extensive GFAP protein clustering (Figure 12).

Cyclophilin inhibitors have been shown to stabilize GFAP (68). Upon the inhibition of cyclophilin A release, we found the phenotypic changes to be akin to those produced by the anti-GM3 antibody treatments; however, the extent of F-actin damage was less. We did not test the mechanism by which CypA directly impacts GM3 clustering. However, GM3 is a well-known lipid raft-associated glycosphingolipid and cyclophilin A interacts with the inner leaflet-associated annexin proteins that are in turn involved in PIP2-mediated lipid raft clustering (74). Given the recent reports that outer and inner membrane leaflets are coupled, ‘intracellularly trapped’ cyclophilin A [*via* use of cyclophilin A release inhibitor (Figure S16 in Supplementary Material)] might have additionally aided in GM3 supraclustering on the outer leaflet.

Indeed, upon the synergistic use of the anti-GM3 antibody and cyclophilin A inhibitor, the effects on anoikis-mediated cell death were enhanced in comparable time points *vs*. individual treatments of the oncogenic pH microenvironments (Figure 11).

The anti and pro-tumorigenic roles of GM3 have been demonstrated *via* the RNA interference studies on GM3 synthase, an enzyme that enables synthesis of GM3 (75–78). Such studies, unfortunately, do not consider the fact that GM3 can be synthesized *de novo* from the higher polysialic acid containing gangliosides *via* the surface resident sialidases that are abundantly expressed in tumor cells (17). Indeed, GM3 and GM3 lactone agonist, as well as sialic acid-clustered GM3 lipid feeding has been shown to cause tumor cell death (66, 79–81). However, the direct feeding of GM3/clustered GM3 for therapeutics is highly undesirable, especially for the brain tumors, as this can be incorporated into the neurons and enough evidence suggest that GM3 in neurons can cause neuronal degeneration (82). This might be the reason why postnatal brain does not synthesize GM3 (83). Therefore, undoubtedly, it is not just the levels or the presence *vs*. absence but the ‘conformational changes of GM3’ on the tumor surface which should be majorly responsible for its differential effects on cell fate and this property should be exploited for anticancer therapeutics.

Given the fact that several tumors express GM3 and cyclophilin A, a therapy composed of anti-GM3 antibody and cyclophilin A inhibitor may not be just limited to brain tumors as demonstrated in this study but may also enable the regression of various tumor types as GM3 is expressed in several other tumors such as melanomas (84, 85), bladder (86), ovarian (75), colorectal (80), colon (87), prostate (88), lung (89), lymphoma (90), hepatic (91), renal (92), and breast cancers (93).

Interestingly, there are growing evidences that majority of tumors engineer acidic microenvironment for survival and invasion and clinical techniques such as acidoCEST MRI are now being employed to measure tumor extracellular pH for prognosis purposes (http://camel.arizona.edu/research/publications) (8, 9). Hence, our pursuit on understanding the cross talk between differential extracellular proton concentration and tumor cell cholesterol–GM3 dynamics, in fine tuning tumor cell heterogeneities and cell fates, should provide a strong impetus to include the measurements of surface cholesterol–GM3 levels in acidoCEST MRI-based prognosis. This may also generate additional insights into combination therapeutics based on the knowledge of proton–lipid–tumor cell fate dynamics.

A recent report (http://meetinglibrary.asco.org/print/2387331, also see 94) also brings forth the importance of manipulating tumor cell pH to induce cell death *via* photodynamically inducing proton release from tumor cells. However, the mechanism by which extracellular protons can manipulate tumor cell surface and downstream cell fate needed elucidation. Hence, our work also mechanistically complements the above report. Our observations further caution that very high concentration of protons will be required for direct acid-driven therapies; otherwise, it may lead to further promotion of oncogenesis (95, 96). Since protons are also diffusible, such direct acid stress therapies may also pose threats to the surrounding normal tissues, if not kept strictly restricted to tumor tissue; hence, the indispensability of mimicking acidosis-driven tumor cell death mechanisms mediated through tumor surface remodeling principles, as demonstrated here needs to be further developed for both solid and liquid tumors toward novel anticancer therapeutics.

## Availability of Data and Material

The datasets supporting the conclusions of this article are included within the article and its supplementary files.

## Ethics Statement

The mouse neurons and astrocyte-associated data were generated on C57/Bl6J mice strictly according to the protocols approved by the Institutional Animal Ethics Committee (certificate number IAEC/164/RM/2012) of Rajiv Gandhi Centre for Biotechnology (RGCB).

## Author Contributions

RM designed the research; SJ and RM performed the research and wrote the manuscript. RM and KS performed molecular simulations. All authors have read and approved the final manuscript.

## Conflict of Interest Statement

The authors declare that the research was conducted in the absence of any commercial or financial relationships that could be construed as a potential conflict of interest. The reviewer CG and handling Editor declared their shared affiliation, and the handling Editor states that the process nevertheless met the standards of a fair and objective review.

## Supplementary Material

Data Sheet 1 **Supplementary experimental procedures**.

Data Sheet 2 **Supplementary figures S1–S17 with legends**.

Figure S1 **GM3 and cholesterol are enriched in the acidic luminal regions of (human) normal kidney and gastric tissues**.

Figure S2 **DNA fragmentation and apoptosis analysis at different pHe in LN229 glioblastoma cells**.

Figure S3 **Autophagy and senescence marker analysis at different pHe in LN229 glioblastoma cells**.

Figure S4 **Comparative staining and intensity analysis of LAMP2 and HMGCR in glioma zones of the same patient tissue**.

Figure S5 **Comparative staining and intensity analysis of LAMP2 and SREBF2 in glioma zones of the same patient tissue**.

Figure S6 **Nystatin-mediated surface cholesterol detection and quantitation**.

Figure S7 **Staining, intensity, and localization analysis of OCT4 in different glioma zones**.

Figure S8 **Staining and intensity analysis of GFAP in different glioma zones**.

Figure S9 **Extracellular pH impacts cytoskeleton-associated remodeling in a cholesterol-sensitive manner**.

Figure S10 **Diagrammatic representation of GM3 tilt angles**.

Figure S11 **Surface GM3 and cholesterol organization in different grades of glioblastoma**.

Figure S12 **Surface profile of GM3 and other gangliosides in LN229 glioblastoma tumor cells**.

Figure S13 **Analysis of GM3 and lactosylceramide surface levels at different pH upon sialidase treatment**.

Figure S14 **IgM control antibody fails to show GM3 and GFAP supraclustering in LN229 glioblastoma cells**.

Figure S15 **Analysis of cyclophilin A in glioblastoma patient samples**.

Figure S16 **Release of cyclophilin A from glioblastoma cell line (LN229) in different pH conditions**.

Figure S17 **Cyclophilin A release inhibitor induces LN229 glioblastoma tumor cell anoikis via GM3 and GFAP supraclustering**.
