# Supplementary material for: Extracellular Proton Concentrations Impacts LN229 Glioblastoma Tumor Cell Fate via Differential Modulation of Surface Lipids
Source: Front Oncol. 2017 Mar 1;7:20. doi: 10.3389/fonc.2017.00020 (PMC5331044; doi:10.3389/fonc.2017.00020)
Supplement: Data Sheet 1 — Supplementary experimental procedures. [file Data_Sheet_1.pdf]

SUPPLEMENTARY METHODS AND MATERIALS

Research Article Title:

Extracellular proton concentrations impacts LN229 glioblastoma tumor cell-fate *via* differential modulation of surface lipids

Authors:

Sebastian John[1], K.C. Sivakumar [2] and Rashmi Mishra[1]*

Authors Affiliations:

[1]*Disease Biology Program, Department of Neurobiology and Genetics, Rajiv Gandhi Centre for Biotechnology, Thycaud PO, Poojappura, Thiruvananthapuram, India-695014.* [2]*Distributed Information Sub-Centre, Rajiv Gandhi Centre for Biotechnology, Thycaud PO, Poojappura, Thiruvananthapuram, India-695014.*

*Corresponding author: Rashmi Mishra, Email: rashmimishra@rgcb.res.in, Tel: +918750347930

CONTENTS:

1) Molecular Simulations Page-3

2) Human tissue array Immunohistochemistry Page-4

3) Cell Surface assays Page-5

(i) Anti-GM3 and anti-Lactosylceramide labeling

(ii) Nystatin mediated cholesterol measurement

(iii) Anisotropy measurements

4) Cell membrane tension measurements in 3D cultures with Gel Contraction assays.Page-6

5) Intracellular pH measurements with pH Rodo probe Page-6

6) Glucose uptake assay Page-7

7) Cell growth associated Soft Agar assay Page-7

8) Cell senescence assay Page-7

9) DNA Fragmentation Assay Page-8

10) Dextran TMR- Fluid phase uptake Page-8

11) Wound healing associated migration assay Page-8

12) Immunocytochemistry Page-9

13) Antibody details Page-9

14) Neuraminidase treatment/ Top lactonization estimation assay Page-9

15) F-actin staining Page-10

16) Antibody treatment Page-11

17) Human Protein Atlas (HPA) cancer data extraction and analysis Page-11

18) Cyclophilin A Inhibitor Treatment Page-11

19) Isolation of mouse cerebellar neurons and cortical astrocytes Page-12

20) Image capture settings Page-12

21) Images analysis and statistics Page-12

22) Image Presentation Page-13

23) Statistical analysis Page-13

24) References Page-14

METHODS AND MATERIALS

1) Molecular Simulations: We have carried out a series of atomic-scale molecular dynamics simulations on lipid bilayer comprising of 16 AcGM3 and 146 POPC molecules for three different pH conditions: 7.4, 6.2 and 3.4 units. This allowed us to study the conformation and organization of glycan headgroup of GM3 on bilayer normal at various pHs in a systematic manner.

The simulation was performed using the GROMACS molecular dynamics package (Hess et al., 2008; Van Der Spoel et al., 2005; Lindahl et al., 2001; Berendsen, Sep-1995). The initial configuration of the simulation was prepared by arranging the POPC molecules in a regular array in the bilayer XY plane using the Gromacs command such as editconf and genconf. In the upper leaflet of the membrane, 16 POPC molecules were removed and 16 GM3 molecules were placed into the cavities created this way, with the axis of the GM3 aligned to the membrane normal and the headgroup sticking out of the membrane surface. The position of neighboring GM3 molecules on the XY plane was chosen in such a way so that their tails could not interact directly with each other. The bilayer systems were hydrated with approximately 10,966 water molecules. For (Ac) GM3 molecules, OpenBabel was used to apply correct charges for a given pH. To parameterize the molecules, all-atom OPLS force field extended for carbohydrate simulations (Damm, 1997) were used. Partial charges, charge groups and neutral units were originally derived in the OPLS parameterization.

A rectangular basic simulation box was used with standard periodic conditions. For water, TIP3P model which is compatible with the OPLS parameterization was employed. The length of the three box edges was allowed to fluctuate independent of each other, in order to decouple the equilibration of the diagonal pressure tensor components and the internal stress tensor was allowed to relax accordingly. The temperature and pressure were kept constant by weak coupling using Berendsen method (Berendsen et al., 1984) with relaxation times set to 0.4 and 1.0ps respectively. The temperature of the solute and solvent were controlled independently. The semi- isotropic control was applied to regulate the pressure. The bond length of the POPC and GM3 molecules were kept constant by means of the LINCS

algorithm (Hess, 1997), while those of the water molecules were kept constant using the SETTLE methods (Shuichi Miyamoto, 1992). The LINCS algorithm was used to preserve hydrogen covalent bond lengths. The Lennard-Jones interactions were cut off at 1.0nm, and for long range part of the electrostatic interactions were accounted by employing the smooth particle mesh Ewald method with a mesh spacing of 12Å. All site-site interactions were truncated to zero beyond the cut-off distance of 9.0Å.

Prior to the actual MD simulations, the steepest-descent algorithm was used to minimize the energy of the initial structure. In the entire course of the simulation, the integration time step of 2fs was used. The MD simulations of all membrane systems were carried out for 200ns. The first 100ns were considered as an equilibrium period. Consequently, only the last 100ns of the trajectory were analyzed. The simulation procedure used in this study has been effectively applied in numerous molecular dynamic simulation studies on lipid bilayers (Patra et al., 2004; Falck et al., 2004b;a).

2) Human tissue array Immunohistochemistry:

Pre-processing: The tissue array slides purchased from US Biomax Inc. were as follows: Glioblastoma (GL805a, with 63 GBM Grade IV patient samples), Human Normal Kidney Tissue (K D803) and Human Normal Stomach Tissue (BN01013a). The slides were pre-baked at 55°C on a hot plate for 15 minutes and then were incubated in fresh xylene for 10 minutes for de-paraffinization. The excess xylene was slowly drained and the slide was re-incubated in fresh xylene for 10 minutes again. Followed by xylene treatment, the slides were first immersed in chloroform for 5 min to remove any xylene and then immersed in 100%, 90%, 80% and 70% ethanol (5 minutes each) for rehydration. The tissues were washed (twice) thereafter in phosphate buffer saline (pH 7.4) and then permeabilized using 0.01% digitonin (in PBS) for 30 min at room temperature. The slides were then washed 5-6 times with PBS and prepared for immunofluorescence.

Immunofluorescence: The tissues were incubated with blocking buffer (3% BSA/PBS with 10% Donkey Serum) for 1 hour and 30 minutes. After blocking, the tissue arrays were washed with 1X PBS and were then incubated with primary antibody (anti GM3, 1:50, anti Cholesterol, 1:100 and anti GFAP, 1:100) and kept for 36 hours at 4°C. Post incubation, the slides were properly washed with PBS (pH 7.4) and were further incubated with secondary antibodies for 90 minutes at RT. The slides were washed properly with 1X PBS and were

DAPI stained for 5 minutes before mounting them in 70% glycerol. In glioblastoma tissue-based analysis, 3 independent arrays with the same Cat. no. GL805a, i.e., tissues from the same patients were processed to represent 3 technical replicates.

3) Cell Surface assays:

LN229 tumor cells were cultured in 8 well chamber slides and after each of the experimental end points, the cells were washed in PBS++ (with 1mM Calcium Chloride and 1mM Magnesium Chloride) to inhibit cell de-adhesion.

(i) *Anti-GM3 or anti-Lactosylceramide labeling:* Either anti-GM3 (1:150) or anti-Lactosylceramide (1:150) antibody was applied on the surface in cold for one hour and cells were then washed in 1XPBS++ and fixed with cold 1.5% PFA. The secondary antibodies (1:200) were subsequently applied for 1 hour without permeabilization. Cells were later mounted in 70% glycerol-based mounting media and imaged.

(ii) *Nystatin mediated cholesterol measurement:* First nystatin (50µg/ml) was incubated on cells for 40 minutes on ice. Cells were further washed and mounted in Prolong mountant that contains an antifade. All experiments were performed in triplicates and 5 random images were captured for further quantitations.
In another experimental set, cells were first fixed with 1.5% PFA at RT and then nystatin staining was followed. For spectrofluorimetric analysis, the cells in different treatments were first labeled with nystatin for merely 3-4 minutes to prevent endocytosis. The cells were quickly washed and scrapped in 1XPBS and processed for standard fluorimetric analysis. The recordings were taken in Perkin-Elmer LS50 spectroscope ex/em 320/410 with an excitation emission slit of 5 nm at 22˚C.

(iii) Anisotropy measurements: Ten thousand cells were seeded in 40mm dishes for 24 hours and pH treatment was given as described above. At the end of the experiment, cells were washed in warm IX HBSS and incubated with 1,6 Diphenyl-1,3,5-Hexatriene (DPH), 2µM, for 1 hour at 37°C. Cells were further washed and gently scrapped with a cell scraper, collected in a 15ml tube, centrifuged at 2000rpm for 3min and re-suspended in 1 ml of 1XPBS. Anisotropy was measured in Perkin-Elmer LS50 spectroscope (with polarizers) using the following setting: Excitation wavelength of 365nm and emission wavelength of 430

nm, Em. and Ex. slit was kept at 5nm and a temperature of 37^0C. The fluorescence anisotropy (r) value was calculated using the equation $r = [(I|| − I⊥) / (I|| + 2I⊥)]$, where I|| and I⊥ are the fluorescence intensities oriented parallel and perpendicular, respectively, to the direction of the polarization of the emitted light.

4) Cell membrane tension measurements in 3D cultures with Gel Contraction assay:

Cells were trypsinized, counted and seeded into a 1 mg/ml Type I collagen solution (Invitrogen) in growth media at a concentration of 1×10^5 cells/ml in four-well culture dishes. The collagen/cell suspension was mixed with pipette in a ratio of 1:2, and 1 ml was added to each well and the solution was allowed to polymerize for 45 min at 37°C. Fresh growth medium was added to the solidified collagen gels and plates were returned to the incubator for 24 hours. Next day, the pH experiment was performed as described earlier and at the end point of the experiment collagen gel was detached from the sides of the well and allowed to freely float and contract in the respective conditions. Gel contraction was monitored over a period of 24 hours and the surface area of contracted gels was measured using Image J. The percent reduction in surface area was calculated by using the following formula % gel contraction=(Original surface area – Final surface area / Original surface area)*100. The results were represented as % gel contraction.

5) Intracellular pH measurements with pH Rodo probe:

Cells were plated on glass bottom dishes and incubated for 16 hours before treatment. After incubation, the cells were serum deprived for 1 hour and pH treatment was followed as discussed earlier. Post treatment, the cells were washed with HBSS (pH 7.4). After washing the cells were incubated with pH Rodo AM staining solution (1:10:1000; staining solution: powerload concentrate: HBSS) at 37°C for 45 minutes in incubator. After incubation, the cells were washed once with HBSS and were imaged at Ex. /Em of 509/533.

Standard Preparation

Various pH standards were prepared for standard curve. Cells plated in glass bottom dishes were treated with various pH mediums for 3 hours. Post pH treatment, the cells were stained with pH Rodo AM as discussed above. After staining, the cells were treated with valinomycin (10μM) at 37°C for 5 minutes before imaging.

6) Glucose uptake assay:

Cells post treatment were washed in warm 1XHBSS buffer, without glucose, and incubated with 100µM of 2-NBDG (2-(N-(7-Nitrobenz-2-oxa-1,3-diazol-4-yl)Amino)-2-Deoxyglucose) for 20min and further washed and left for additional 20min. Cells were then imaged live. 5 images were captured per well per condition to average the readings. The experiment was performed in triplicates and average glucose uptake was represented in percentage for each condition in a graphical format.

7) Cell growth associated Soft Agar assay:

Anchorage-independent and clonogenic growth was estimated through the soft agar assay. Briefly, 0.5% agarose containing DMEM medium supplemented with FBS was added in 6 well cell culture dishes and allowed to solidify (base agar) at RT. After 30mins 1.2×10^4 LN229 cells were mixed with 0.35% agarose containing DMEM medium supplemented with FBS and was added to the top of base agar. The cells were then cultured for 7 days at 37°C in 5% carbon dioxide. The cells were then given the pH treatment and colony growth was imaged over further 12 days. Medium in respective conditions was replaced every 24 hours. The experiments were done in triplicate. The average diameter between initial and final time point over 15 colonies in each condition was derived and represented in graph using the following formula: % sphere growth= (final sphere size-initial sphere size/initial sphere size)*100.

8) Cell senescence assay:

The assay was performed as per manufacturer's protocol. For the positive control, cells were first treated with 400µM H_2O_2 for 2 hours and were further replaced with normal medium for additional 5 hours. Cells were incubated for 12 hours in beta-galactosidase substrate solution at 37˚C on hot plate and, excessive crystals were dissolved with a wash in DMSO. The blue colored enzyme-substrate complex formation was imaged and 5 random fields from each condition (in duplicates) were captured for analysis.

9) DNA Fragmentation Assay:

The pH experiments were performed as described above. After the extraction, purification and concentration determination of DNA from all the samples, the ladder assay was performed according to a recent protocol (Rahbar Saadat et al., 2015). The samples were run on a 1.2% agarose gel primarily at 15 volts for 5 minutes continued by 120 volts for next 30 min.

Positive control cells were either treated with 200µM Temozolomide or with 1 mM H2O2. Later the gel was visualized and imaged under UV transilluminator after ETBR staining.

10) Dextran TMR- Fluid phase uptake:

$8x10^4$ cells were plated in 8 well chamber slide containing DMEM, 10%FBS and 1X antibiotic. Cells were pH treated as discussed earlier. Post treatment, the cells were incubated with 2mg/ml Dextran TMR for 10 minutes. Post incubation, the cells were washed with PBS with calcium and magnesium and were fixed with 1.5% PFA. The cells were imaged using A1R confocal microscope and the signal intensity was quantitated using Fiji Image analysis software.

11) Wound healing associated migration assay:

$4.8x10^4$ cells were cultured in 24 well platforms. At the end of the experiment, a plastic pipette tip was used to draw a scratch across the center of the culture to produce a clean wound area. The images at the beginning and in every 12 hours interval for a total of 72 hours were captured by light microscopy. The distance between the edges of the wound was estimated from the captured images using line measure tool in Fiji software. The graph was plotted against distance covered over time. Speed for each condition was hence derived and represented in µm/sec and indicated in the graph. During the migration assay, medium in each condition was replaced per 24 hours with the respective conditions.

12) Immunocytochemistry:

Cells were fixed for 20 min in 1.5% PFA, permeabilized with 0.25% saponin for 20 min, blocked for 1 hour with 5% BSA containing 2% normal serum in PBS and primary antibody

was then incubated overnight at 4°C at the indicated concentrations below. Following primary antibody, cells were washed with PBS and incubated with anti-isotype specific Alexa-fluor conjugated secondary antibody (Jackson immunoresearch) at a concentration of 1: 200 in 1X PBS for 1 h at room temperature in dark. Cells were coverslipped with mounting medium containing DAPI (Invitrogen) and images were captured using a Nikon confocal microscope in 60X oil immersion objectives with NA 1.29.

13) Antibody details:

Lipid antibodies: GM3 (CosmoBio; NBT-M101; 1:150), Lactosylceramide (Aviva; OAAD00051; 1:150), SSEA4 (Novex; cat no.414000; 1:50), anti-Cholesterol (Abbexa; abx100311; 1:100).
Proliferation markers: Oct4 (sc5279,1:100), BrdU (sc70443); *Apoptosis Markers:* Cleaved caspase-3 (IMG5700,1:100), cleaved caspase 8 (IMG5703, 1:100) PARP gamma (sc7196;1:100); *Autophagy markers:* Beclin-1 (sc11427,1:80), LC3II (sc134226,1:100); *Energy metabolism markers:* cAMP (ab24851,1:200), LDHA (sc33781;1:50); *Cell differentiation markers:* GFAP (sc- 6170,1:200), *Adhesion and Cytoskeleton markers:* Rac1 (Cytoskeleton inc., ARC03-A;1:100), Beta 1 integrin (sc13590,1:100); Cytoskeletal and *Cell migration markers:* alpha tubulin (sc8035;1:100), vinculin (sc73614;1:200), cortactin (ab51073,1:200), alpha-actinin (ab81265;1:200); *Other antibodies and probes:* human IgM (ab26867;1:100), Cyclophilin A (ab58144;1:300), WGA (Invitrogen; w11262).

14) Neuraminidase treatment/ Top lactonization estimation assay:

This assay was performed to determine, whether GM3 surface clusters were due to GM3 lactone formation under low pH condition or were predominantly due to the net effect of GM3-GM3 surface ligations. However, it is to be noted that GM3 lactones are formed only to some extent under low pH condition and they are not observed at physiological pH (Alves et al., 2002).
0.75 x 10^5 cells were plated on 8 well-chambered slides in DMEM with 10%FBS and 1X antibiotic and incubated for 16 hours. After incubation, the cells were serum deprived for 1 hour and pH treatment was followed as discussed in earlier sections. Post treatment, the cells were either further treated with methyl cyclodextrin (10uM) for 2 hours (+veCD condition) or left as such (-veCD condition) in the respective pH grades before incubating the cells with

neuraminidase. Cell treatment with neuraminidase was then followed as described:
Cells were first washed with PBS plus (calcium + magnesium) and then treated live with 0.25U/ml neuraminidase at (i) 37°C and (ii) on ice in PBS plus for further four hours according to established protocols (Zhu et al., 1999). After treatment, cells were fixed with 1.5% paraformaldehyde and probed for surface GM3 and Lactosylceramide by immunocytochemistry, wherein antibodies to the respective lipids were incubated on cell surface without permeabilizing the cells and signal were detected with fluorescently labeled secondary antibody. For confocal based quantitation of the levels of lipids concerned, the confocal settings were adjusted with no primary control and all settings were kept identical for image capture under each condition. The experiment was repeated in triplicates and the signals from the images (approx. 200 cells) were quantified using 'measure' tool in Fiji Image Analysis software. The cell peripheries were outlined from the DIC images of the respective fields and superimposed on the fluorescent images using ROI tool in Fiji Image Analysis software. Since GM3 lactones, formed under low pH condition, are demonstrated to be 'resistant to neuraminidase treatment,' the extent of retention of GM3 signal on the cell surface shows the extent of lactonized GM3 under low pH (6.2 and 3.4 units). Since loss of neuraminic acid from GM3 leads to lactosylceramide, the increase in lactosylceramide signal demonstrates the extent of GM3 that was not lactonized on the surface. Hence, this assay can enable detection of the extent of GM3 surface lactone formation under low pH conditions. Probably due to near optimal activity of neuraminidase/sialidases at the room temperature, the signal intensities for lipid concerned were lower than those obtained with cells treated on ice (cold treatment).

15) F-actin staining:

Fixed cells were first processed for immunocytochemistry if needed, then were further incubated in Rhodamine phalloidin at a concentration of 2µg/ml for 45 min. Cells were briefly washed and counter stained with Hoechst 33342 (5µg/ml for 5min) and mounted in the mounting media.

16) Antibody treatment:

$0.7x10^5$ cells were seeded in 8 well chamber slides with DMEM, 10%FBS, 1X antibiotic. The cells post 16 hours incubation were treated with pH conditions as described in previous

sections. Post-treatment the cells were treated with 20µg of anti-GM3 monoclonal IgM antibody in 250µ l of medium and were left incubated for further 24 hours. After 24 hours the cells were retreated with antibody for further six hours and then fixed with 1.5% PFA at RT for 30 minutes.

Immunofluorescence: The fixed cells were properly washed 3-4 times with 1XPBS (pH 7.4) and then incubated with anti-IgM secondary antibody for 90 minutes at room temperature. The cells were then properly washed with 1X PBS and permeabilized with 0.01% digitonin. After permeabilization, the cells were blocked with 5% BSA with 2% donkey serum. After blocking, the cells were incubated with primary antibody for GFAP for next 16 hours at 4°C. Following this step, the regular immunofluorescence method as discussed earlier was performed.

17) Human Protein Atlas (HPA) cancer data extraction and analysis:

Glioma (high-grade) patient tissue section images, immunostained for respective proteins of interest were extracted from HPA data portal (Ponten et al., 2008;Uhlen et al., 2010). This process was carried to extract the extent of corroboration of our *in vitro* observations in acid stress experiment with proteomics remodeling in glioma patient tissues. Individual images were further qualitatively evaluated for two parameters, i.e., their staining on a scale of high, medium, low and intensities on a scale of strong, medium and weak in a tissue region-specific manner. The tumor-associated major anatomic structures were chosen in concordance to the specified anatomic structures specified in IVY glioblastoma atlas project 2015 (IvyGAP, 2015) (http://glioblastoma.alleninstitute.org/, follow 'documentation tab' and 'overview' under document sub-section).

18) Cyclophilin A Inhibitor Treatment:

Post pH treatment, the cells were treated with 5nM of Cyclophilin A inhibitor in 250µl of medium for 24 hours. After 24 hours the Cyclophilin A inhibitor treatment was repeated in a fresh 250µl of media. After 48 hours the cells were fixed with 1.5% PFA and relevant assay was followed.

19) Isolation of mouse cerebellar neurons and cortical astrocytes:

The mouse cerebellar neurons and the cortical astrocytes isolation were performed using previously described protocols (Lee et al., 2009; Schildge et al., 2013) from P4-P9 post natal pups.

20) Image Capture settings:

The followed protocols were similar to that described in Rharass, T. et al, JBC (2014) (Rharass et al., 2014). Cell morphologies were observed in transmitted light to ensure no cell death occurred during the experimental time frame. The image capture setting, namely, detector gain and offset, pinhole size, laser power, confocal section, zoom factor, line and frame averaging were kept constant for all comparative set of experiments. Brightness/contrast adjustments were applied equally to every pixel in the images (i.e. maximum projections), for each comparative set using Fiji image analysis software. No change to gamma settings was applied. Regions of interest (ROIs) were set individually i.e. for each cell of the population in the images, based on cell boundaries to calculate the mean fluorescence intensities (ratio of the sum of fluorescence intensity emitted in the ROIs to number of pixels in the ROIs). All data were obtained from at least 3 independent experiments. For each time point or treatment, at least 10 images per experiment were recorded. Results are shown as means ± S.D.

21) Images analysis and statistics:

Protein integrated densities: Protein profile in cytoplasmic and nuclear fractions of the cell were derived either by using free hand tool ROI followed by 'Measure' application in Fiji software or by using the Cell Profiler software.

Co-localization: The image statistics were produced by the colocalization Coloc2 plug-in in Fiji Software (freely downloadable from- http://pacific.mpi-cbg.de/). Briefly, we used the following measure: R= Pearson's colocalization coefficient for the image.

Cluster analysis: The cluster signals were identified against the background using the threshold and binary tools embedded in Fiji Image analysis software. Subsequently, the

Measure tool was used to retrieve the area and intensity values of each cluster. The area values were in micrometer square and the integrated density designated the number of pixels in each cluster. The minimum size of cluster was set automatically by the software and was described by a unit of $0.17\mu m^2$.

22) Image Presentation:

In most data figures, the images were presented as pseudo-colored index images, using fire LUT. The lowest to highest intensity of expression, at a single cell and tissue level, can be easily visualized by this format using the calibration bar as a guidepost. On some occasions, the DIC images were given a pseudo-color tinge in Adobe Photoshop, using Image>Adjust>Color Balance tools. This was merely done to distinguish the sub-panels within panels in some figures. It does not distort the quality and meta-data associated with the images.

23) Statistical analysis:

The multivariant test was performed using ANOVA in Microsoft Excel. Statistical analyses were performed using two-tailed unpaired Bonferroni's t-test. Significance was represented by the following p-values: $^{*}p \leq 0.05$, $^{**}p \leq 0.01$ and $^{***}p \leq 0.001$. All comparisons were made with pH 7.4 -veCD conditions unless otherwise indicated. Data was presented as means ± S.D. and averaged from at least 3 independent experiments. For single cell analysis, at least 50-200 cells captured in 5 random fields per condition in 3 independent experiments were considered.

References:

Alves, F., Borchers, U., Keim, H., Fortte, R., Olschimke, J., Vogel, W.F., Halfter, H., and Tietze, L.F. (2002). Inhibition of EGF-mediated receptor activity and cell proliferation by HK1-ceramide, a stable analog of the ganglioside GM3-lactone. *Glycobiology* 12**,** 517-522.

Berendsen, H.J.C., Postma, J.P.M., Van Gunsteren, W.F., Dinola, A., and Haak, J.R. (1984). Molecular dynamics with coupling to an external bath. *The Journal of Chemical Physics* 81**,** 3684-3690.

Berendsen, H.J.C., Van Der Spoel, D. & Van Drunen, R (Sep-1995). GROMACS - A

MESSAGE-PASSING PARALLEL MOLECULAR-DYNAMICS IMPLEMENTATION. *Computer Physics Communications* 91**,** 43-56.

Damm, W., Frontera, A., Tirado–Rives, J. And Jorgensen, W. L. (1997). OPLS all-atom force field for carbohydrates. *J. Comput. Chem.* 18**,** 1955–1970.

Falck, E., Patra, M., Karttunen, M., Hyvonen, M.T., and Vattulainen, I. (2004a). Impact of cholesterol on voids in phospholipid membranes. *J Chem Phys* 121**,** 12676-12689.

Falck, E., Patra, M., Karttunen, M., Hyvonen, M.T., and Vattulainen, I. (2004b). Lessons of slicing membranes: interplay of packing, free area, and lateral diffusion in phospholipid/cholesterol bilayers. *Biophys J* 87**,** 1076-1091.

Hess, B., Bekker, H., Berendsen, H. J. C. And Fraaije, J. G. E. M. (1997). LINCS: A linear constraint solver for molecular simulations. *J. Comput. Chem.* 18**,** 1463–1472.

Hess, B., Kutzner, C., Van Der Spoel, D., and Lindahl, E. (2008). GROMACS 4: Algorithms for Highly Efficient, Load-Balanced, and Scalable Molecular Simulation. *J Chem Theory Comput* 4**,** 435-447.

Ivygap (2015). *Technical White Paper Overview* [Online]. Available: glioblastoma.alleninstitute.org [Accessed May,2015].

Lee, H.Y., Greene, L.A., Mason, C.A., and Manzini, M.C. (2009). Isolation and culture of post-natal mouse cerebellar granule neuron progenitor cells and neurons. *J Vis Exp*.

Lindahl, E., Hess, B., and Van Der Spoel, D. (2001). GROMACS 3.0: a package for molecular simulation and trajectory analysis. *Molecular modeling annual* 7**,** 306-317.

Patra, M., Karttunen, M., Hyvönen, M.T., Falck, E., and Vattulainen, I. (2004). Lipid Bilayers Driven to a Wrong Lane in Molecular Dynamics Simulations by Subtle Changes in Long-Range Electrostatic Interactions. *The Journal of Physical Chemistry B* 108**,** 4485-4494.

Ponten, F., Jirstrom, K., and Uhlen, M. (2008). The Human Protein Atlas--a tool for pathology. *J Pathol* 216**,** 387-393.

Rahbar Saadat, Y., Saeidi, N., Zununi Vahed, S., Barzegari, A., and Barar, J. (2015). An update to DNA ladder assay for apoptosis detection. *BioImpacts : BI* 5**,** 25-28.

Rharass, T., Lemcke, H., Lantow, M., Kuznetsov, S.A., Weiss, D.G., and Panakova, D. (2014). Ca2+-mediated mitochondrial reactive oxygen species metabolism augments Wnt/beta-catenin pathway activation to facilitate cell differentiation. *J Biol Chem* 289**,** 27937-27951.

Schildge, S., Bohrer, C., Beck, K., and Schachtrup, C. (2013). Isolation and Culture of Mouse Cortical Astrocytes. e50079.

Shuichi Miyamoto, P.a.K. (1992). SETTLE: an analytical version of the SHAKE and

RATTLE algorithm for rigid water models. *Journal of Computational Chemistry* 13**,** 952-962.
Uhlen, M., Oksvold, P., Fagerberg, L., Lundberg, E., Jonasson, K., Forsberg, M., Zwahlen, M., Kampf, C., Wester, K., Hober, S., Wernerus, H., Bjorling, L., and Ponten, F. (2010). Towards a knowledge-based Human Protein Atlas. *Nat Biotech* 28**,** 1248-1250.
Van Der Spoel, D., Lindahl, E., Hess, B., Groenhof, G., Mark, A.E., and Berendsen, H.J. (2005). GROMACS: fast, flexible, and free. *J Comput Chem* 26**,** 1701-1718.
Zhu, J., Li, Y.T., Li, S.C., and Cole, R.B. (1999). Structural characterization of gangliosides isolated from mullet milt using electrospray ionization-tandem mass spectrometry. *Glycobiology* 9**,** 985-993.
