# Supplementary material for: Extracellular Proton Concentrations Impacts LN229 Glioblastoma Tumor Cell Fate via Differential Modulation of Surface Lipids
Source: Front Oncol. 2017 Mar 1;7:20. doi: 10.3389/fonc.2017.00020 (PMC5331044; doi:10.3389/fonc.2017.00020)
Supplement: Data Sheet 2 — Supplementary figures S1–S17 with legends. [file Data_Sheet_2.pdf]

# SUPPLEMENTARY FIGURES S1-S17 WITH LEGENDS

Research Article Title:

# Extracellular proton concentrations impacts LN229 glioblastoma tumor cell fate *via* differential modulation of surface lipids

Authors:

Sebastian John[1], K.C. Sivakumar [2] and Rashmi Mishra[1]*

Authors Affiliations:

[1]*Disease Biology Program, Department of Neurobiology and Genetics, Rajiv Gandhi Centre for Biotechnology, Thycaud PO, Poojappura, Thiruvananthapuram, India-695014.* [2]*Distributed Information Sub-Centre, Rajiv Gandhi Centre for Biotechnology, Thycaud PO, Poojappura, Thiruvananthapuram, India-695014.*

*Corresponding author: Rashmi Mishra, Email: rashmimishra@rgcb.res.in, Tel- +91- 8750347930

## SUPPLEMENTARY FIGURE LEGENDS

**S1 Fig: GM3 and cholesterol are enriched in acidic luminal regions of (human) normal kidney and gastric tissues.**

**A)**, **B)** Shows normal tissue sections from human kidney **C)**, **D)** Shows normal tissue sections from human gastric regions. The DAPI stained DNA in cells lining the acidic lumens showed typical circular plasmid like chromatin organization. The cells lining the lumens were observed to be highly enriched in cholesterol as visualized with anti-cholesterol antibody immunostaining (shown in red). The 'cholesterol-enriched cells' also showed significant colocalization with GM3 ganglioside (shown in green), a sphingolipid that protects the apical membranes of luminal epithelial cells from acid stress. Please see co-localization coefficient, R-values. The immunostained images of both lipids show highly punctate and clustered organization that may be induced by high proton concentrations in the lumen.

**S2 Fig: DNA fragmentation and Apoptosis analysis at different pHe in LN229 glioblastoma cells.**

**A)** DNA fragmentation assay was performed 24 hours post pH treatments, to detect apoptosis. Data showed no signs of DNA damage and laddering in the pH treated samples. Note that the treatment with Temozolomide (an FDA approved anti-glioma drug) and high concentrations of $H_2O_2$ induced partial DNA laddering and nucleic acid smearing, hence served as the positive controls in this assay. **B), C)** and **D)** Shows expression profiles of apoptosis-associated proteins. Cleaved PARP1 was weakly expressed at pH 3.4, cleaved caspase-8 too showed lower expression at pH 3.4 in comparison to other pHs but cleaved caspase-3 showed higher expression at pH 3.4. However, the data overall did not show any appreciable and consistent trendlines at very low *vs.* low and physiological pH conditions, suggesting that cells were not progressing towards apoptosis. It is to be noted that cleaved caspases are associated with non-apoptotic functions too. Also note that cleaved caspases were observed to be sequestered near the inner surface of the plasma membrane at low and very low pHs, a localization that cannot induce apoptosis. The absence of tumor cell apoptosis at various pHe was also in concert with the lack of DNA fragmentation and nuclear pyknosis (**Fig 2D**) in all pH ranges. Besides, the positive response of tumor cells in live cell-based assays such as glucose uptake, gel contraction and macropinocytosis also confirmed

that the cells did not die at pH 3.4 in a timescale of 7-24 hours. Error Bar=S.D; No. of independent experimental replicates=3; *:$p \leq 0.05$, **: $p \leq 0.01$, ***:$p \leq 0.001$.

**S3 Fig: Autophagy and Senescence marker analysis at different pHe in LN229 glioblastoma cells.**

**A)** Beclin1 and **B)** LC3, two major autophagy associated proteins, did not show any consistent trendlines in cells exposed to different pHe. Beclin1 was reduced at very low pH whereas LC3 expression was enhanced. The mixed trendlines suggest that overall autophagy may be induced at all pH ranges and may assist in the survival of tumor cells. **C)** Shows that when senescence is induced by $H_2O_2$ treatment (positive control), there was a high cytoplasmic accumulation of beta-galactosidase enzymatic product (bluish-green in color). However, when tumor cells were exposed to various pH ranges, active enzyme localization was detected either on the plasma membrane or in acid organelles but such localizations are associated with the non-senescent condition. Error Bar=S.D; No. of independent experimental replicates=3; *:$p \leq 0.05$, **: $p \leq 0.01$, ***:$p \leq 0.001$.

**S4 Fig: Comparative Staining and Intensity analysis of LAMP2 and HMGCR in glioma zones of the same patient tissue.**

Necrotic Zone (pH <5.5-3.4), Pseudo-palisading cells layer (pH approx. 5.5 or less) and Cellular Tumor Zone (pH <7.0-6.2) from the same glioma tissue were compared for the expression of LAMP2 (an acidosis marker) and HMGCR (a rate limiting cholesterol synthesizing enzyme). Tabulated results showed that both proteins were highly expressed in necrotic and pseudo-palisading areas which are associated with very low pH. Moderate expression was also observed in Cellular Tumor Zones. The patient data was extracted from Human Protein Cancer Atlas (http://www.proteinatlas.org/).

**S5 Fig: Comparative Staining and Intensity analysis of LAMP2 and SREBF2 in glioma zones of the same patient tissue.**

Necrotic Zone (pH <5.5-3.4), Pseudo-palisading cells layer (pH approx. 5.5 or less) and Tumor Core (pH <7.0-6.2) from the same glioma tissue were compared with respect to the expression of LAMP2 (an acidosis marker) and SREBF2 (a transcription factor that

upregulates the synthesis of HMGCR, a rate-limiting enzyme in cholesterol synthesis). **A), B)** Results showed that both LAMP2 and SREBF2 were highly expressed in necrotic and pseudo-palisading zones (which are associated with very low pH). Moderate expression was detected in Cellular Tumor regions. The patient data was extracted from Human Protein Cancer Atlas (http://www.proteinatlas.org/).

**S6 Fig: Nystatin mediated surface cholesterol detection and quantitation**

Nystatin dye binds to cholesterol, hence enables its surface detection and quantitation. **A)** Shows fire LUT converted images of the nystatin dye fluorescent signal; this enabled us to quantitate surface cholesterol at different pH ranges. The experiment was performed on live cells which were fixed post dye incubation and the signal was captured by confocal microscopy (*please see supplementary methods and materials for details on the experimental setup*). **B)** Shows images of nystatin dye signal when the dye was incubated at room temperature on the surface of cells post-fixation. This enabled comparison of surface cholesterol profiles at low and room temperature. The overall trendlines of nystatin staining were found to be similar at the compared temperatures. Image analysis was done on over 200 cells in each condition using Fiji image processing software. Calibration bar for LUT converted images is shown at the bottom left of the figure. **C)** To quantitate surface cholesterol, nystatin dye signal was also measured with spectrofluorimetry-based method. Results obtained by both image and spectrofluorimetry based measurements corroborated well with each other. Results showed that with the decrease in pH, there was a significant rise in the surface cholesterol levels. All independent experiments had 3 technical replicates. Error bars are indicative of average standard deviations (S.D.) obtained across three independent experimental sets. The p values were obtained through Bonferroni's t-test between pH 7.4 units -veCD condition and other pH ranges in –veCD and +veCD conditions. p-values were represented with one star, 2 stars or 3 stars to denote $p \leq 0.05$, 0.01, 0.001 respectively.

**S7 Fig: Staining, Intensity and Localization analysis of OCT4 in different glioma zones**

Nuclear localization of Oct4 indicates proliferative and stem like properties of expressing cells. **A)** and **B)** Shows that necrotic and pseudo-palisading zone cells were low in nuclear and high in cytoplasmic expression for Oct4. **C), D)** Cellular Tumor Zone showed medium-

high nuclear and medium cytoplasmic expression of Oct4. The patient data was extracted from Human Protein Cancer Atlas (http://www.proteinatlas.org/).

**S8 Fig: Staining and Intensity analysis of GFAP in different glioma zones**

GFAP is a cytoplasmic marker of astrocytes and is also identified as a membrane-associated cytosketetal protein. It is highly expressed in matured/differentiated astrocytes. **A)**, **B)**, **C)**, **D)** and **E)** Shows that GFAP was strongly expressed in high-grade glioma and was found to be enriched in necrotic and peri-necrotic zones in comparison to cellular tumor zone (see panel **F)**. Note that necrotic zone cells have a particularly high surface expression of GFAP. The patient data was extracted from Human Protein Cancer Atlas (http://www.proteinatlas.org/).

**S9 Fig: Extracellular pH impacts cytoskeleton associated remodeling in a cholesterol-sensitive manner**

Rhodamine phalloidin binds to F-actin and identifies cortical, lamellipodial and stress fiber associated actin. α-actinin is a major F-actin associated protein and enable cell motility. **A)** Shows representative images of F-actin and α-actinin co-immunostained tumor cells exposed to various pHs. α-actinin was observed to penetrate well into the cortical actin areas in tumor cells exposed to physiological and low pHs (shown with white arrowheads). The actin stress fibers were diminished and severed in very low pH condition and showed prominent delocalization of α-actinin with cortical actin. **B)** Co-dynamics of surface beta-1 integrins and underlying alpha tubulins (palmitoylated) influence migration competencies. Cellular levels of alpha-tubulin along with respective SD's are indicated in each panel. Co-localization of beta-1 integrin and alpha-tubulin on the surface is indicated by Pearson's coefficient (R-value) and also highlighted with white arrows. Tumor cells at low pH in different conditions (pH 6.2 +veCD; pH 3.4 -veCD and +veCD) appeared to be supported by alpha tubulins although integrin expression was majorly reduced. This might be responsible for the migratory potential of tumor cells at low pH. **C)** Rac1, a small GTPase showed sub-membrane (white arrowheads) to nuclear localization in various pH microenvironments. High sub-membrane localization is associated with better migration competencies. This localization was associated with tumor cells at pH 7.4 in both basal and high cholesterol conditions. Error Bar=S.D; No. of independent experimental replicates=3; *:$p \leq 0.05$, **: $p \leq 0.01$, ***:$p \leq 0.001$.

**S10 Fig: Diagrammatic representation of GM3 Tilt angles.**

GM3 glycan tilt angle (Ɵ) denotes the extent of glycan bending to the membrane normal from the axis of the fatty acyl chains. It ranges from 0-90$^{o}$. Torsion angles (φ) allow rotation of glycans on the acyl chain axis. It ranges from 0-360$^{o}$.We did not find any significant difference in the torsion angles in our pH simulation studies; hence it was not further discussed in the main results.

**S11 Fig: Surface GM3 and cholesterol organization in different grades of glioblastoma**

Grade II gliomas are much less aggressive than Grade IV gliomas. **A)** Shows necrotic zones of Grade II gliomas which were enriched in GM3 and cholesterol expressions. **B)**, **C)** However, Grade II gliomas in non-necrotic niches showed lower levels of GM3 and cholesterol. Also, far less GM3 surface clustering was observed in Grade II gliomas in comparison to grade IV gliomas, as shown in D). **D)** Shows Pediatric grade IV GBM niches with 'high levels' of surface GM3 and cholesterol which was akin to observations made in adult GBM. Pediatric GBMs (Grade IV) also showed varying degrees of surface clustering with patterns that matched observations in *in vitro* acid stress studies. **E)** Shows IHC scoring and co-localization coeff. of GM3 and cholesterol in Grade II and IV glioma samples. The data was acquired by processing US Biomax Inc. GBM tissue array (with 63 distinct patient samples).

**S12 Fig: Surface profile of GM3 and other gangliosides in LN229 glioblastoma tumor cells.**

**A)** Shows LN229 glioblastoma cell line exposed to various pHs in -veCD-veHQ, +veCD and +veHQ conditions.Cells were fixed with 1.5% PFA at room temperature and then probed for surface GM3 clusters. Results show that the GM3 clustering was comparable to the experiment that was done at low temperature (live cells, on ice). **B)** Cells were treated with 0.5% saponin to disrupt lipid rafts (cholesterol and sphingolipid enriched domains). Results show that the GM3 surface clustering was dramatically reduced at all pHs upon saponin treatment, supporting the observations that cholesterol may stabilize GM3 surface ligations. **C)** GM3 precursor, lactosylceramide failed to show differential surface clustering at different pHs except in membrane protrusions. **D)** The tumor enriched alpha 2,3 polysialic acid

ganglioside, SSEA4, too failed to show surface clustering in response to different concentrations of protons. Hence, GM3 clustering in response to various pH was very specific and not a general function of gangliosides. The experiment was repeated 3 times.

**S13 Fig: Analysis of GM3 and Lactosylceramide surface levels at different pH upon sialidase treatment.**

**A)** and **B)** Shows that GM3 undergoes minimal lactonization in low pH conditions: Tumor surface GM3 signal was quantified in basal (-veCD) and high cholesterol (+veCD) conditions at various pH ranges. The neuraminidase treatments were performed on cells that were either kept on ice (cold treatment to prevent endocytosis of lipids concerned) or at room temperature. The GM3 signal detection was performed post fixation. Since GM3 lactonized forms are documented to be neuraminidase resistant, the major loss of GM3 signal from the surface, upon digestion of the neuraminic/sialic acid moiety, indicated that the observed GM3 clusters (as described in Fig10) were predominantly due to GM3-GM3 ligations and not due to the formation of GM3 lactones. Cleavage of the sialic acid moiety from GM3 converts this lipid into its precursor form known as lactosylceramide. **C)** and **D)** Shows an increase in surface lactosylceramide signal upon neuraminidase digestion with concomitant loss of GM3 signal in the same conditions (see A, B) which indicates that the extent of GM3 lactones formed on the cell surface in low pH condition contributed only minimally to GM3 clustered pattern. More specifically when neuraminidase treatment was performed at cold temperature, the estimated % of GM3 clusters due to lactonization was as follows: 16.7% at pH 6.2 and 23.2 % at pH 3.4 in -veCD condition; 17.1 % at pH 6.2 and 28.4% at pH 3.4 in +veCD condition. However, when neuraminidase treatment was performed at RT and GM3 signal detection was then followed, the estimated % of GM3 clusters due to lactonization was found to be 4.4% at pH 6.2 and 5.4 % at pH 3.4 in -veCD condition; 0.2% at pH 6.2 and 0.6 % at pH 3.4 in +veCD condition. The % of GM3 signal at respective pHs was calculated over the untreated controls signals at the same pH. Hence, GM3 clustering effects were majorly due to GM3-GM3 ligations. The resulting signal of both GM3 and lactosylceramide in the cells that were digested with neuraminidase at RT were lower in comparison to the cells that were kept in cold due to the enhanced endocytosis of the lipids at RT or due to higher activity of neuraminidase. Error Bar=S.D; No. of independent experimental replicates=3.

**S14 Fig: IgM control antibody fails to show GM3 and GFAP supraclustering in LN229 glioblastoma cells.**

**A), B), C)** and **D)** pH graded LN229 tumor cell microenvironments in the unperturbed cholesterol and cholesterol high conditions were incubated with human IgM antibody instead of anti-GM3 IgM antibody and subsequently probed with Alexa 488 secondary antibody. In this experiment no GM3 clusters were observed. The cells showed variable degrees of stress fibers and no severing of stress fibers was noticed, hence the clustering effect of anti-GM3 antibody was specific and originates from extensive clustering of GM3 in the oncogenic pH microenvironments.

**S15 Fig: Analysis of Cyclophilin A in glioblastoma patient samples.**

**A)** Shows levels of cyclophilin A (CyPA) in various tumors and also compares it with respective normal tissue. Note that glioma expresses high levels of cyclophilin A however normal tissue had negligible expression. **B)** Shows that normal adult brain tissue has a negligible expression of CyPA. **C)** and **D)** Glioma shows high nuclear, cytoplasmic, membranous expression of CyPA and also its secreted form was detected in the extracellular space. The patient data was extracted from Human Protein Cancer Atlas (http://www.proteinatlas.org/).

**S16 Fig: Release of Cyclophilin A from glioblastoma cell line (LN229) in different pH conditions.**

**A)**, **B)** and **C)** Shows that intracellular cyclophilinA was very high at pH 3.4 but failed to be released extracellularly, whereas, a high level of vesicular release was observed in the physiological (7.4) and much more at the low (6.2) pH condition. Error Bar=S.D; No. of independent experimental replicates=3; *:$p \leq 0.05$, **: $p \leq 0.01$, ***:$p \leq 0.001$.

**S17 Fig: Cyclophilin A release inhibitor induces glioblastoma tumor cell anoikis *via* GM3 and GFAP supra-clustering.**

**A)**, **B)**, **C)** and **D** Insets in DIC images show that upon CyPA inhibitor treatment, cyclophilin A vesicular release was inhibited, and it was trapped inside the cells. DIC images represent

that within 30-33 hours of CyPA treatment (within 3-6 hours of 2$^{nd}$ shot) massive cellular blebbing, cell rounding and loss of anchorage occurred. Several tumor cells were levitated and underwent secondary necrosis within this timeframe. **A)**, **B)**, **C)** and **D)** CyPA inhibitor treated LN229 tumor cells showed massive GM3 clustered patterns over the untreated cells in oncogenic pH microenvironments in both unperturbed cholesterol and cholesterol high conditions. The patterns of GM3 clusters colocalized well with the intracellular membranous GFAP protein clustering. Note that the Pearson's colocalization coefficient [R-values] between GM3 and GFAP in cyclophilin A inhibitor treated conditions was >0.7±S.D, hence was highly significant. The experiment was repeated 3 times.  F-actin was visualized *via* rhodamine conjugated phalloidin which showed disruption of actin stress fibers in CyPA inhibitor *vs.* the control condition.

# SUPPLEMENTARY FIGURES S1-S17

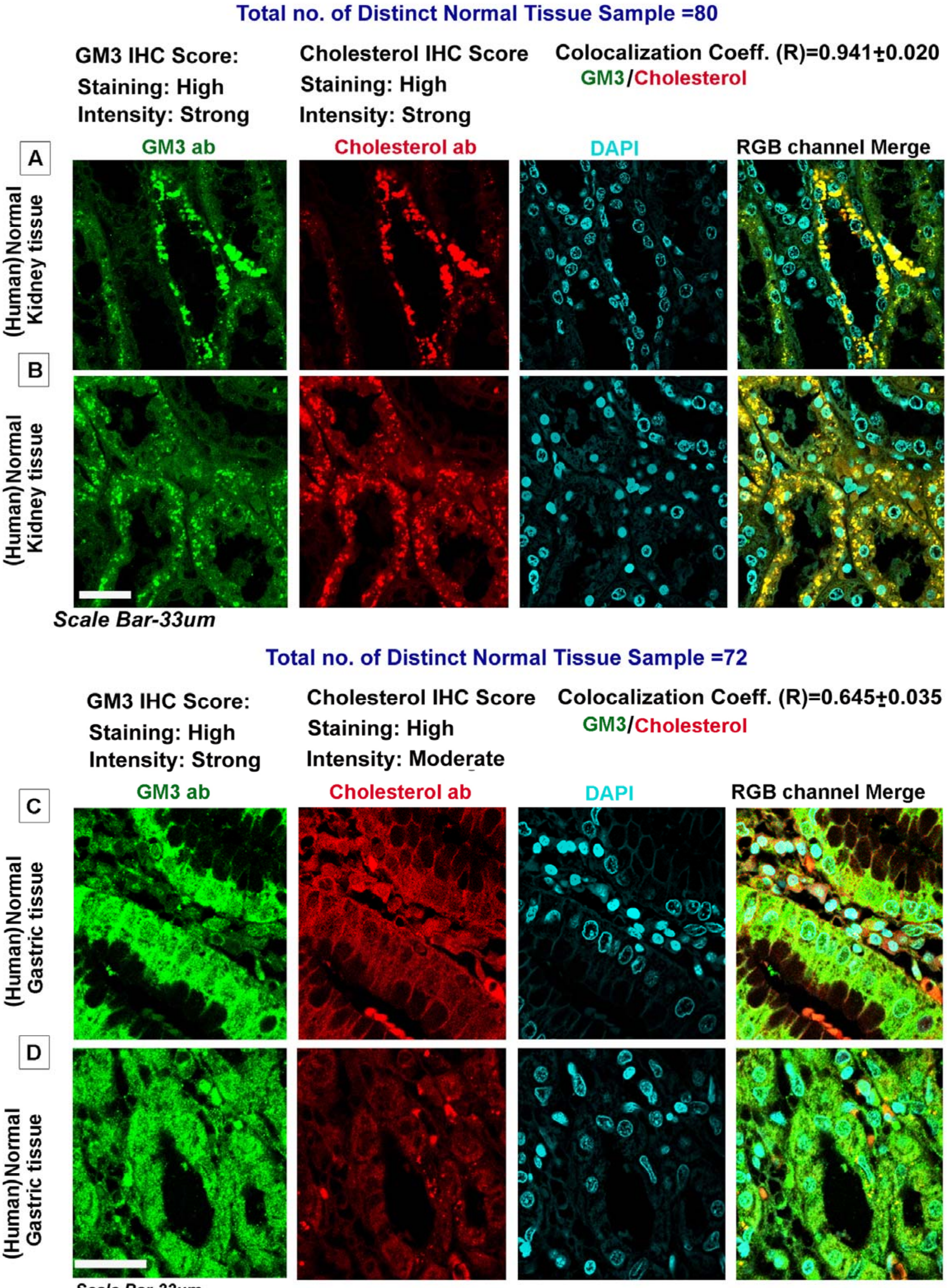
Total no. of Distinct Normal Tissue Sample =80
GM3 IHC Score:
Staining: High
Intensity: Strong
Cholesterol IHC Score
Staining: High
Intensity: Strong
Colocalization Coeff. (R)=0.941±0.020
GM3/Cholesterol
GM3 ab
Cholesterol ab
DAPI
RGB channel Merge
A
B
(Human)Normal Kidney tissue
(Human)Normal Kidney tissue
Scale Bar-33um
Total no. of Distinct Normal Tissue Sample =72
GM3 IHC Score:
Staining: High
Intensity: Strong
Cholesterol IHC Score
Staining: High
Intensity: Moderate
Colocalization Coeff. (R)=0.645±0.035
GM3/Cholesterol
GM3 ab
Cholesterol ab
DAPI
RGB channel Merge
C
D
(Human)Normal Gastric tissue
(Human)Normal Gastric tissue
Scale Bar-33um

S1 Figure

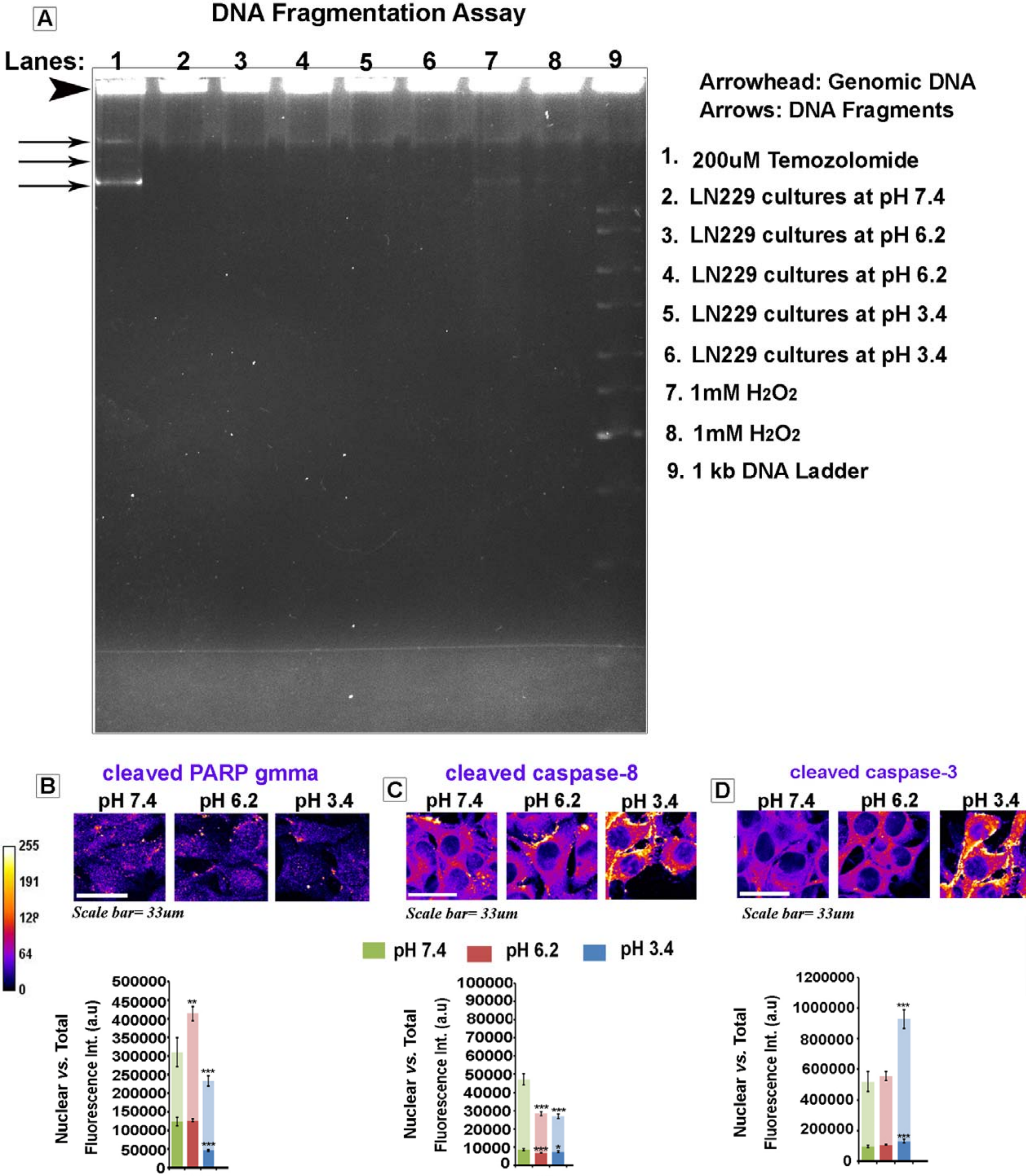

A
DNA Fragmentation Assay
Lanes: 1 2 3 4 5 6 7 8 9
Arrowhead: Genomic DNA
Arrows: DNA Fragments
1. 200uM Temozolomide
2. LN229 cultures at pH 7.4
3. LN229 cultures at pH 6.2
4. LN229 cultures at pH 6.2
5. LN229 cultures at pH 3.4
6. LN229 cultures at pH 3.4
7. 1mM H2O2
8. 1mM H2O2
9. 1 kb DNA Ladder
B
cleaved PARP gmma
C
cleaved caspase-8
D
cleaved caspase-3
pH 7.4
pH 6.2
pH 3.4
Scale bar= 33um
255
191
128
64
0
pH 7.4
pH 6.2
pH 3.4
Nuclear vs. Total
Fluorescence Int. (a.u)


S2 Figure

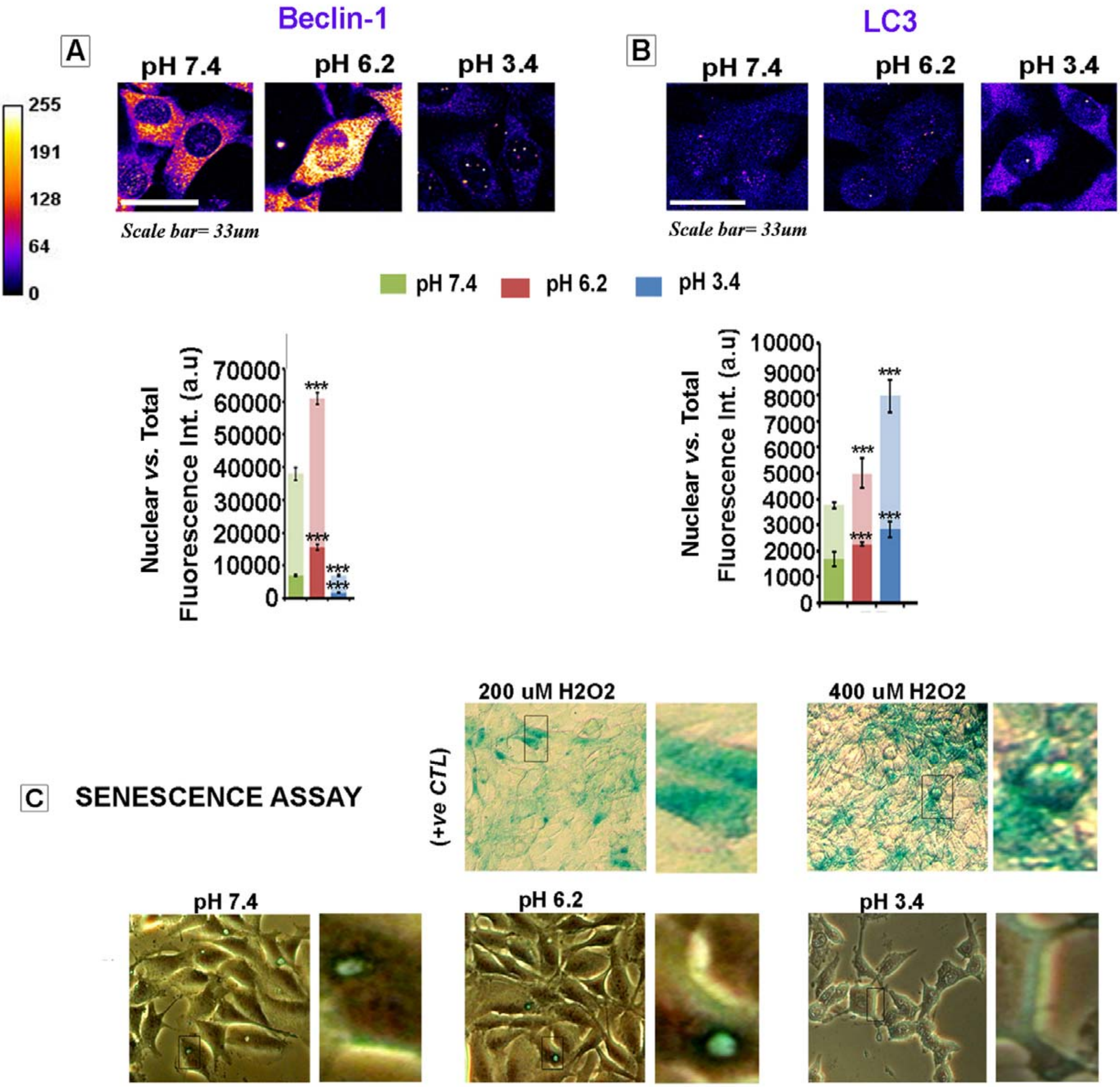

Beclin-1
LC3
A
B
pH 7.4
pH 6.2
pH 3.4
255
191
128
64
0
Scale bar= 33um
Scale bar= 33um
pH 7.4
pH 6.2
pH 3.4
Nuclear vs. Total Fluorescence Int. (a.u)
70000
60000
50000
40000
30000
20000
10000
0
***
10000
9000
8000
7000
6000
5000
4000
3000
2000
1000
C
SENESCENCE ASSAY
200 uM H2O2
400 uM H2O2
(+ve CTL)
pH 7.4
pH 6.2
pH 3.4


S3 Figure

## IHC Scoring of LAMP2 and HMGCR in the same glioma tissue

| S No. | Patient ID | Grade | Protein | Necrotic Zone | | Pseudopalisading Zone | | Cellular Tumor Zone | |
|---|---|---|---|---|---|---|---|---|---|
| | | | | Staining | Intensity | Staining | Intensity | Staining | Intensity |
| 1 | 1587 | High | LAMP2 | — | — | — | — | Medium | Moderate |
| | | | HMGCR | — | — | — | — | Low | Moderate |
| 2 | 1537 | High | LAMP2 | High | Strong | High | Strong | Medium | Moderate |
| | | | HMGCR | Moderate | Strong | High | Strong | Medium | Moderate |
| 3 | 2726 | High | LAMP2 | High | Strong | High | Strong | — | — |
| | | | HMGCR | High | Strong | Medium | Moderate | Medium | Moderate |
| 4 | 2750 | High | LAMP2 | High | Strong | High | Strong | — | — |
| | | | HMGCR | High | Strong | High | Strong | — | — |
| 5 | 2529 | Low | LAMP2 | — | — | Medium | Moderate | Medium | Moderate |
| | | | HMGCR | — | — | Medium | Moderate | Medium | Moderate |

**S4 Figure**

A

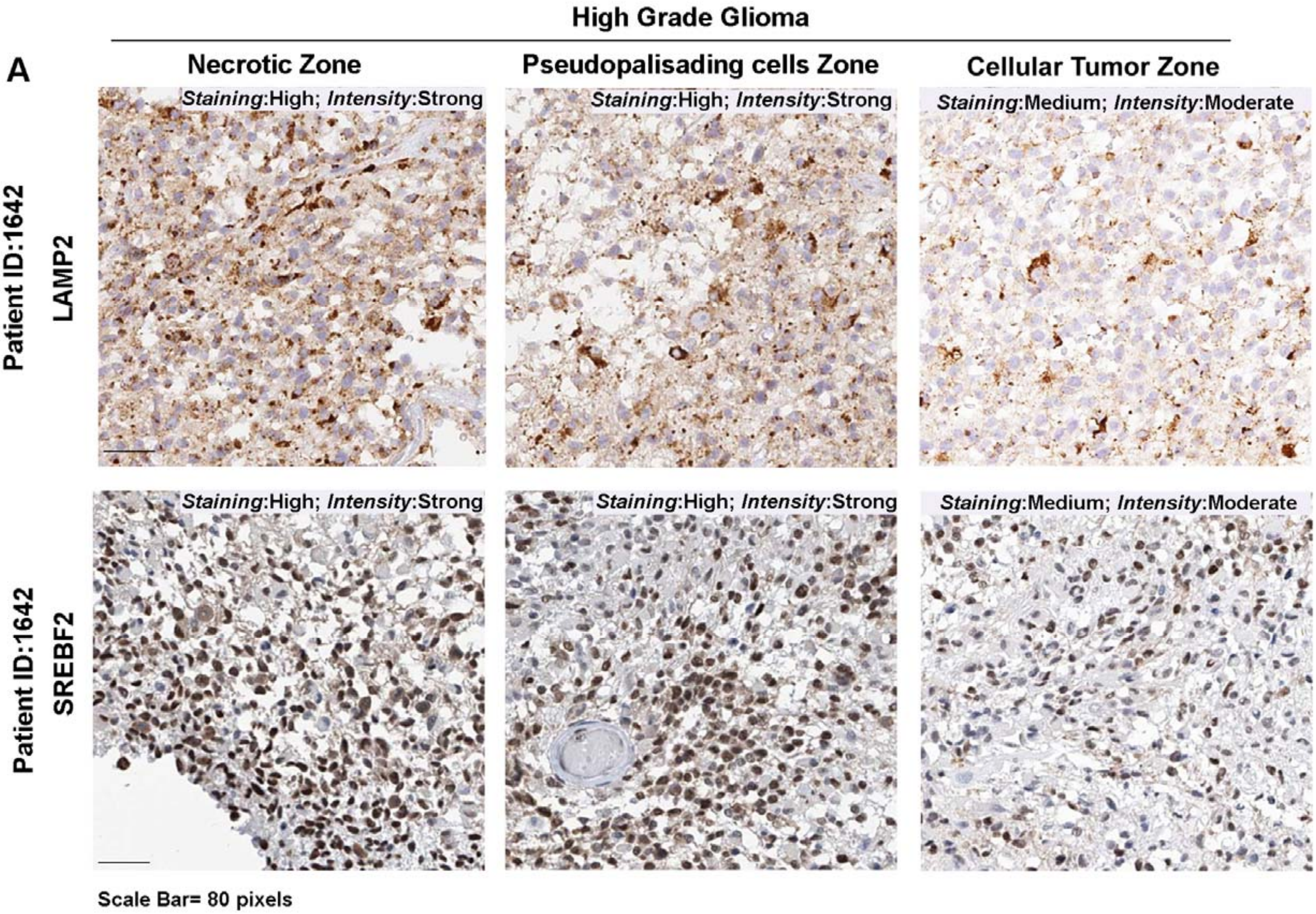


B

## IHC Scoring of LAMP2 and SREBF2 in the same patient tissue

| S No. | Patient ID | Grade | Protein | Necrotic Zone | | Pseudopalisading Zone | | Cellular Tumor Zone | |
|---|---|---|---|---|---|---|---|---|---|
| | | | | Staining | Intensity | Staining | Intensity | Staining | Intensity |
| 1 | 3326 | High | LAMP2 | Medium | Strong | High | Strong | Medium | Moderate |
| | | | SREBF2 | Medium | Strong | High | Strong | Medium | Moderate |
| 2 | 1645 | High | LAMP2 | Medium | Moderate | Medium | Strong | Medium | Moderate |
| | | | SREBF2 | Medium | Strong | Medium | Strong | Medium | Moderate |
| 3 | 1642 | High | LAMP2 | High | High | High | Strong | Medium | Moderate |
| | | | SREBF2 | High | Strong | High | Strong | Medium | Moderate |

S5 Figure

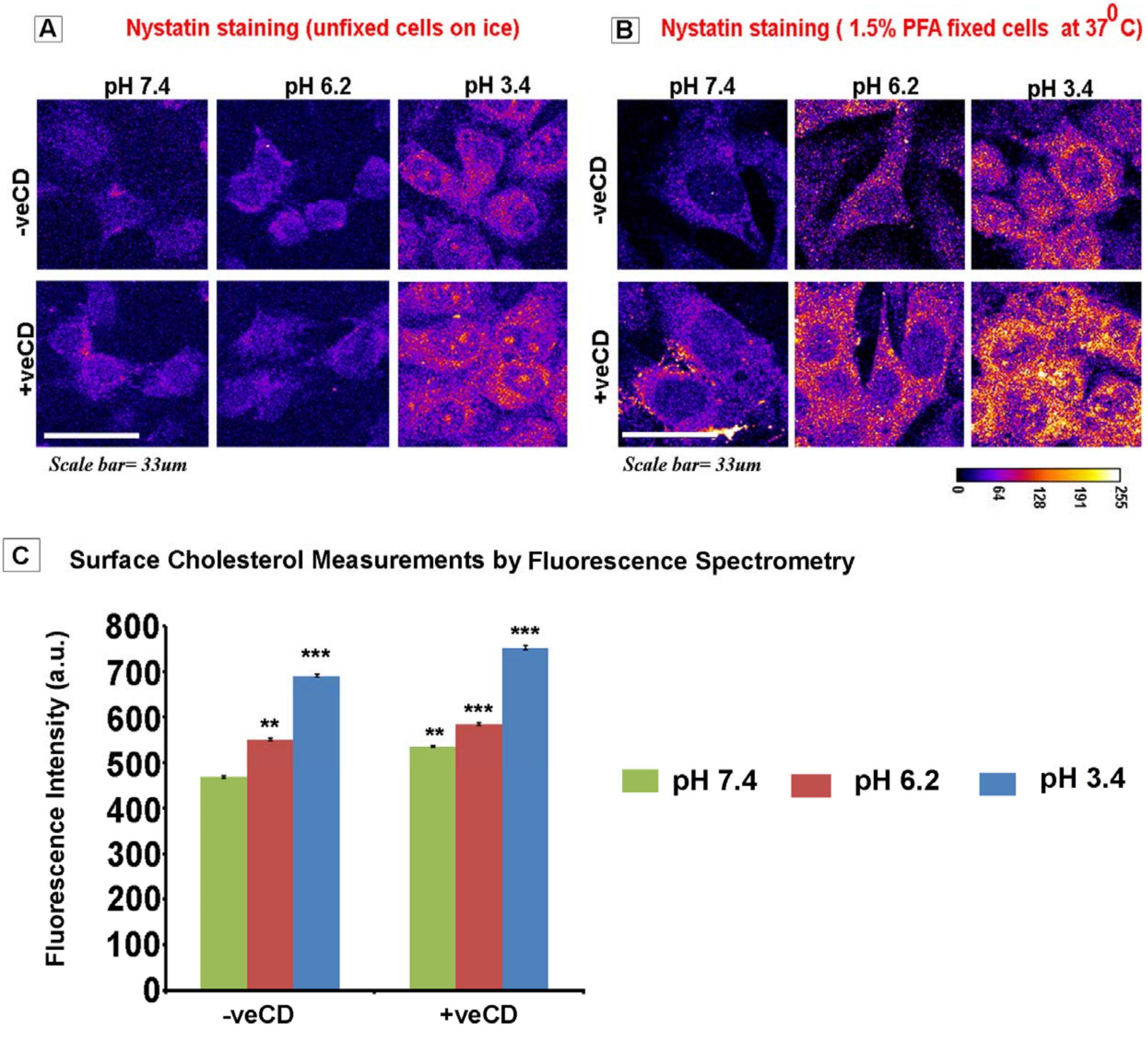
A
Nystatin staining (unfixed cells on ice)
B
Nystatin staining ( 1.5% PFA fixed cells at 37 0 C)
pH 7.4
pH 6.2
pH 3.4
-veCD
+veCD
Scale bar= 33um
Scale bar= 33um
0
64
128
191
255
C
Surface Cholesterol Measurements by Fluorescence Spectrometry
Fluorescence Intensity (a.u.)
800
700
600
500
400
300
200
100
0
**
***
**
***
***
-veCD
+veCD
pH 7.4
pH 6.2
pH 3.4

S6 Figure

## Oct4/POU5F1

**A** Necrotic and Pseudopalisading zone

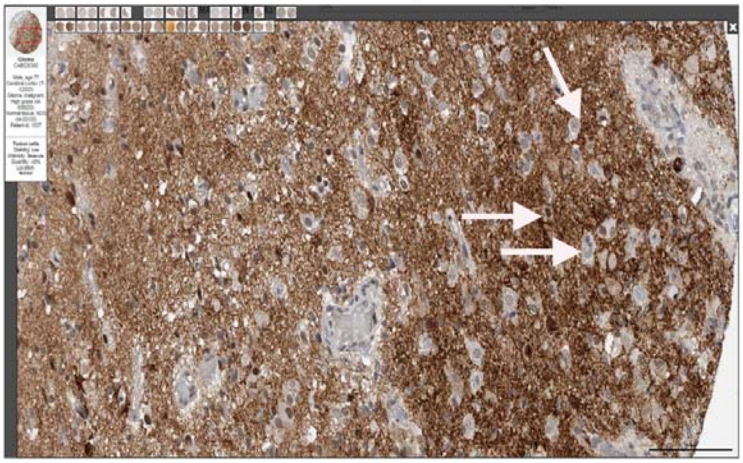

Staining:*High*;Intensity:***Strong***:Localization:***Cytoplasm***

**B** Necrotic and Pseudopalisading zone

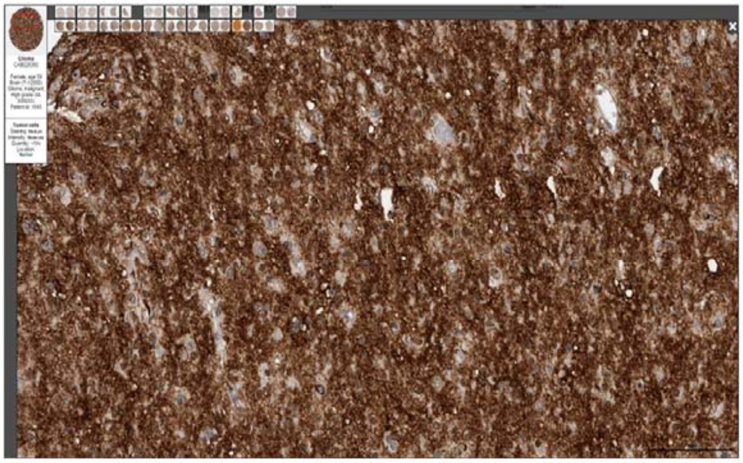

Staining:*High*;Intensity:***Strong***:Localization:***Cytoplasm***

**C** Cellular Tumor Zone

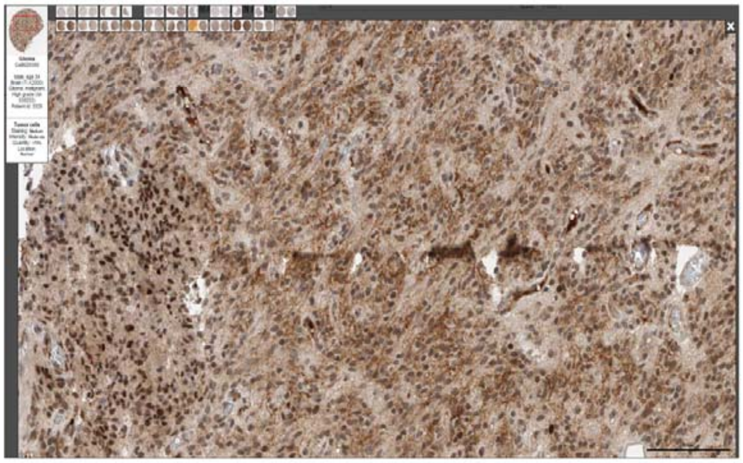

Staining:*Medium*;Intensity:***Strong***:Localization:***Cytoplasm /nuclear***

**D** Cellular Tumor Zone

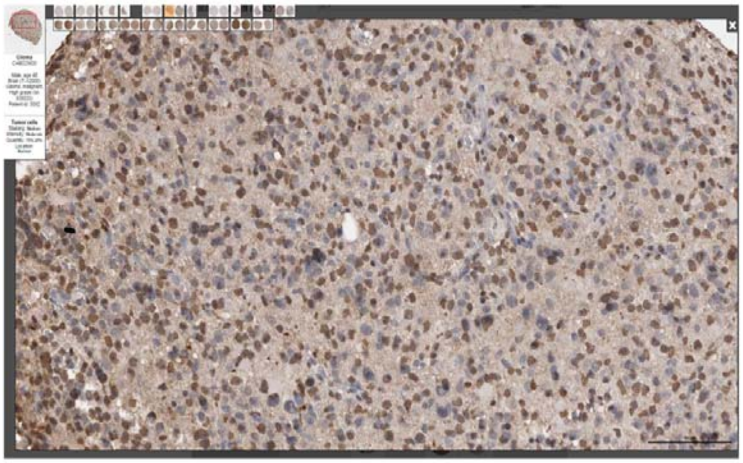

Staining:*Medium*;Intensity:***Strong***:Localization:***nuclear***

### IHC Score of OCT4 in different patients

| S No. | Grade | Necrotic Zone | | Pseudopalisading Zone | | Cellular Tumor Zone | |
|---|---|---|---|---|---|---|---|
| | | Staining | Intensity | Staining | Intensity | Staining | Intensity |
| 1 | High | High | Strong | High | Strong | Medium | Moderate |
| | | Patients=3; Local.:Cytoplasmic | | Patients=4; Local.:Cytoplasmic | | Patients=8; Local.:Cytoplasmic | |
| 2 | High | High | Strong | High | Strong | Medium | Moderate |
| | | Patients=2; Local.:Cyto./Nuclear | | Patients=1; Local.:Cyto./Nuclear | | Patients=1; Local.:Nuclear | |
| 3 | High | — | — | Medium | Moderate | Medium | Moderate |
| | | — | | Patients=1; Local.:Cyto./Nuclear | | Patients=1; Local.:Cyto./Nuclear | |
| 4 | High | — | — | — | — | High | Strong |
| | | — | | — | | Patients=1; Local.:Cyto./Nuclear | |

**S7 Figure**

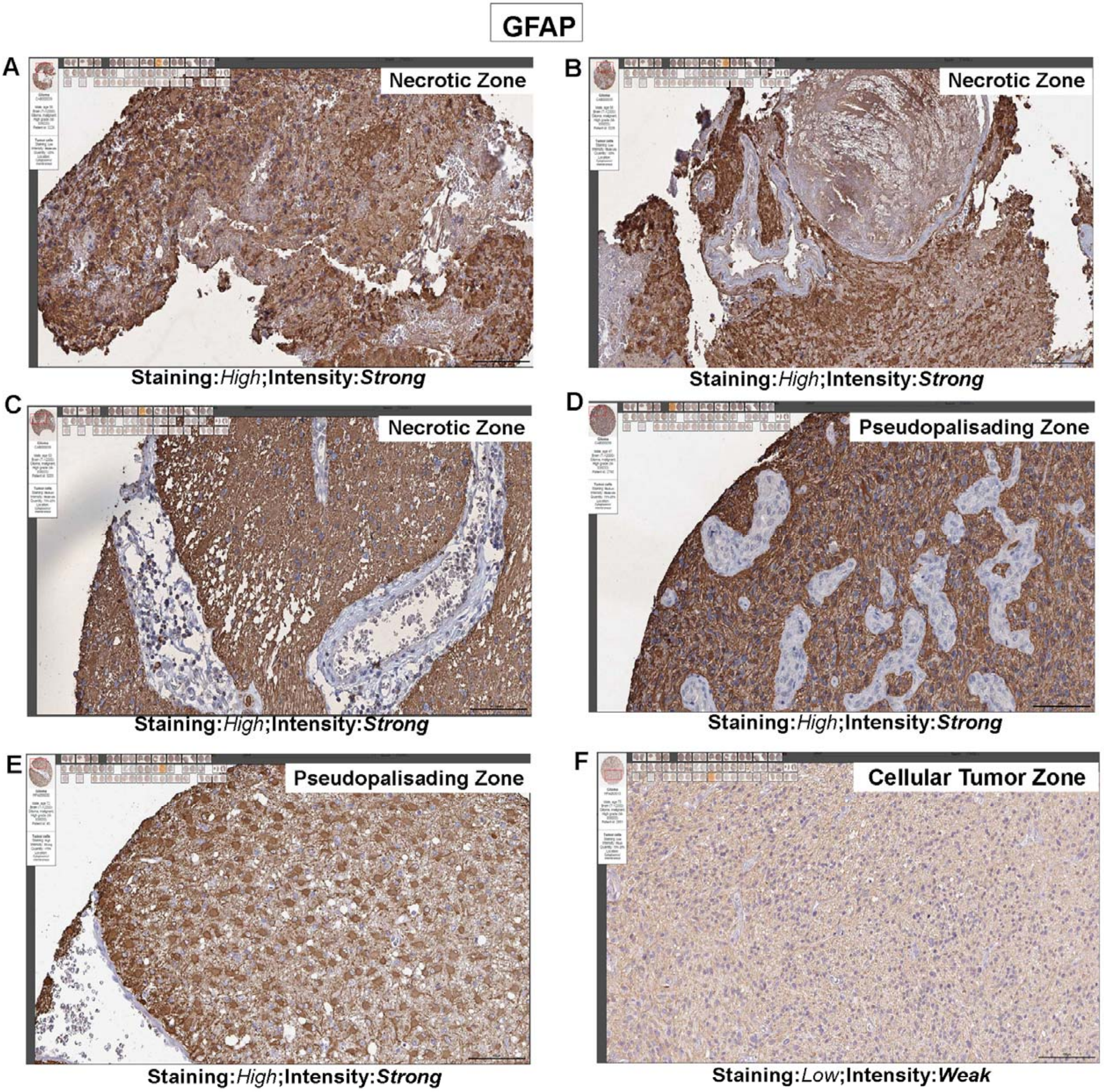


**IHC Score of GFAP in different patients**

| S No. | Grade | Necrotic Zone | | Pseudopalisading Zone | | Cellular Tumor Zone | |
|---|---|---|---|---|---|---|---|
| | High | Staining | Intensity | Staining | Intensity | Staining | Intensity |
| 1 | High | High | Strong | High | Strong | Low | Weak |
| | | Patients=5 | | Patients=5 | | Patients=2 | |
| 2 | High | — | — | — | — | Low | Moderate |
| | | — | | — | | Patients=1 | |

S8 Figure

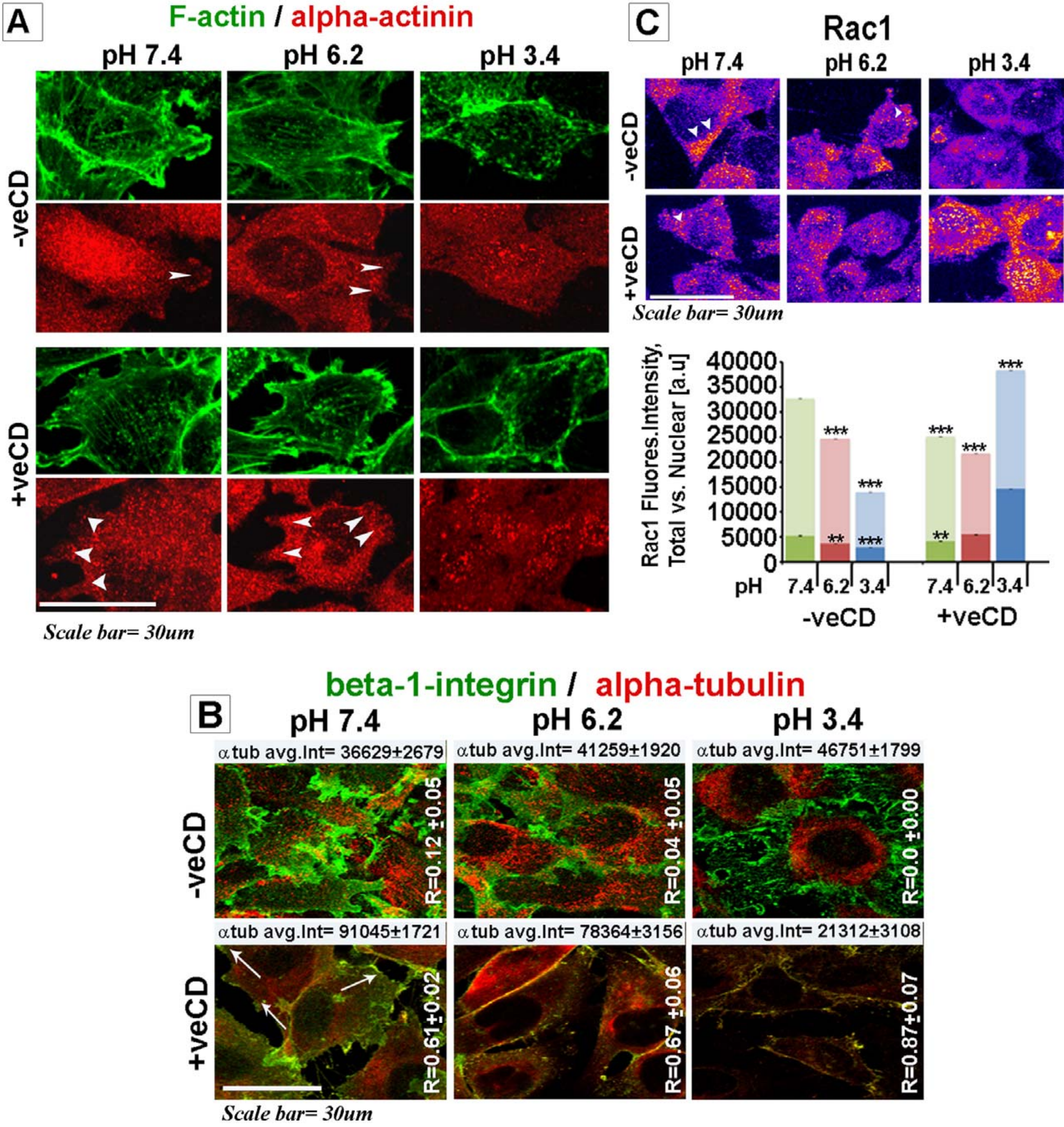
A
F-actin / alpha-actinin
pH 7.4
pH 6.2
pH 3.4
-veCD
+veCD
Scale bar= 30um
C
Rac1
pH 7.4
pH 6.2
pH 3.4
-veCD
+veCD
Scale bar= 30um
Rac1 Fluores.Intensity, Total vs. Nuclear [a.u]
40000
35000
30000
25000
20000
15000
10000
5000
0
***
**
pH
7.4
6.2
3.4
-veCD
+veCD
B
beta-1-integrin / alpha-tubulin
pH 7.4
pH 6.2
pH 3.4
αtub avg.Int= 36629±2679
αtub avg.Int= 41259±1920
αtub avg.Int= 46751±1799
R=0.12 ±0.05
R=0.04 ±0.05
R=0.0 ±0.00
αtub avg.Int= 91045±1721
αtub avg.Int= 78364±3156
αtub avg.Int= 21312±3108
R=0.61±0.02
R=0.67 ±0.06
R=0.87±0.07
-veCD
+veCD
Scale bar= 30um

S9 Figure

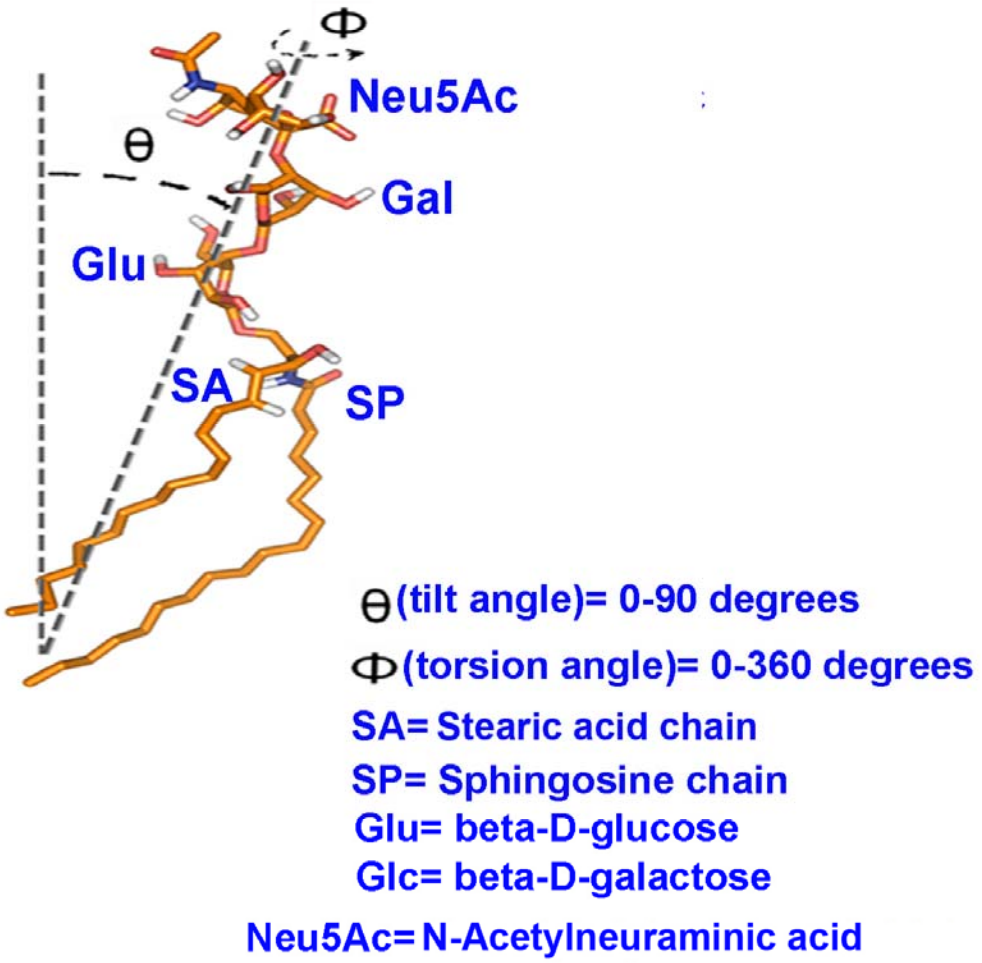
Φ
Neu5Ac
θ
Gal
Glu
SA
SP
θ(tilt angle)= 0-90 degrees
Φ(torsion angle)= 0-360 degrees
SA= Stearic acid chain
SP= Sphingosine chain
Glu= beta-D-glucose
Glc= beta-D-galactose
Neu5Ac= N-Acetylneuraminic acid

**S10 Figure**

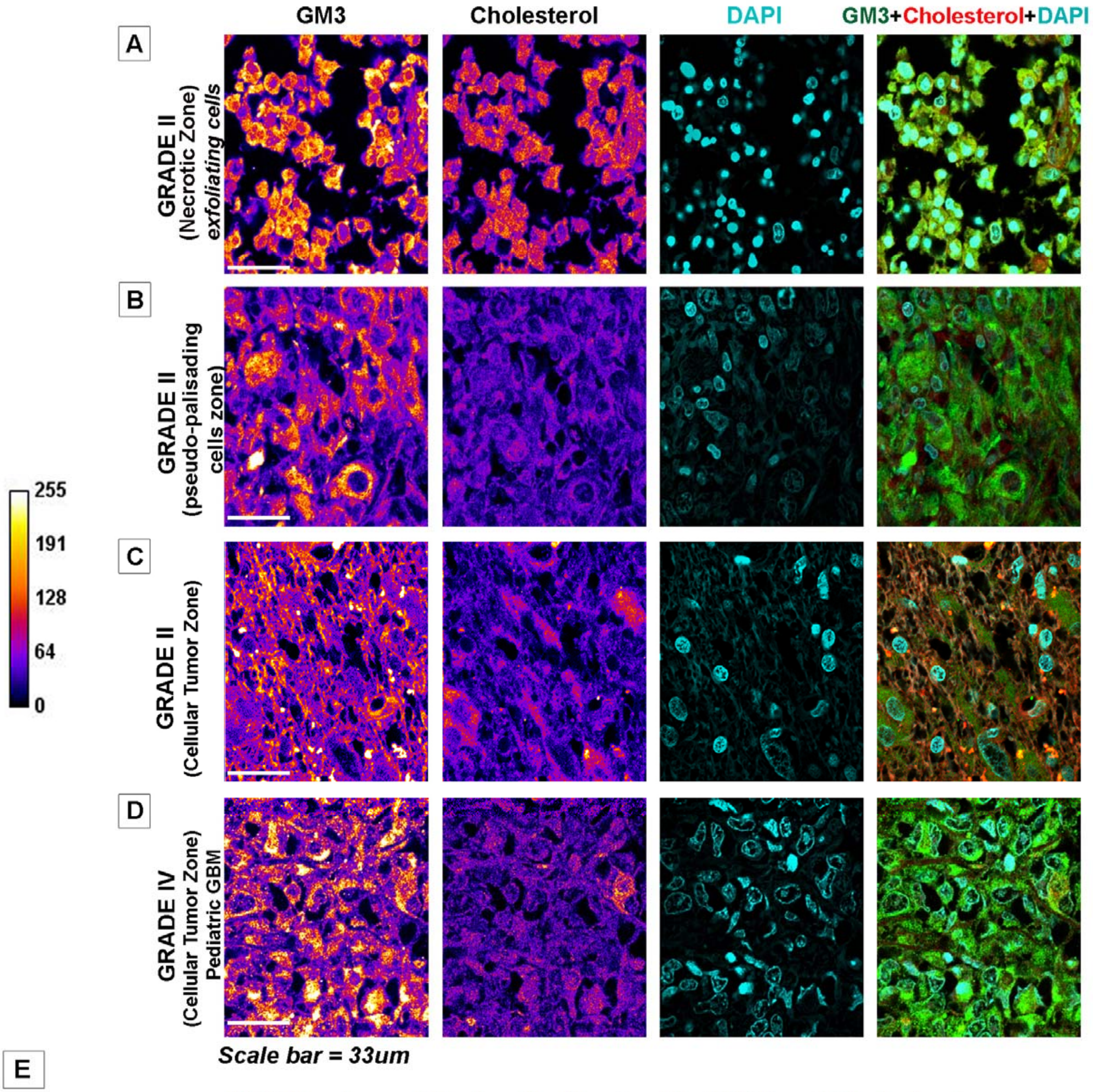


E

**IHC Scoring and Co-localization Coeff. of GM3 and Cholesterol**

| S.No | No. of Samples | Tumor Grade | GM3 | | Cholesterol | | Co-localization Coeff. of GM3/Cholesterol |
|---|---|---|---|---|---|---|---|
| | | | Staining | Intensity | Staining | Intensity | |
| 1 | 4 | II | High | Strong | Medium | Moderate | 0.0478±0.11 |
| 2 | 4 | IV Pediatric GBM | High | Strong | Medium | Moderate | 0.731±0.026 |

Note that each sample had 3 technical replicates: data obtained from 3 US Biomax Slides with samples from the same patients

S11 Figure

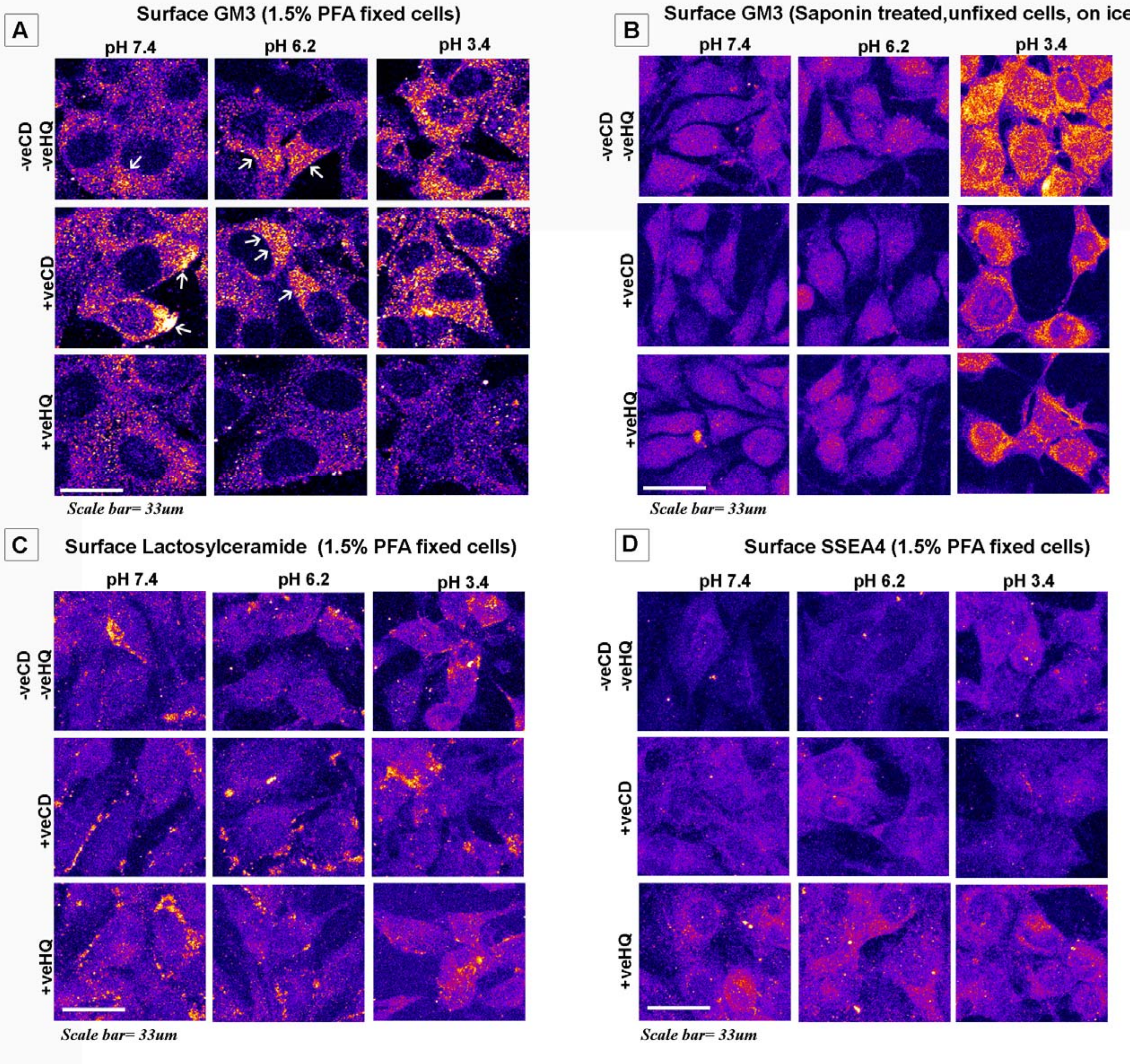

A
Surface GM3 (1.5% PFA fixed cells)
pH 7.4
pH 6.2
pH 3.4
-veCD -veHQ
+veCD
+veHQ
Scale bar= 33um
B
Surface GM3 (Saponin treated,unfixed cells, on ice
pH 7.4
pH 6.2
pH 3.4
-veCD -veHQ
+veCD
+veHQ
Scale bar= 33um
C
Surface Lactosylceramide (1.5% PFA fixed cells)
pH 7.4
pH 6.2
pH 3.4
-veCD -veHQ
+veCD
+veHQ
Scale bar= 33um
D
Surface SSEA4 (1.5% PFA fixed cells)
pH 7.4
pH 6.2
pH 3.4
-veCD -veHQ
+veCD
+veHQ
Scale bar= 33um


S12 Figure

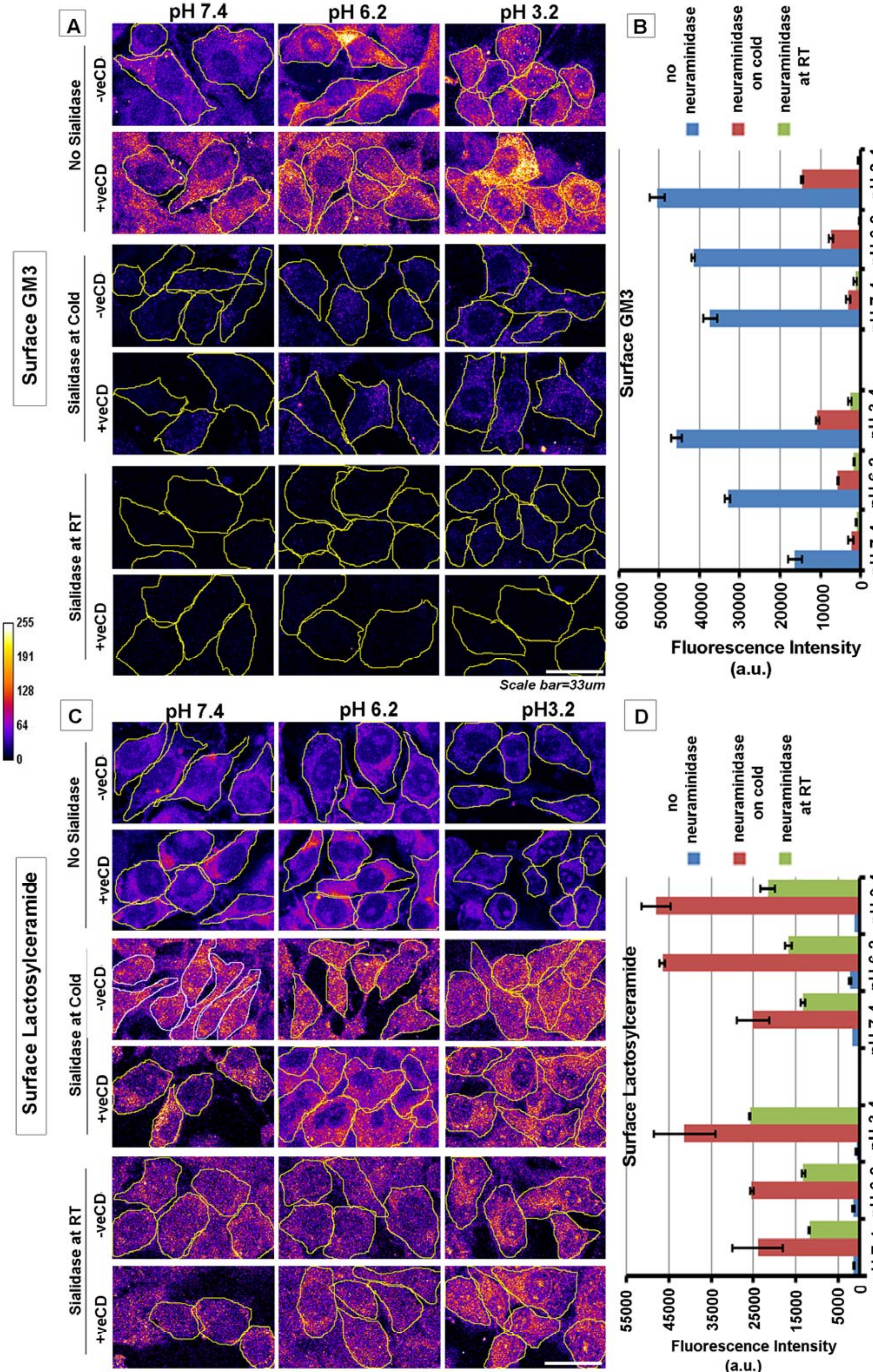
A
pH 7.4
pH 6.2
pH 3.2
Surface GM3
No Sialidase
Sialidase at Cold
Sialidase at RT
-veCD
+veCD
Scale bar=33um
B
no neuraminidase
neuraminidase on cold
neuraminidase at RT
Fluorescence Intensity (a.u.)
60000
50000
40000
30000
20000
10000
0
pH 7.4
pH 6.2
pH 3.4
(-veCD)
(+veCD)
255
191
128
64
0
C
pH 7.4
pH 6.2
pH3.2
Surface Lactosylceramide
D
55000
45000
35000
25000
15000
5000
0

S13 Figure

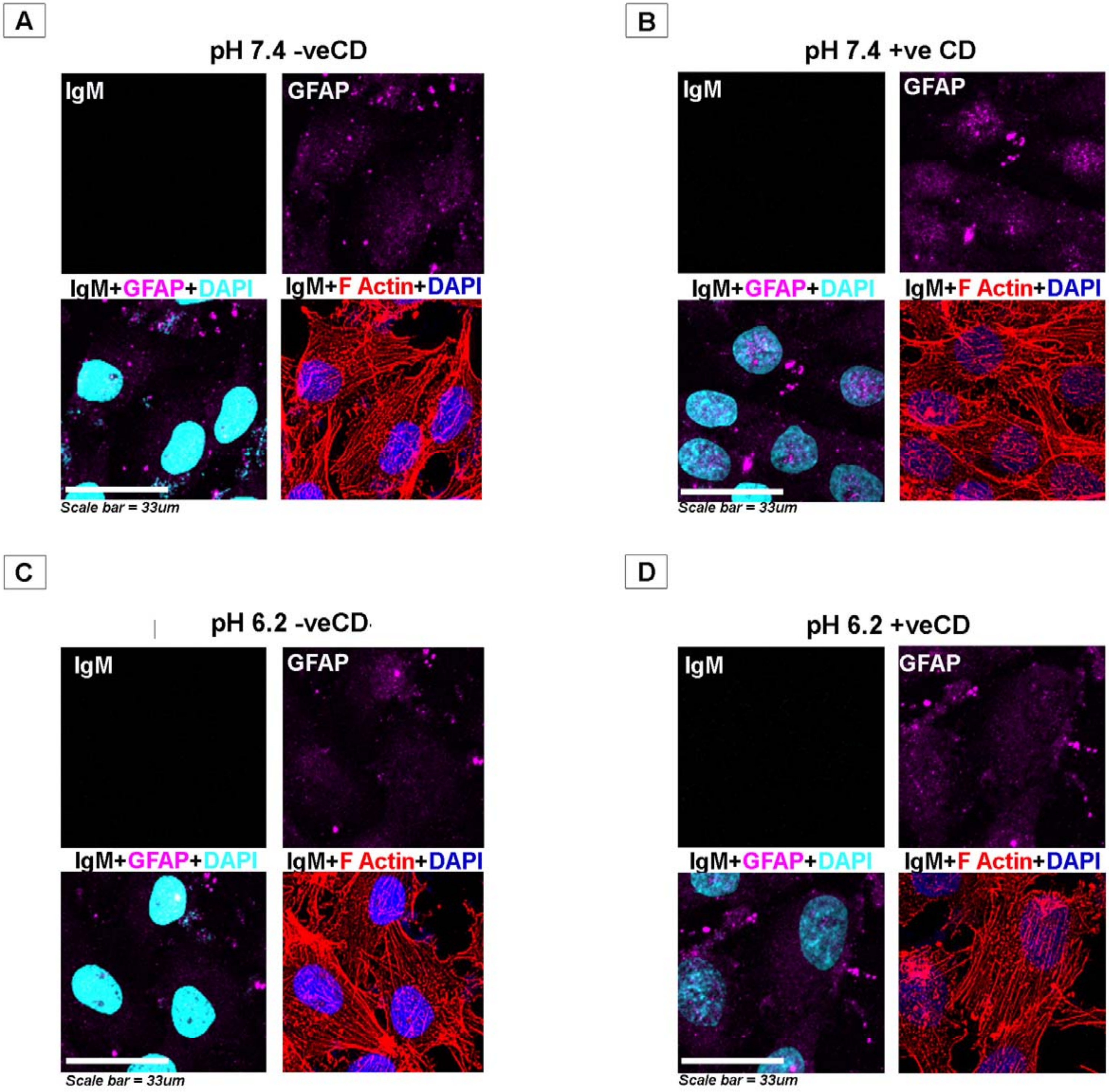
A
pH 7.4 -veCD
IgM
GFAP
IgM+GFAP+DAPI
IgM+F Actin+DAPI
Scale bar = 33um
B
pH 7.4 +ve CD
IgM
GFAP
IgM+GFAP+DAPI
IgM+F Actin+DAPI
Scale bar = 33um
C
pH 6.2 -veCD
IgM
GFAP
IgM+GFAP+DAPI
IgM+F Actin+DAPI
Scale bar = 33um
D
pH 6.2 +veCD
IgM
GFAP
IgM+GFAP+DAPI
IgM+F Actin+DAPI
Scale bar = 33um

**S14 Figure**

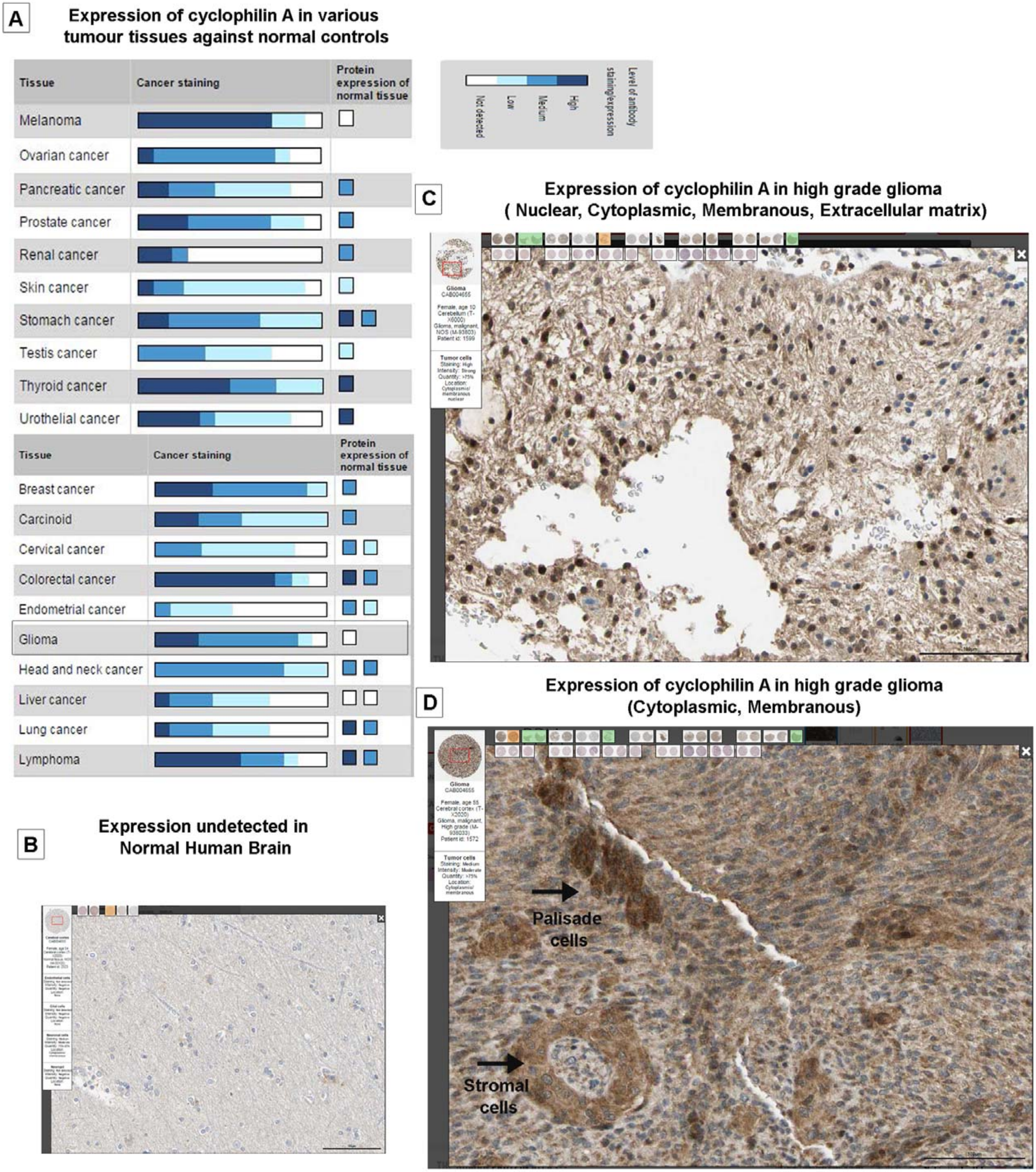
A
Expression of cyclophilin A in various tumour tissues against normal controls
Tissue
Cancer staining
Protein expression of normal tissue
Melanoma
Ovarian cancer
Pancreatic cancer
Prostate cancer
Renal cancer
Skin cancer
Stomach cancer
Testis cancer
Thyroid cancer
Urothelial cancer
Tissue
Cancer staining
Protein expression of normal tissue
Breast cancer
Carcinoid
Cervical cancer
Colorectal cancer
Endometrial cancer
Glioma
Head and neck cancer
Liver cancer
Lung cancer
Lymphoma
Level of antibody staining/expression
Not detected
Low
Medium
High
B
Expression undetected in Normal Human Brain
C
Expression of cyclophilin A in high grade glioma
( Nuclear, Cytoplasmic, Membranous, Extracellular matrix)
D
Expression of cyclophilin A in high grade glioma
(Cytoplasmic, Membranous)
Palisade cells
Stromal cells

S15 Figure

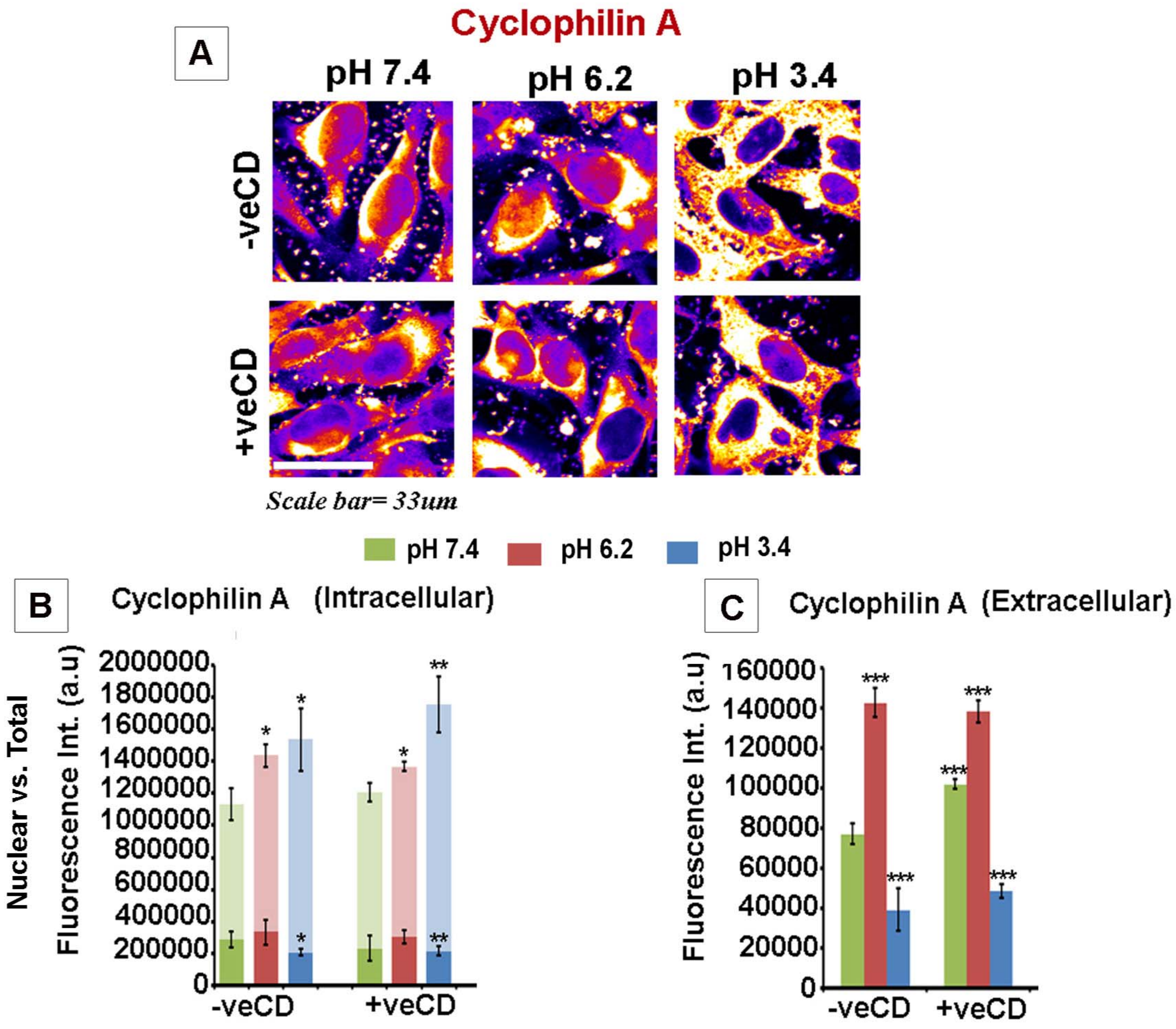

Cyclophilin A
A
pH 7.4
pH 6.2
pH 3.4
-veCD
+veCD
Scale bar= 33um
pH 7.4
pH 6.2
pH 3.4
B
Cyclophilin A (Intracellular)
Nuclear vs. Total
Fluorescence Int. (a.u)
2000000
1800000
1600000
1400000
1200000
1000000
800000
600000
400000
200000
0
-veCD
+veCD
C
Cyclophilin A (Extracellular)
Fluorescence Int. (a.u)
160000
140000
120000
100000
80000
60000
40000
20000
0
-veCD
+veCD


S16 Figure

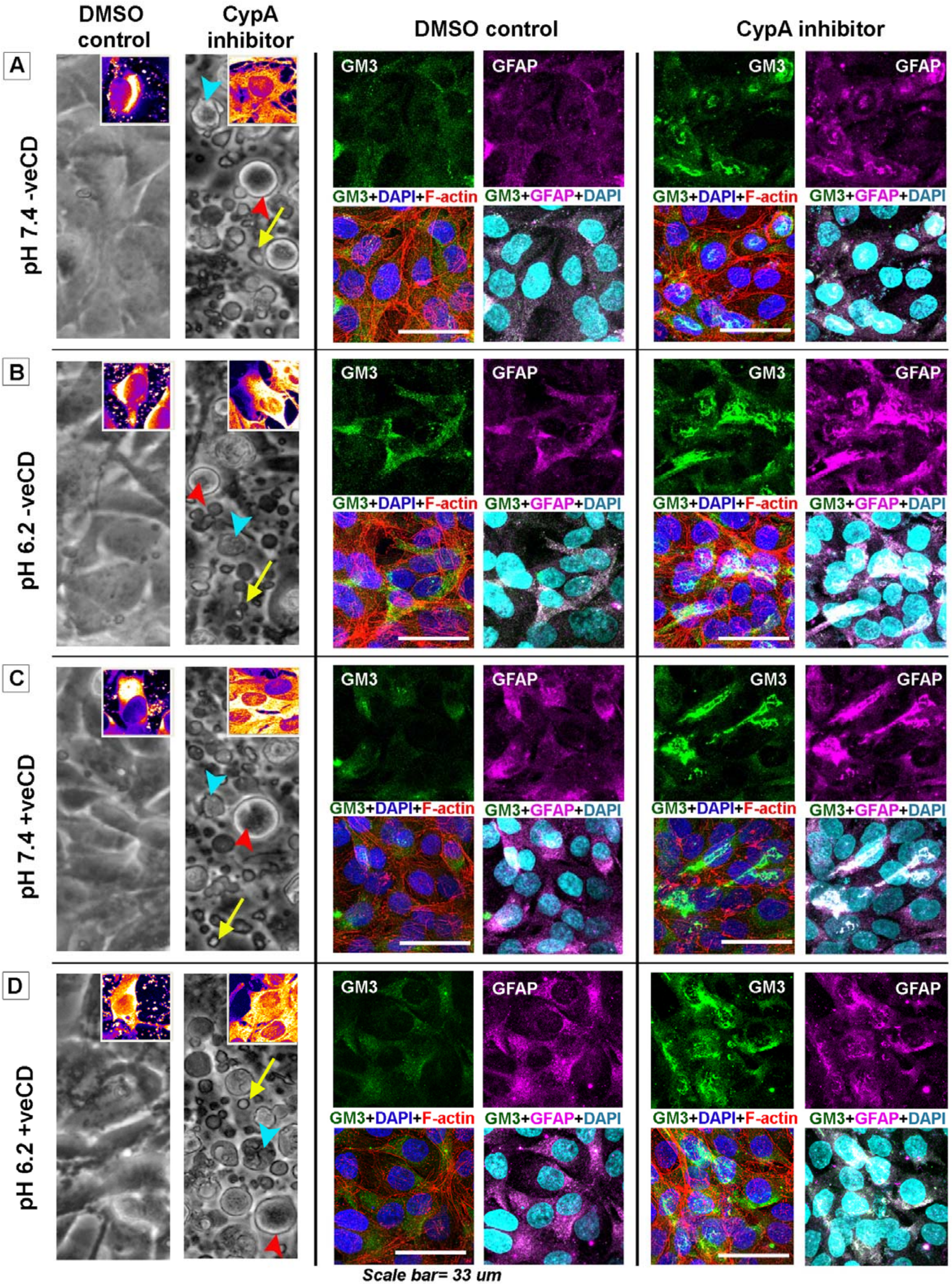
DMSO control
CypA inhibitor
DMSO control
CypA inhibitor
A
B
C
D
pH 7.4 -veCD
pH 6.2 -veCD
pH 7.4 +veCD
pH 6.2 +veCD
GM3
GFAP
GM3+DAPI+F-actin
GM3+GFAP+DAPI
Scale bar= 33 um

S17 Figure
